# Mixed Nd^4+/3+^ and Cluster Magnetism in Hexagonal Perovskite 12R-Ba_4_NdMn_3_O_12−γ_

**DOI:** 10.1021/jacs.6c08795

**Published:** 2026-07-24

**Authors:** Liam A. V. Nagle-Cocco, Madeline G. Port, James Eujin Park, Dennis Nordlund, Oliver Mueller, Stephan Lany, Anna A. Berseneva, Alicia M. Manjón-Sanz, Danielle R. Yahne, Mathias A. Kiefer, Yuntian Li, Benito Ariceaga Gonzalez, Eric N. Coker, Andrea Ambrosini, Ryan A. Klein, Rebecca W. Smaha, Nicholas A. Strange

**Affiliations:** † Stanford Synchrotron Radiation Lightsource, 497525 SLAC National Accelerator Laboratory, Menlo Park, California 94025, United States; ‡ Department of Materials Science and Engineering & Department of Chemistry, 6572 University of Pennsylvania, Philadelphia, Pennsylvania 19104, United States; § 1105 Sandia National Laboratories, Albuquerque, New Mexico 87185, United States; ∥ Materials, Chemical, and Computational Sciences Directorate, National Laboratory of the Rockies, Golden, Colorado 80401, United States; ⊥ Neutron Scattering Division, 6146 Oak Ridge National Laboratory, Oak Ridge, Tennessee 37831, United States; # Geballe Laboratory for Advanced Materials and Department of Applied Physics, 6429 Stanford University, Stanford, California 94305, United States; ¶ Department of Chemistry, University of California, Berkeley, California 94720, United States

## Abstract

Hexagonal perovskite oxides with 12R stacking host well-separated face-sharing octahedral metal trimers with short metal–metal distances, leading in some cases to large degrees of magnetic frustration and cluster magnetism. Introducing magnetic ions in proximity to these trimers can influence the degree of frustration and cluster magnetism, but the extent to which the magnetism can be tuned by varying neighboring metal cations remains an open question. In this work, we test the impacts of using Nd in proximity to Mn trimers in the hexagonal perovskite 12R-Ba_4_NdMn_3_O_12−γ_. Given this stoichiometry, Nd should assume the 4+ oxidation state with a spin state of *S* = 1, which would be the first realization of Nd^4+^ in an oxide environment. Through detailed bulk magnetic, X-ray absorption spectroscopic, and powder neutron diffraction (PND) measurements, we find that Nd^4+^ is realized in this material, but there is also partial reduction to Nd^3+^, which is charge-balanced by O vacancies. Magnetometry measurements indicate an antiferromagnetic ordering temperature *T*_N_ ≈ 16 K, and PND measurements reveal a surprising collinear antiferromagnetic structure with magnetic space group *Pc*2/*m* (no. 10.49 in BNS notation), which has not previously been seen in this class of materials. Our results represent a comprehensive analysis of the structural, electronic, and magnetic properties of 12R-Ba_4_NdMn_3_O_12−γ_, showing the first observation of partial Nd^4+^ in an oxide, and demonstrating that this structural class can host a broad range of magnetic structures which are not easily predicted based on compositional trends.

## Graphical abstract

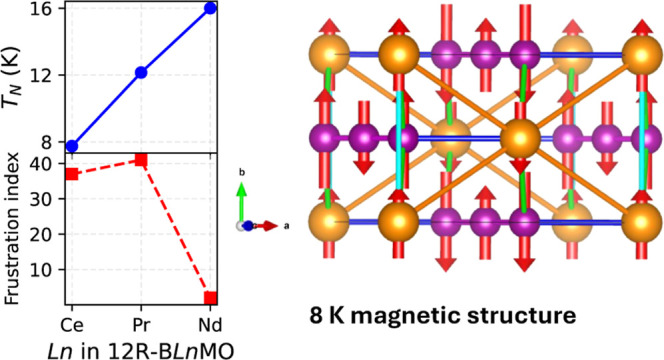

## Introduction

Layered metal oxides of the general formula 12R-Ba_4_ *M*M_3_^′^O_12_ (12R-B*M*M′O) are characterized by a 12-layer stacking of face-sharing M_3_^′^O_12_ trimers, joined by corner-sharing interactions with *M*O_6_ octahedra.^1–12^ This class of materials has received interest in recent years for the possible implications of this crystal structure on magnetism. For instance, the short metal–metal distances within face-sharing trimers often induces very strong intratrimer antiferromagnetic interactions.^9,10,13,14^ These trimers are separated from each other by voids occupied by nonmagnetic Ba^2+^ cations, with this separation leading to intertrimer magnetic interactions manifesting as a magnetic cluster effect.^9,10,14–17^

The 12R-Ba_4_ *M*Mn_3_O_12_ (12R-B*M*MO) subclass is particularly interesting given the large moment on Mn^4+^, which gives rise to a particularly pronounced magnetic cluster effect.^9–11,14^ Materials with various *M* species have been studied, such as niobium,^14^ antimony,^16,17^ tantalum,^17^ hafnium,^11^ or a lanthanoid, Ln.^9,10,18–21^ Magnetic complexity can be introduced by placing a magnetic cation on the corner-sharing *M* site; this has been shown to induce complex magnetic structures including spin canting of the trimer edge Mn^4+^ cations, which shows that while the intratrimer interactions are strong, they are not impervious to external effects.^9,10^ Recently, the 12R-B*M*MO class of materials (*M* = Ce, Nb) has also seen interest from the perspective of thermochemical hydrogen production.^22–25^

There have been very few studies^19,20^ looking at 12R-Ba_4_NdMn_3_O_12_ (12R-BNM), and the existing studies have not investigated magnetic properties, despite the interesting and useful magnetic properties of Nd-containing materials.^27–29^ 12R-BNM is isostructural with the Ln = Ce and Pr materials^9,10^ and would be expected to exhibit a larger moment on the Ln site due to the 4f^2^ occupancy of Nd^4+^ if stoichiometric. However, the Nd^4+^ oxidation state is highly unstable and rarely reported,^30^ so it was suggested that the presence of O vacancies results in full or partial Nd reduction to Nd^3+^,^19^ a phenomenon observed in other isostructural O-deficient phases.^31,32^

In this work, we investigate this material structurally using synchrotron and neutron diffraction and electronically using X-ray absorption spectroscopy (XAS) and density functional theory (DFT). We perform a rigorous magnetic characterization using DC and AC susceptibility measurements, heat capacity measurements, and neutron diffraction in the ordered regime, looking for phenomena such as ferrimagnetic spin canting and the magnetic cluster effect reported in other materials. We investigate the possibility of O vacancies, which have not been reported before in the 12R-Ba_4_LnMn_3_O_12_ compositional series without thermochemical cycling, and look for site-selectivity of vacancy formation, finding none. We investigate the ways in which the Nd material follows, and deviates from, trends in the compositional series as a function of Ln cation. Finally, we examine the possibility of off-centering of the edge Mn^4+^ cations within Mn trimers, and discuss how this may indicate a second-order Jahn–Teller distortion.^33^ Our work significantly extends the range of known structural, magnetic, and electronic phenomena that can occur in 12R-stacked hexagonal perovskites.

## Results

### Crystal Structure and Stoichiometry

12R-BNM crystallizes with the same symmetry as the Ce- and Pr-containing analogs,^9,10^ i.e., a rhombohedral unit cell (*R*3̅*m* space group, refined in the hexagonal setting) in which trimers of face-sharing MnO_6_ octahedra are connected via corner-sharing interactions to LnO_6_ octahedra, with Ba^2+^ cations occupying the voids. This crystal structure is visualized in Figure 1a, based on a Rietveld refinement of room-temperature synchrotron XRD data shown in Figure 1c. Two Ba sites exist at 6*c*(0, 0, *z*) positions (in Wyckoff notation). There is a 3*a*(0, 0, 0) Nd site. Mn sites exist at 3 $b\left(0,0,\frac{1}{2}\right)$ and 6*c*(0, 0, *z*), which correspond to the central and edge MnO_6_ octahedra within the trimers, respectively. There are two O sites at 36*i*(*x*, *y*, *z*). The edge and central MnO_6_ octahedra are nonidentical, exhibiting slightly different octahedral volumes (8.869 Å^3^ and 8.752 Å^3^ respectively). We note that a Ba_4_Mn_3_O_10_ impurity phase forms consistently during 12R-BNM synthesis (Figures 1c and S6). This impurity also forms during synthesis of the Ce- and Pr-containing analogs,^9,10^ suggesting lanthanide loss is common during the synthesis conditions employed here. This is likely due to a combination of the high volatility of lanthanide cations at high-temperatures^34,35^ and the previously reported^36,37^ reactivity of Nd-containing phases with alumina crucibles, which we investigate in SI Section S2.

**Figure 1:**
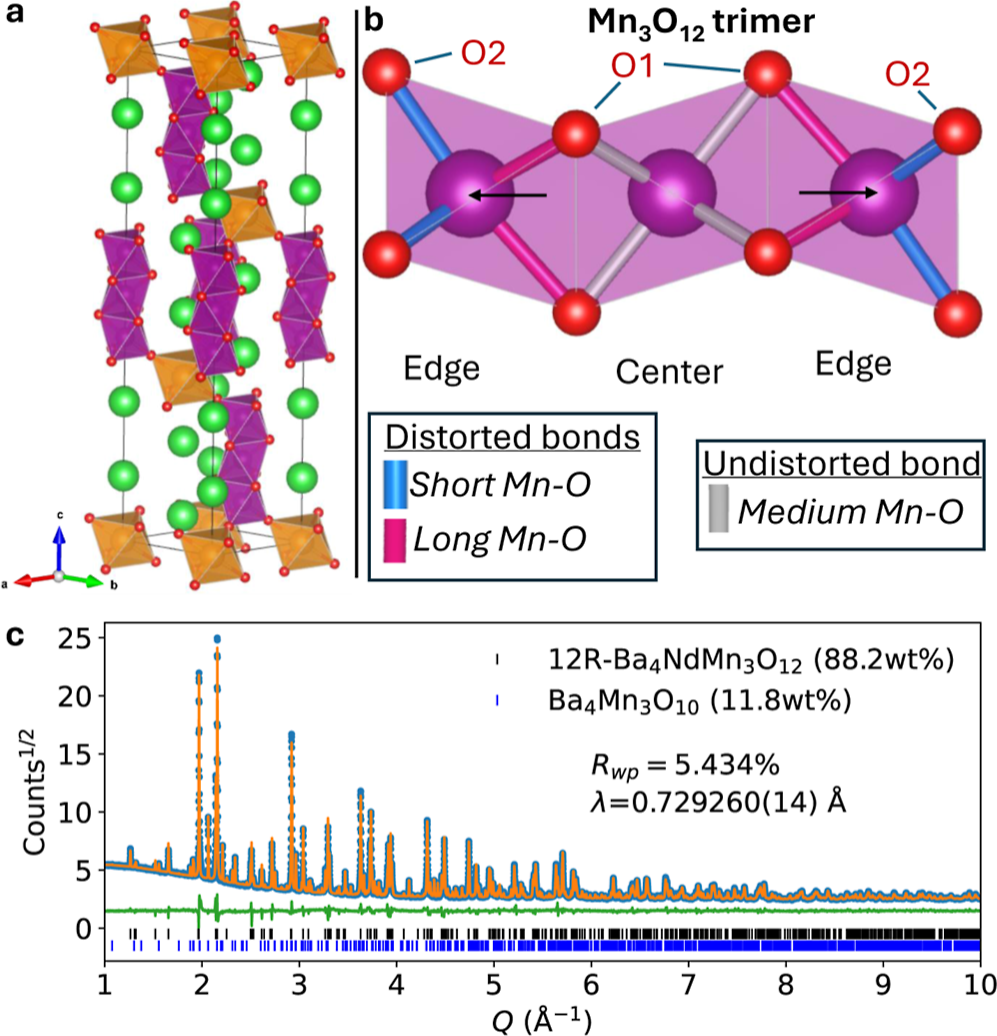
(a) The crystal structure of 12R-Ba_4_LnMn_3_O_12_ (Ln = Ce, Pr, Nd) materials. MnO_6_ octahedra: purple. LnO_6_ octahedra: orange. Oxygen: red. Barium: green. (b) A demonstration of the second-order Jahn–Teller effect observed in the edge MnO_6_ octahedra in 12R-Ba_4_NdMn_3_O_12_ at room-temperature. Black arrows indicate the direction of Mn displacements from octahedral centers. (c) Synchrotron XRD on 12R-BNM measured on beamline 2–1 at room-temperature, with a fit obtained by Rietveld^26^ refinement.

A first-order Jahn–Teller distortion would not be expected^38^ for d^3^ Mn^4+^. Consistent with this expectation, we calculate using vanvleckcalculator^38,39^ that the magnitude of first-order Jahn–Teller distortion (defined as the *Q*_2_ and *Q*_3_ Van Vleck modes^38,40–43^ summed in quadrature) has a value of zero for both edge and central MnO_6_ octahedra. However, a second-order (or pseudo) Jahn–Teller distortion may sometimes become energetically favorable for d^3^ Mn^4+^ ^33^ and has been reported previously.^44,45^ While the central MnO_6_ octahedron also has zero bond length distortion (as defined by the bond length distortion index^46^), the edge MnO_6_ octahedra have a nonzero bond length distortion index of around 0.0393(15) at room-temperature. This value is larger than that reported for undistorted Mn^2+/3+^ in cryptomelane,^47^ which was 0.01320(12), and comparable to the value 0.05463(14) reported for a first-order Jahn–Teller distortion in NiO_6_ octahedra.^48^ This bond length distortion is due to a subtle Mn^4+^ cation off-centering, with the Mn^4+^ cation occurring at a distance of ∼0.14 Å from the mean position of the six oxygen anions in the edge MnO_6_ octahedra. While Coulombic repulsion between the Mn–Mn cations may play a role, this would also be consistent with a second-order Jahn–Teller distortion on the edge MnO_6_ octahedra (Figure 1b), as has been suggested for the similar trimer-containing Ba_3_NbMO_8.5_ (*M* = Mo, W).^49^ While such an effect has not yet been reported in the 12R-B*M*MO compositional series, inspection of the edge MnO_6_ octahedra in the reported room-temperature structures of 12R-BCM and 12R-BPM^9^ indicates an identical off-centering distance of ∼0.14 Å, suggesting the phenomenon is common to the series.

We calculate the bond valence sum using eq 4 (described in Methods) as an empirical proxy for oxidation state based on bond lengths. For Mn1 and Mn2, these give valences of 4.22(5) and 4.19(4), respectively, whereas for Nd, the calculation gives a valence of 3.67(6). The Mn values are broadly consistent with an oxidation state of 4+, with deviation attributable to local effects such as strain associated with face-sharing octahedral interactions and the aforementioned cation off-centering. The deviation from 4+ in the case of Nd is less likely to be attributable to strain effects given the corner-sharing nature of the octahedral interactions and more likely a consequence of partial reduction to 3+ as will be discussed later.

12R-Ba_4_ *M*Mn_3_O_12_ (*M* = Ln, Nb) materials can host O vacancies, as evidenced by their successful deployment as materials for thermochemical hydrogen production,^22–25^ and thermogravimetric analysis suggests that 12R-BNM is more readily reduced than the Pr analog (Figure S13), likely due to the low stability of the Nd^4+^ oxidation state, suggesting the presence of O vacancies. To our knowledge, oxygen vacancy content in these materials has not been quantified by scattering measurements. X-rays are relatively insensitive to oxygen vacancies in the presence of elements such as Nd with high proton numbers, so we assume fully occupied O sites in our synchrotron XRD Rietveld refinement. However, neutrons are sufficiently scattered^50^ by oxygen such that we can refine vacancy content at these positions. We perform a Rietveld refinement against the neutron diffraction data at 200 K (Tables S2 and S3; Figure S16) which indicates 3.66(14)% O vacancy content across both sites (we saw no meaningful improvement in *R*_wp_ from using different vacancy parameters for each site), corresponding to a true stoichiometry of 12R-Ba_4_NdMn_3_O_12−γ_ where γ = 0.440(16).

To understand the origin of these vacancies, we calculated O vacancy formation energy using DFT + U for the Ce-, Pr-, and Nd-containing 12R-Ba_4_LnMn_3_O_12_ materials (SI Table S8). We found that O vacancy formation energies for BCM and BPM are 3.03 and 2.76 eV, respectively. For BNM, the vacancy formation energy was far lower at 1.75 eV. This is consistent with our finding of non-negligible vacancy content in BNM, compared with previous reports of zero/negligible vacancy content in BCM and BPM.^9,10^

### Electronic Properties of 12R-BNM

In charge-balanced 12R-Ba_4_LnMn_3_O_12_ (Ln = Ce, Pr), assuming stoichiometric oxygen content, we would expect both Mn^4+^ and Nd^4+^. However, given the rarity of the Nd^4+^ oxidation state^30^ and the observation by neutron diffraction of 3.66(14)% O vacancy content averaged over both sites (Figure S16), there must be some metal reduction to charge balance. Using XAS measurements and DFT + U calculations, we determine whether this charge balancing occurs via reduction of Nd^4+^ toward Nd^3+^, reduction of Mn^4+^ toward Mn^3+^, or a combination of both. The charge-balancing mechanisms, and resulting cationic oxidation states, in 12R-BNM must be understood in order to understand other properties of the material, such as its magnetic properties and potential water-splitting behavior (by analogy with 12R-BCM^22^).

We first discuss the XAS data. We have measured both hard XAS (Mn K-edge, Nd L-edge, Ba L-edge) and soft XAS (O K-edge, Mn L-edge, Nd M_4,5_-edge), as shown in Figure 2. We use as a reference the extensive XAS characterization that has already been performed on the Ce- and Pr-containing analogues.^10^

**Figure 2:**
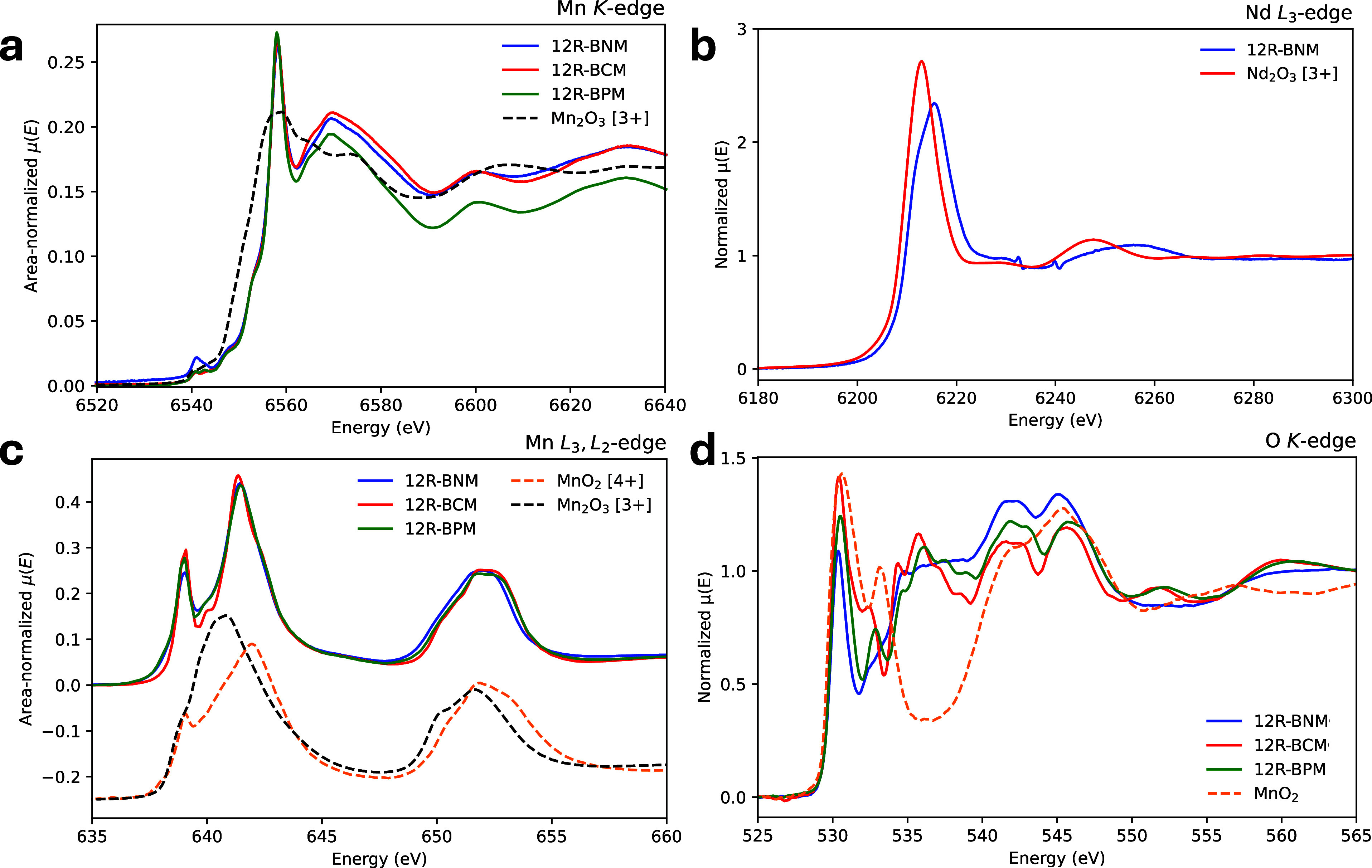
Background-corrected and normalized hard XAS of 12R-BNM compared to 12R-BCM, 12R-BPM, and relevant oxide reference states. (a) Mn K-edge hard XAS spectra, with area-normalization between 6555 and 6560 eV. (b) Nd L_3_-edge hard XAS spectra. (c) Mn L_3_ and L_2_-edge soft XAS spectra, with area-normalization between 641 and 647 eV. (d) O K-edge soft XAS spectra. The data for 12R-BPM and 12R-BCM was previously published by Regier et al.^10^

Figure 2a shows the Mn K-edge XAS of the Ln = Ce, Pr, and Nd members of the 12R-Ba_4_LnMn_3_O_12_ compositional series, along with Mn^3+^ and Mn^4+^ references. This edge is typically dominated by the dipole-allowed 1s → 4p transition. The Mn K-edge spectra of the hexagonal perovskite materials display a sizable upshift in edge energy relative to the Mn^3+^ reference, indicative of oxidation consistent with the expected Mn^4+^ oxidation state. Among the three 12R hexagonal perovskites, the XAS appears very similar, with the only significant difference being the presence of a significantly larger prepeak feature at ∼6540 eV for the Ln = Nd member of the compositional series. Such prepeaks in transition metal oxide octahedra are typically the consequence of: (1) dipole-forbidden quadrupole (1s → 3d) transitions and (2) dipole-allowed transitions via 3d/4p mixing. The latter would occur as a consequence of octahedral distortion, which in this case could be due to one or both of the edge Mn off-centering and the O vacancies. However, as the prepeak is much larger in 12R-BNM than 12R-BCM or 12R-BPM, it is likely due to the O vacancies rather than the off-centering effect, which also occurs in those two materials with approximately equal magnitude.

As for the Mn L-edge, shown in Figure 2c, we see by comparing the Mn^4+^ and Mn^3+^ standards that the L_3_ feature exhibits resolvable multiplet splitting for the Mn^4+^ case but not for the Mn^3+^ case. This splitting is clearly present for all of the three hexagonal perovskites studied here. The 12R-BNM data appear slightly broader compared with the other two hexagonal perovskites, in particular 12R-BCM. This may be due to a combination of factors, including the local heterogeneity caused by O vacancies. The presence of trace Mn^3+^ cannot be ruled out on the basis of these data, though comparison to 12R-BPM (which has not been reported to show O vacancies) suggests this is unlikely. We should also note that 12R-BNM was measured on a different beamline from the other two hexagonal perovskites (see Methods), which could result in subtle changes in feature broadening.

The key takeaway from the Mn edge XAS data presented here is that Mn is primarily in the same oxidation state in 12R-BNM as it is in 12R-BCM and 12R-BPM, i.e., Mn^4+^.

Due to the rarity of the Nd^4+^ oxidation state,^30^ we have no standard for it, so we can only judge the Nd-oxidation state relative to Nd^3+^ standards (Nd_2_O_3_). We show the Nd L_3_-edge in Figure 2b, which typically corresponds to a 2p_3/2_ → 5d transition in Ln spectra. For our Nd^3+^ standard, and for similar Nd^3+^-containing materials,^51–54^ the L_3_ edge is typically a single white line, but we see what appears to be two superposed overlapping features at different energies. This is even more clear in the L_2_ edge (Figure S23a). Although there are no Nd^4+^ oxide standards available to us, tetravalent Nd halides^30,55^ also exhibit an L_3_-edge dominated by a single prominent white line. If we assume the same is true of tetravalent Nd oxides, our dual-feature spectrum can be interpreted as likely a superposition of trivalent and tetravalent white lines, offset by ∼3 eV at the L_3_-edge. However, we also note that other tetravalent Ln–O environments (Ln = Ce, Pr, Tb) do not exhibit single white lines, but rather clear multiplets.^56–64^ Further, the asymmetric Nd L_3_-edge we measure resembles that reported for tetravalent Tb in O environments.^59,64^ Given this, we find that the Nd L_3_-edge XAS data on 12R-BNM are inconsistent with fully trivalent Nd, but could be consistent with either a fully tetravalent or a mixed trivalent/tetravalent system. Crucially, we also see an upshift of edge energy relative to the Nd^3+^ reference, which supports the interpretation of oxidation relative to the trivalent state. We conclude from the Nd L_3_-edge data that Nd-oxidation is most likely to be the mechanism of charge balancing O vacancies in 12R-BNM.

We also performed XAS measurements at the Nd M_4,5_-edge (Figure S22) in 12R-BNM. Whereas the multiplet structure at the L_3_ edge (2p_3/2_ → 5d) is very sensitive to valence, due to the extended and hybridized nature of 5d orbitals, the localized nature of the 3d_5/2,3/2_ → 4f final state makes the multiplet structure of the M_4,5_ edge relatively invariant with valence.^65^ However, while the multiplet structure does not vary much with valence, the absolute energies of features does vary. The relatively sharp features reported for single-valence Nd,^65–67^ particularly at the M_4_-edge (∼1000 eV) contrast with the smeared features in our measured spectrum (Figure S22). This would be consistent with mixed-valence Nd, i.e., via a superposition of two near-identical spectra with a constant offset.

To explain the XAS data on Mn and Nd, we performed DFT + U calculations of the density-of-states shown in Figure 3. This shows a band gap[Fn fn1] of 0.66 eV, far smaller than the 1.40 eV reported for BPM or 1.96 eV for BCM,^10^ indicating a trend of band gap with Ln in this compositional series. Further, it shows the reason for the collapse in band gap size for the Nd-containing material is the significant decrease in minimum energy of states associated with the Nd f orbital occupancy relative to the valence band minimum (VBM). This means that Nd is far more susceptible to reduction than Mn, which explains why the Nd reduces more readily in 12R-BNM compared with Mn.

**Figure 3:**
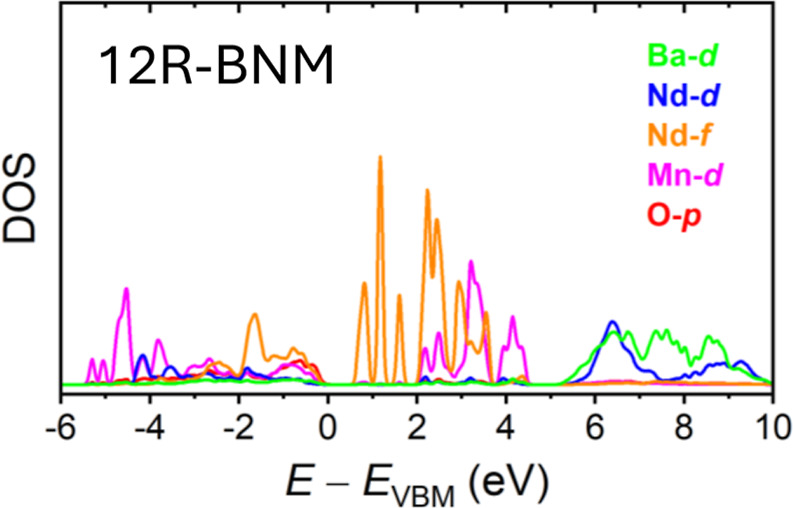
DFT + U calculated partial density of states (DOS) of 12R-BNM (assuming no oxygen vacancies) in the vicinity of the band gap reveals insulating behavior with a band gap of ∼0.66 eV. A Gaussian smoothing with σ = 0.05 eV was applied to this DOS.

Figure 2d shows the O K-edge, which corresponds to 1s → 2p transitions and approximates a *p*-projected partial density-of-states, comparing 12R-BNM, 12R-BPM, and 12R-BCM with a MnO_2_ standard. These spectra are rich with features, due to both Mn–O and Nd–O contributions, and the two different Mn–O environments. These features will be discussed below.

The low-energy features of the O K-edge, between 529 eV and ∼535 eV, are characteristic of Mn-containing oxides and occur due to hybridization between Mn 3d orbitals with O 2p orbitals.^68–72^ The lower-energy and higher-energy features in this range are due to hybridization with 3d t_2g_ orbitals and e_g_ orbitals, respectively.^68^ The prominent 3d *t*_2*g*_ hybridization feature at ∼531 eV is reminiscent of the characteristic Mn^4+^ feature absent in LaMn^3+^O_3_ but present in SrMn^4+^O_3_.^73^ The feature’s decreased intensity for 12R-BNM compared with 12R-BCM and 12R-BPM indicates reduced O 2p-Mn 3d overlap, likely due to octahedral angular distortion stemming from increased O vacancy content.

Hybridization with Mn orbitals also contributes to the more delocalized, higher-energy features of the O K-edge; the features between 540 and 547 eV are attributed to O 2p-Mn sp hybridization,^10^ by comparison with prior works.^72^

Meanwhile, the intermediate features in the O K-edge spectrum, between 534 eV and ∼540 eV, are attributed to the LnO_6_ octahedra. Here, the 4f orbitals are typically highly localized, and though there are some reports of O 2p-Ln 4f hybridization, these are unlikely to contribute significantly to the observed features.^64,74,75^ We therefore suspect these features are primarily due to O 2p-Ln 5d hybridization at lower energy, with 5d states split into pi t_2g_ and e_g_ components. There will also likely be weaker 6s/6p hybridized contributions at higher energy within this intermediate range. Again, we note that the 12R-BNM features in the intermediate region of the O K-edge appear smeared and less resolvable compared to 12R-BCM and 12R-BPM, likely due to O vacancy disorder in NdO_6_ octahedra.

Finally, we note that there is essentially no difference in the Ba L-edge between 12R-BNM and 12R-BCM (Figure S24), likely due to the highly stable nature of the Ba^2+^ oxidation state.

### Bulk Magnetic Properties and Heat Capacity

Variable-temperature magnetometry measurements on heating are shown in Figure 4 and contain two features. First, there is a broad bump around 120 K that is clearly present at a low-field but suppressed at a higher field. This bump marks the onset of a bifurcation between the field-cooled and zero-field-cooled susceptibility data. While such a divergence may be interpreted as evidence for spin-glass behavior,^76–79^ AC susceptibility shows no frequency-dependence is resolved at this transition temperature within the temperature resolution and frequency range of our measurements (Figure S10), making a conventional spin-glass assignment unlikely. Below the bump, the field-dependence exhibits a low-field, high-susceptibility region, which is suppressed above the bump (see the upper left inset in Figure 5); a similar effect is reported for the analog materials with Nb and Ta-substituted on the Nd site.^17^ The impurity phase, Ba_4_Mn_3_O_10_, exhibits a very similar broad feature,^13^ but we do not believe this feature is due to the impurity phase as we cannot see the antiferromagnetic transition that occurs in Ba_4_Mn_3_O_10_ around 40 K. It is far more likely that this feature is intrinsic to the 12R-BNM sample, as a result of similar phenomena to that which occur in the impurity phase. As shown in Figure S7, the temperature of this bifurcation transition decreases with increasing field and is near-suppressed by *H*_ext_ = 50 kOe. A similar feature is also observed in the magnetometry data for the Pr-containing analog (12R-BPM)^9^ and 12R-Ba_4_Fe_3_NiO_12_.^2^ Using low-temperature synchrotron refinement, we see no indication that this feature is related to a structural transition (Figure S15).

**Figure 4:**
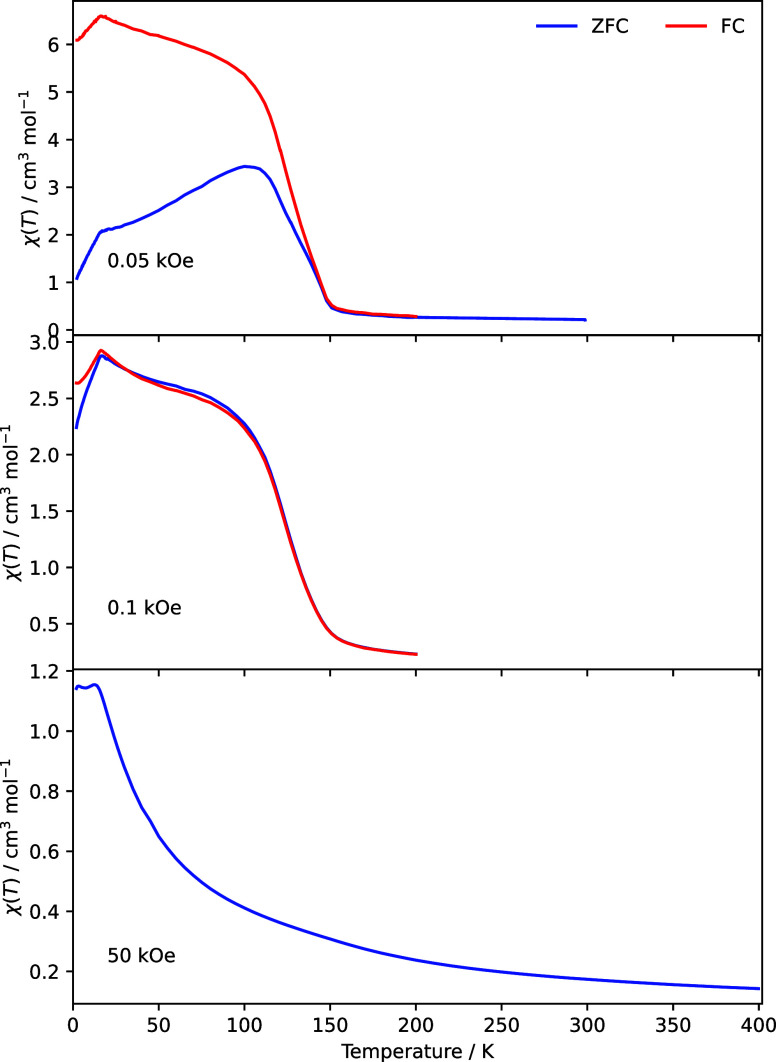
DC magnetic susceptibility as a function of temperature under applied fields of 0.05 kOe, 0.1 kOe, and 50 kOe. For FC-W (field-cooled, measured on warming) measurements, a field equal to the subsequent field on heating was used during the cooling before measurement.

**Figure 5:**
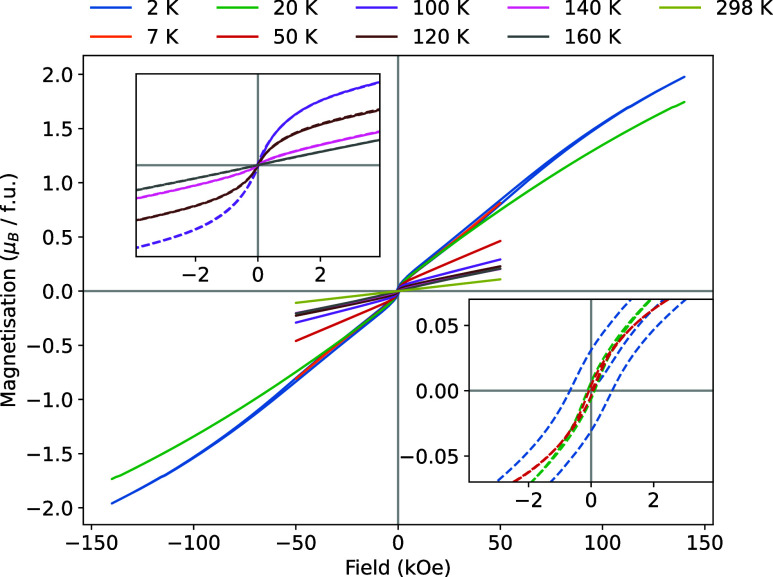
DC magnetic moment as a function of an applied field. Upper left inset shows the disappearance of the low-field high-gradient component of the magnetization on heating through the 120 K transition. Lower right inset shows the suppression of hysteresis on heating at cryogenic temperatures.

Second, we see a clear antiferromagnetic ordering on further cooling to around 17 K, which we interpret as the onset of long-range magnetic ordering. In the variable-field data, Figure 5, cooling through this transition approximately corresponds to the emergence of low-field magnetic hysteresis, as shown in the lower-right inset. This hysteresis has a magnitude of ∼0.03 μ_B_/f.u. at 2 K in the ordered regime, but some hysteresis persists into the paramagnetic regime, with a value of ∼0.003 μ_B_/f.u. at 20 K, suggesting that there may be some local spin canting, which does not show long-range order. We note a similar small hysteresis was reported for antiferromagnetic Ba_4_Mn_3_O_10_,^13^ although we attribute the hysteresis at least partly to the 12R-BNM as we posit that the phase fraction of Ba_4_Mn_3_O_10_ is too low to give rise to such hysteresis.

At temperatures above these two features, the sample appears paramagnetic. However, the magnetic susceptibility in this paramagnetic regime exhibits nonlinearity with curvature $\frac{d^{2}\chi \left(T\right)}{dT^{2}}<0$ , as shown in Figures S8 and S34, indicative of a positive (paramagnetic) temperature-independent component. These data were therefore fit with a modified Curie–Weiss equation,^80^ defined thus

1 $$\chi^{-1}\left(T\right)=\frac{T-T_{CW}}{C+\chi_{0}\left(T-T_{CW}\right)}$$

where *T* is the temperature, χ is the magnetic susceptibility, *C* is the Curie constant, *T*_CW_ is the Weiss temperature, and χ_0_ is the temperature-invariant component. The results of this fitting are dependent on the fitting range used, so the largest possible temperature range within which the temperature-dependence of susceptibility can be fit with eq 1 was used as indicated in Figure S35. Consequently the Curie–Weiss fit was performed in the temperature range 270 to 400 K, with the results tabulated in Table 1 with comparison to 12R-BPM and 12R-BCM.

**Table 1:** Results of Fitting the Modified Curie–Weiss Equation (eq 1) to the High-Temperature Inverse Susceptibility Data for 12R-Ba_4_LnMn_3_O_12_ (Ln = Ce, Pr, Nd)^a^

Ln in 12R-BLnMO	*T*-range (K)	χ_0_ (cm^3^ mol^–1^)	*C* (cm^3^ K mol^–1^)	*T*_CW_ (K)	μ_eff_ (μ_B_/f.u.)
Ce^4+^	250–350	+0.00398	2.2(13)	–128(120)	3.1(9)
Pr^4+^	250–350	–0.00533	140(70)	–522(197)	9(2)
Nd^3+/4+^	270–400	+0.0422(7)	42.5(5)	–23(2)	5.24(3)

a The fits are shown in Figure S36 for 12R-BNM. The results for 12R-BPM and 12R-BCM are taken from ref 9; for the conversion from cgs units in that paper to SI units, we use 1 emu Oe^–1^ = 1 cm^3^. All fitting was performed on data measured in an external field of 50 kOe. The *T*-range column indicates the temperature range in which inverse susceptibility data were used for fitting.

From fitting the Curie–Weiss equation, we can calculate moment per formula unit. This requires knowledge of the molar mass, which depends on the O vacancy content. We note that ignoring the Ba_4_Mn_3_O_10_ impurity phase does not meaningfully impact the calculated Bohr magneton per formula unit (Figure S37). Using the refined O vacancy content of 3.66(14)%, corresponding to γ = 0.440(16) in 12R-Ba_4_NdMn_3_O_12−γ_, we calculate μ_eff_ = 5.24(3) μ_B_ per formula unit. This is far smaller than the expected free-spin effective magnetic moment for 12R-BNM, which is ∼7.40 μ_B_/f.u. (Table S6) including orbital contributions for Nd. We note that we assumed Nd^4+^ in our calculations here, as orbital contributions to μ_eff_ make Nd^3+^ and Nd^4+^ essentially indistinguishable (3.62 μ_B_ vs 3.58 μ_B_, respectively).

The measured moment of the Ce-containing analog, 3.1(9) μ_B_, is also smaller than the calculated moment ∼6.71 μ_B_, and a similar discrepancy between measured and predicted paramagnetic moment has been observed in 4H–BaMnO_3–*x*_ and 4H–BaMn_0.5_Sr_0.5_O_3–*x*_ by Adkin and Hayward^81^ and 12R-Ba_4_Mn_3_NbO_12_ by Nguyen et al.^14^ A similar behavior is observed in off-stoichiometric 12R-Ba_4_Hf_0.8_Mn_3.2_O_12_ but is shown to disappear by 232 K.^11^ The proposed origin of this behavior is the magnetic cluster effect, whereby local antiferromagnetic correlations prevent all moments in the sample from aligning with an external magnetic field.

As we know from XAS that Mn exists in the 4+ oxidation state, and that the effective moment of Nd^3+^ and Nd^4+^ is essentially equal, we can calculate μ_eff_ = 5.27 μ_B_ per formula unit assuming entirely antiferromagnetic clusters with an effective moment of $S_{eff}=\frac{3}{2}$ (Table S6). This calculated moment occurs very close to our experimentally derived value, consistent with magnetic clustering as the origin of our reduced paramagnetic effective moment, and also with the absence of Mn charge disproportionation, which would significantly impact the experimentally derived moment. In contrast, if we consider the alternative trimer scenario where one Mn is reduced to Mn^3+^ (*S*_eff_ = 1), we would get μ_eff_ = 4.59 μ_B_, which is far smaller than our measured value.

As with 12R-BCM and 12R-BPM, we obtain a negative Weiss temperature for 12R-BNM (Table 1) of – 23(2) K. This is indicative of local antiferromagnetic correlations within the paramagnetic regime. We observe, however, a far smaller value of the Weiss temperature compared with the 12R-BCM and 12R-BPM materials, which are an order of magnitude larger. A calculation of the magnetic frustration index^80,82–88^ of the material is performed as

2 $$f=\left|\frac{T_{CW}}{T_{N}}\right|$$

where *T*_N_ ≈ 16 K is the magnetic ordering Néel temperature. This gives rise to *f* ≈ 2 for 12R-BNM, indicative of the absence of significant magnetic frustration. In contrast, the values for 12R-BCM and 12R-BPM are ∼37 and ∼41 respectively, indicative of substantial frustration in those materials. This suggests subtle differences in the local magnetic behavior in 12R-BNM compared with the Ce- and Pr-containing analogs, likely related to the suppression of the strength of local antiferromagnetic correlations.

We also obtain from this Curie–Weiss fitting a slightly positive temperature-independent component χ_0_. We would typically expect χ_0_ to be negative due to intrinsic diamagnetism, and so a positive value indicates that there is a temperature-independent paramagnetic component with a greater magnitude than the intrinsic diamagnetism. Nd^4+^ is a non-Kramers’ ion and therefore has a tendency to exhibit Van Vleck paramagnetism, and this is the most likely origin of this positive χ_0_. This is supported by the fact that a positive temperature-independent component was also observed for the Ce^4+^-containing (spin-0; also a non-Kramers’ ion) analog (12R-BCM), but not the Pr^4+^-containing (spin- $\frac{1}{2}$ ) analog (12R-BPM).^9^ Such Van Vleck paramagnetism is common among lanthanoid-containing materials^83,85,89–93^ and its presence indicates Nd is at least partially in its 4+ oxidation state. However, a positive χ_0_ is also reported for the Nb-containing analog,^14^ which does not contain non-Kramers’ ions, which authors attribute to incomplete quenching of local magnetic order associated with the Mn_3_O_12_ trimers, although this would not explain the negative χ_0_ obtained for the Pr-containing material.

Heat capacity data were collected under a range of incident magnetic fields between 0 and 14 T (Figure S38). The low-temperature heat capacity data were found not to exhibit expected Debye behavior (Figure S39), so the magnetic heat capacity data were obtained using an empirical subtraction detailed in the Methods section. There is a broad transition between 12 and 18 K, shown in the upper panel of Figure 6, consistent with the magnetic transition observed via DC susceptibility measurements. This transition is found to be highly sensitive to an external magnetic field, being suppressed progressively as field increases to 14 T. This is in contrast to the lack of magnetic field dependence of the transition observed in 12R-BCM and 12R-BPM.^9^

**Figure 6:**
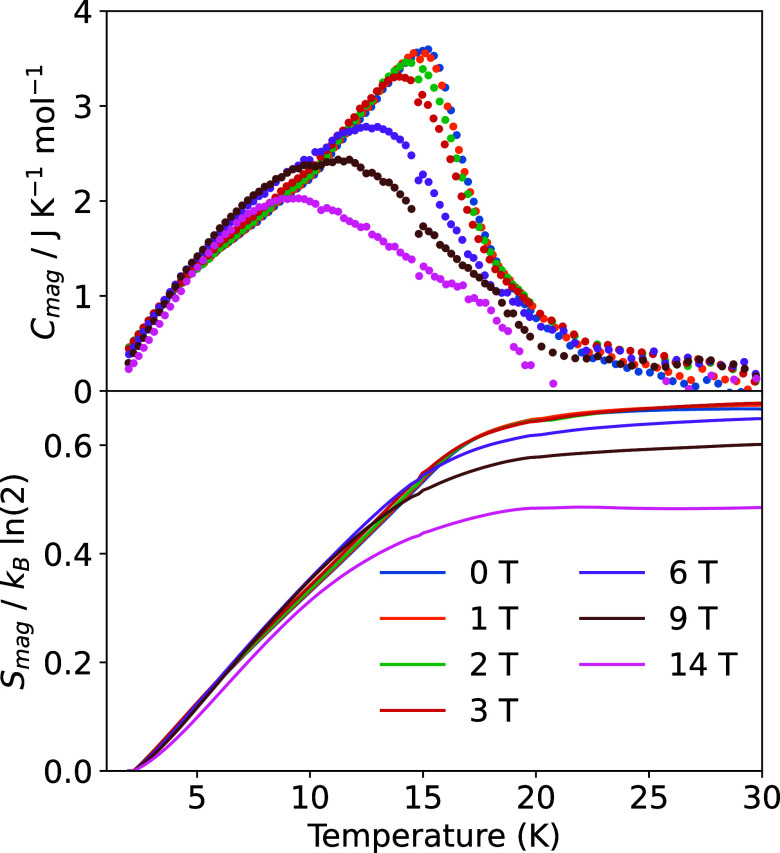
Magnetic contribution to heat capacity, *C*_mag_, as a function of temperature for a range of magnetic fields, compared with the calculated magnetic entropy. The origin of the anomalous data point at many fields around 15 K is not known but is likely an instrumental artifact.

We calculate the magnetic entropy, Δ*S*_mag_, released through this transition in the lower panel of Figure 6. We see a larger entropy change than was observed^9^ for 12R-BCM (where the Ln cation is nonmagnetic Ce^4+^) indicating some contribution of the magnetic Nd to the entropy change. However, we see far less than the theoretical maximum entropy change for this material (∼5.25 *k*_B_ × ln 2 assuming Nd in equal parts 3+ and 4+; derived in SI Section S12) and also less than that observed experimentally for 12R-BPM^9^ despite Pr^4+^ having a smaller moment than Nd^4+^.

The large discrepancy between the theoretical entropy release through the transition and the much smaller observed entropy release suggests that the Nd sublattice likely does not strongly participate in the magnetic order that exists below the ordering transition. However, the entropy is larger than that expected purely for spin clusters, and so the Nd does at least partially participate in the magnetic ordering. The strong suppression of the heat-capacity anomaly in the Nd compound with applied field arises because Nd^3+/4+^ carries a magnetic 4f moment that can be partially polarized by the field above the ordering temperature, reducing the entropy released at the transition and possibly affecting the Mn ordering. In contrast, Ce^4+^ is nonmagnetic (4f^0^), so the peak in the heat capacity of 12R-BCM at the transition remains largely unaffected by field. To test this hypothesis, future work may consider studying the magnetic structure of 12R-BNM as a function of the external field using neutron diffraction.

### Low-Temperature Magnetic Structure

Time-of-flight powder neutron diffraction (PND) was used to determine the magnetic structure of 12R-BNM that emerges below *T*_N_ ≈ 16 K. Figure S16 shows the results of a Rietveld refinement against the PND data at 200 K, at which temperature neither 12R-BNM nor the impurity phase Ba_4_Mn_3_O_10_ exhibit long-range magnetic order. Consequently, the PND data can be fit using the same structural models as are used in the room-temperature synchrotron refinement (Figure 1c). On further cooling to 8 K, a number of additional peaks emerge, which we attribute to magnetic ordering.

Figure 7 shows the magnetic Bragg peaks that emerge at low temperatures. One set of magnetic Bragg peaks emerge by cooling to 20 K. These we attribute to the Ba_4_Mn_3_O_10_. We fit these peaks using a |k⃗|=0 propagation vector and the *Pb*′*c*′*a* (no. 61.436) magnetic space group, as described using the Belov–Neronov–Smirnova (BNS) notation,^95^ obtaining a magnetic structure qualitatively identical to that obtained by Zubkov et al.^13^ for Ba_4_Mn_3_O_10_. A Rietveld refinement at 20 K, including the magnetic Ba_4_Mn_3_O_10_ phase, is shown in Figure S20. Interestingly, we also see some scattering intensity associated with the [021] (*Q* ≈ 1.1 Å^–1^) Bragg peak at 60 K, which is above the ordering temperature for Ba_4_Mn_3_O_10_ reported by Zubkov et al.^13^

**Figure 7:**
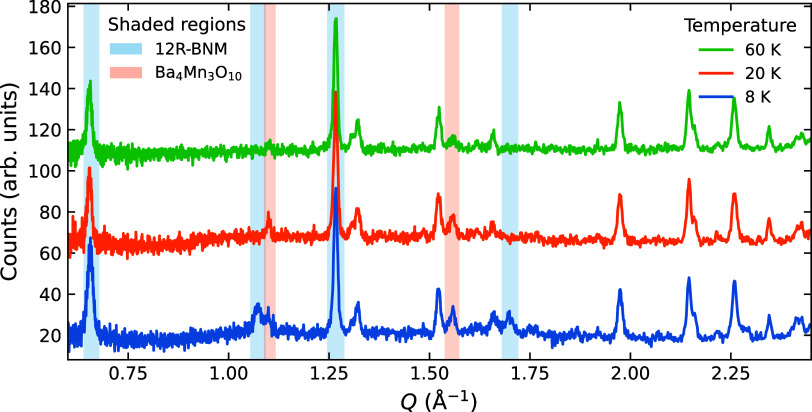
PND data on 12R-BNM measured on POWGEN^94^ at 60 K, 20 K, and 8 K, as viewed over the *Q*-range in which magnetic peaks emerge. Shaded regions indicate the emergence of magnetic Bragg peaks, with blue shading attributed to 12R-BNM and orange shading to Ba_4_Mn_3_O_10_.

The second set of magnetic Bragg peaks emerge on cooling from 20 to 8 K and are attributable to the 12R-BNM phase. Indexing these peaks using k search implemented in fullprof^96^ gives rise to several possible propagation vectors, with the most promising being *k⃗* = (0, ^1^/_2_, 1). isodistort^97,98^ was used to generate all possible irreducible representations of the magnetic modes consistent with this propagation vector, and these were refined against the diffraction pattern in a four-phase fit, with the nuclear 12R-BNM phase and both the nuclear and magnetic phases of Ba_4_Mn_3_O_10_.

This trial and error approach led to the structure solution using this propagation vector *k⃗* = (0, ^1^/_2_, 1) and magnetic space group *Pc*2/*m* (no. 10.49), a structure with just three magnetic degrees of freedom, all of which are magnetic moments contained within the *b*-axis (perpendicular to the Mn–Mn–Mn trimers). We perform Rietveld analysis in which the O occupancy was fixed to the value obtained by Rietveld refinement of the 200 K neutron diffraction data set (Table S2). The fit is shown in Figure 8 magnifying the magnetic peaks; a full refinement is shown in Figure S21.

**Figure 8:**
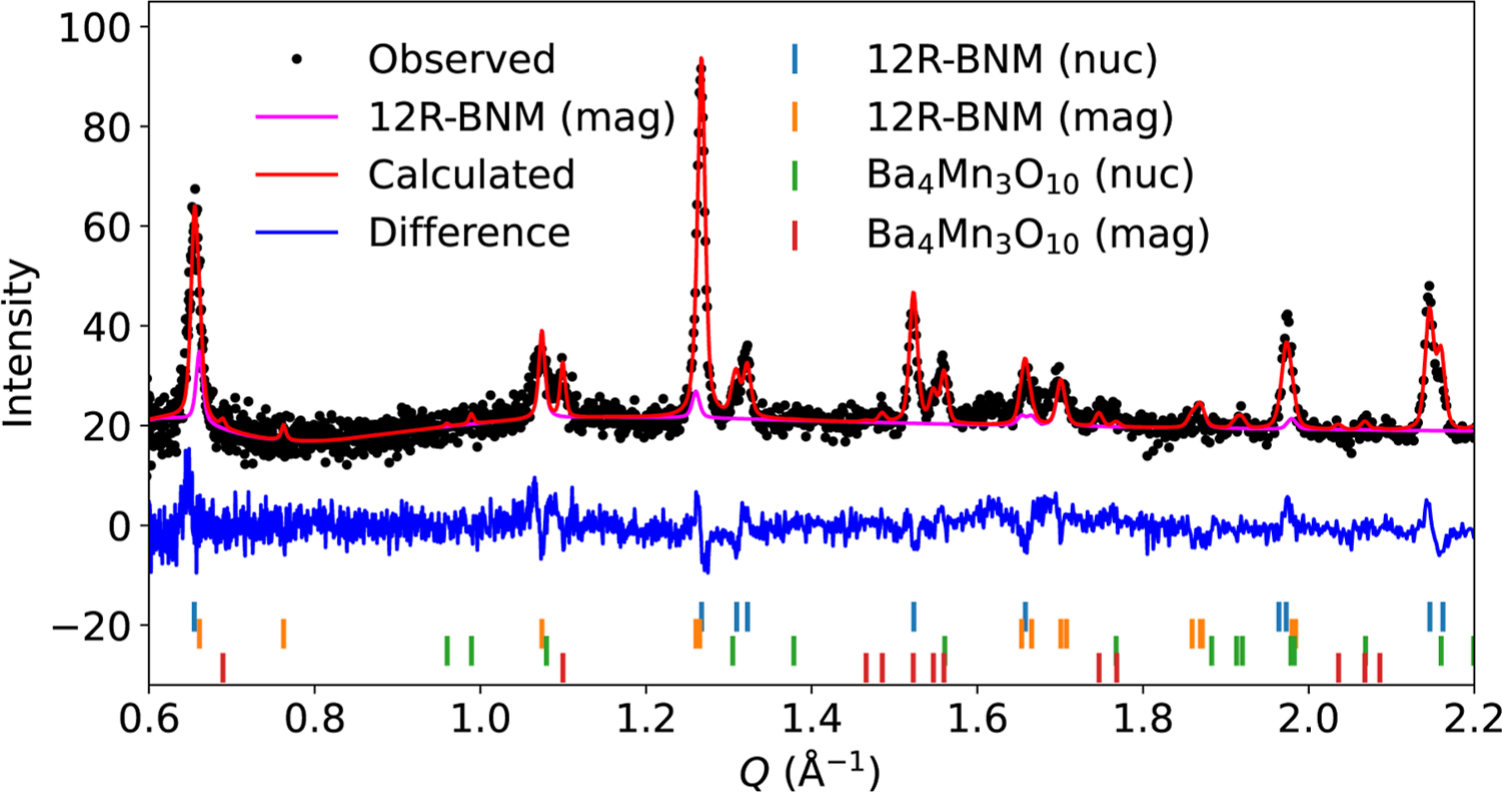
PND data from POWGEN at 8 K, fit by Rietveld refinement using four phases, two nuclear and two magnetic. The two nuclear phases are 12R-BNM (*R*3̅*m* in the hexagonal setting) and Ba_4_Mn_3_O_10_ (*Cmca*, using the crystal structure reported by Zubkov et al.^13^). The magnetic phases are for Ba_4_Mn_3_O_10_ (*Pb*′*c*′*a*, or no. 61.436) and our newly reported magnetic structure for 12R-BNM (*Pc*2/*m*, or no. 10.49). The calculated data for the 12R-BNM magnetic phase are shown, to demonstrate the location of the magnetic peaks. In this refinement, the O vacancy concentration was fixed to that obtained from Rietveld refinement of the neutron diffraction data at 200 K (Table S2; Figure S16). Vertical tick marks indicate the position of Bragg reflections.

This structure is antiferromagnetic, consistent with the dependence of susceptibility on temperature through the transition as shown in Figure 4. The refined magnetic moment magnitudes for Mn^4+^ are 0.99(10) μ_B_ for the edge cation and 0.89(11) μ_B_ for the central cation. These are both less than would be expected, though this can be explained by the continuation of thermal fluctuations suppressing the average moment. For the Nd moment, we refine a moment of 0.75(14) μ_B_. The theoretical Nd moment is calculated by μ_sat_ = *g*_J_ *J*μ_B_, where *g*_J_ is given in SI eq S6 and *J* is the total angular momentum number; this yields values of 3.27 μ_B_ and 3.20 μ_B_ for Nd in the 3+ and 4+ oxidation states, respectively. The highly suppressed refined Nd moment relative to the theoretical value is consistent with the small entropy release on heating through the ordering temperature which was observed by magnetic heat capacity.

The Mn–Mn–Mn trimers likely behave as independent magnetic clusters with an effective spin $S=\frac{3}{2}$ , which was reported for the Ce- and Pr-containing materials.^10^ This is likely a primary driver of the magnetic structure that we observe in this material.

Within the structure, there are a total of five magnetic interactions that are likely to contribute to magnetic ordering. These are all shown in Figure 9.

**Figure 9:**
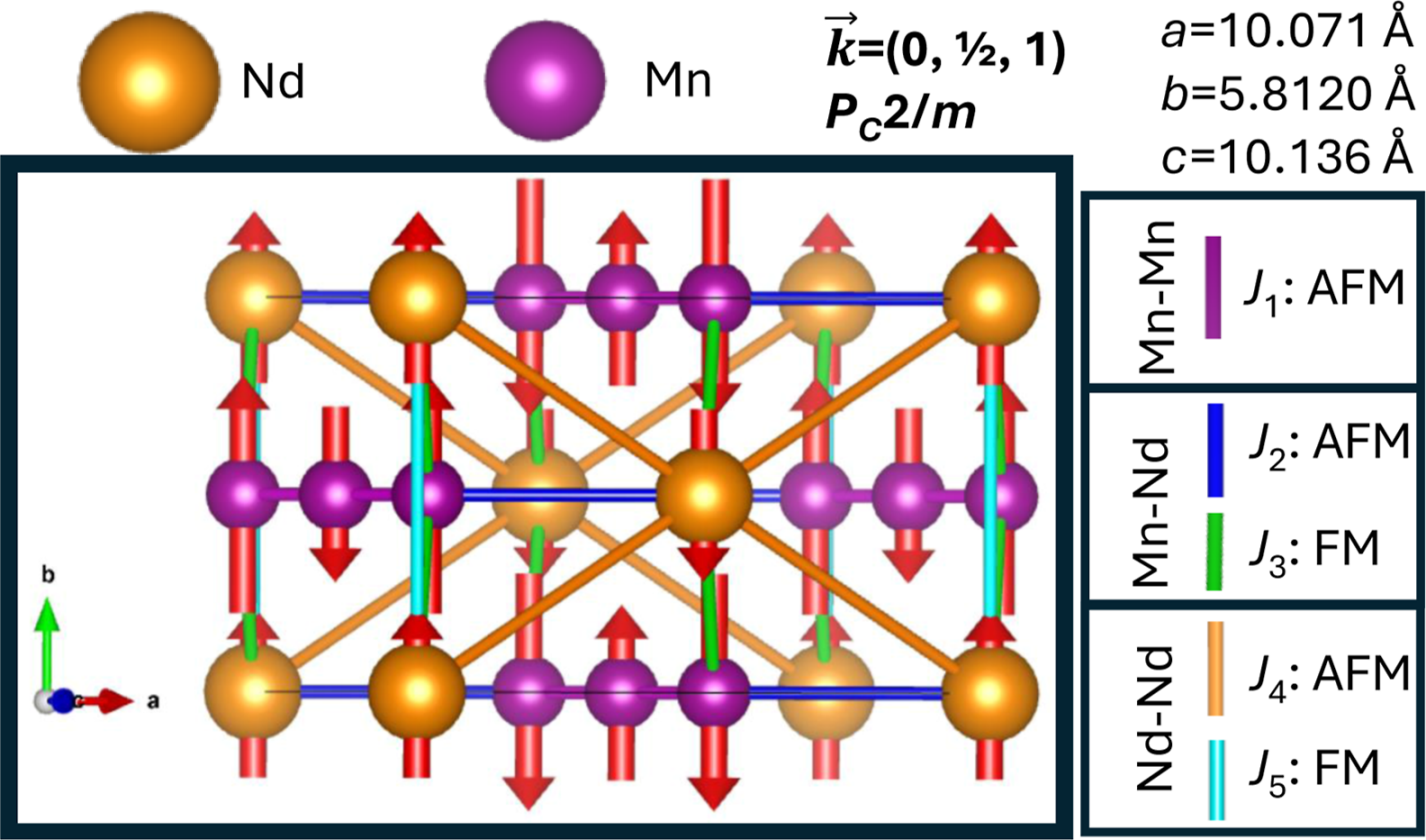
Magnetic structure of 12R-BNM at 8 K, obtained using a *k⃗* = (0, ^1^/_2_, 1) propagation vector and a *Pc*2/*m* (no. 10.49) magnetic space group, with O vacancy concentration fixed to the value obtained by Rietveld refinement of the 200 K data set. *J*_1_ refers to the intratrimer Mn–Mn interactions (purple); *J*_2_ refers to the antiferromagnetic Nd–Mn interactions (dark blue); *J*_3_ refers to the ferromagnetic Nd–Mn interactions (green); *J*_4_ refers to the antiferromagnetic Nd–Nd interactions (orange); and *J*_5_ refers to the ferromagnetic Nd–Nd interactions parallel to the *b*-axis (light blue). Ba cations and O anions are excluded.

*J*_1_ is the intratrimer Mn–Mn interaction. As has been the case in other structures with this Mn trimer structural motif, which were studied with magnetic neutron diffraction, such as the Pr- and Ce-containing analogs of this structure^10^ and the Ln-free Ba_4_Mn_3_O_10_ material,^13^ this interaction is antiferromagnetic. This result can be understood in terms of the direct exchange interaction, which favors antiparallel alignment when there is direct overlap of half-filled t_2g_ orbitals.

The *J*_2_ and *J*_3_ interactions are approximately the same distance, but they lead to different correlations. A single Nd cation will have two *J*_2_ interactions but four *J*_3_ interactions. The *J*_2_ interaction, which occurs within the *ac*-plane, is antiferromagnetic, whereas the *J*_3_ interaction is ferromagnetic. Given that the antiferromagnetic interactions of Mn trimer spin clusters means that all Nd–Mn interactions can be of the same type, the dominance of the ferromagnetic *J*_3_ interaction suggests it is lower in energy than the antiferromagnetic interaction. A simple explanation may be that the Nd 4f orbitals are polarized by the effective moment of the Mn–Mn–Mn trimers via a minimization of magnetic free energy, given by $E=-\vec{\mu_{Nd}\;}\cdot \mathrm{B⃗}$ where *B⃗* is magnetic field due to the trimer clusters. An alternative explanation is that there is a direct exchange coupling between the Mn^4+^ 3d orbitals and the Nd^4+^ 4f orbitals, although this seems less likely due to the localized nature of 4f orbitals.

Finally, we note that the Nd–Nd interactions *J*_4_ (four per Nd) and *J*_5_ (two per Nd) are antiferromagnetic and ferromagnetic, respectively, although at quite a long distance (>6 Å). While it is possible that there may be some exchange between Nd 4f orbitals, it is more likely that the nature of these interactions are determined by the Mn–Mn and Mn–Nd interactions, *J*_1_ to *J*_3_, than being of fundamental importance in determining the magnetic structure of this material.

This magnetic structure has no features (such as ferrimagnetism or canting) that could explain the hysteresis observed in Figure 5. This is similar to the magnetic structure of Ba_4_Mn_3_O_10_, which also has a collinear magnetic structure despite a small hysteresis in variable-field magnetometry measurements at low temperature. It is possible that there is a low-intensity additional magnetic component to the structure which cannot be resolved in these data, corresponding to a ferro/ferrimagnetic phase. However, the hysteresis does appear to persist above the magnetic ordering temperature, suggesting that it may be a local phenomenon. A future muon-spectroscopy study may be needed to test these hypotheses.

## Discussion

A significant result of this work is the partial stabilization of Nd^4+^. In oxides, we can find only one prior work that claims partial site occupancy with Nd^4+^, allegedly accomplished via the partial substitution of Nd on the *B*-site of perovskites by Brauer and Kristen (1979).^99^ However, in this work there is no spectroscopic or magnetometry evidence for the presence of Nd^4+^. The possibility of Nd reduction to the 3+ oxidation state, compensated by O vacancies, appears entirely consistent with their findings. In contrast, while our material certainly exhibits partial Nd reduction to the 3+ oxidation state, we have two direct pieces of evidence for the presence of Nd^4+^ in our material. First, the XAS data at the Nd L_3_-(Figure 2b) and L_2_-(Figure S23) edges indicate that Nd is either trivalent or, more likely, mixed tri- and tetra-valent. Second, the nonlinearity of inverse magnetic susceptibility with curvature $\frac{d^{2}\chi \left(T\right)}{dT^{2}}<0$ at high temperatures (Figure S36) is a sign of Van Vleck paramagnetism, from which Nd^4+^ would be the only likely source. We therefore believe this study is the first to demonstrate the presence of Nd^4+^ in a metal oxide.

The magnetic structures of the Ce- and Pr-containing materials are very similar, but for the fact that replacing nonmagnetic Ce^4+^ with magnetic Pr^4+^ results in a spin canting within Mn_3_O_12_ trimers. Given this, it could be expected that substituting Nd on the Ln site might logically result in a similar magnetic structure with enhanced spin canting angle. It is therefore surprising that the magnetic structure we observe does not follow this trend, showing no spin canting at all and an entirely different propagation vector and magnetic symmetry. This may in part be due to the presence of O vacancies, which would disrupt magnetic superexchange significantly. However, there are other features observed from the magnetic properties of Nd in 12R-BNM, such as its low saturation relative to its theoretical moment as seen from Rietveld refinement, and its almost paramagnetic coupling to external magnetic fields in the ordered regime as observed by the rapid suppression of a λ transition with application of field. These differences likely result from the enhanced moment in Nd^4+^/Nd^3+^ relative to Pr^4+^, and could also explain the significantly different magnetic structure compared with the Ce- and Pr-containing analogs.

There remains much work to be done to understand the magnetic properties of this compositional series. For 12R-BNM, magnetic pair distribution function^100–102^ may show how Nd locally correlates with Mn–Mn–Mn trimers and how this differs from the Pr-containing material. There would also be value in performing neutron diffraction to much lower temperatures, such as the mK regime, to see if Nd saturation eventually occurs and results in a Mn^4+^ spin canting like the Pr-containing material. Beyond the Nd-containing compound, the previously reported Nb-containing,^14,24^ Ta-containing,^17^ and Sb-containing^16,17^ materials, 12R-Ba_4_ *M*Mn_3_O_12_ (*M* = Nb, Sb), have been studied using magnetometry but not yet magnetic neutron diffraction, which would help us understand the range of magnetic structures which can be adopted by this class of material. Variants with significant oxygen-deficiency with *M* = Y, In have also been reported,^31,32^ with an antiferromagnetic transition in the Y-containing analog, but the magnetic structure of these materials remain undetermined. Finally, synthesis efforts should be directed at obtaining 12R-Ba_4_LnMn_3_O_12_ materials containing Ln cations with larger moments, if possible, to see what effect this has on the intratrimer magnetic ordering and interactions between the magnetic clusters.

## Conclusion

By synthesizing and studying the hexagonal perovskite 12R-Ba_4_NdMn_3_O_12−γ_, we have demonstrated the first stabilization of Nd^4+^ in a metal oxide, with partial reduction to Nd^3+^ driven by oxygen vacancy formation.

We find that the Nd cation behaves differently than cations with a lower f-electron count, such as Ce and Pr, substituted on the same site.^9,10^ This manifests as a weaker participation in long-range magnetic ordering and a stronger coupling to external magnetic fields. These effects, potentially coupled with suppressed superexchange due to O vacancies, manifest an entirely new magnetic structure below *T*_N_ ≈ 16 K not yet observed in hexagonal perovskites with 12R stacking. We also find trends with composition, such as increasing *T*_N_ and decreasing magnetic frustration in the series from Ln = Ce–Nd.

Additionally, we identify a previously unrecognized off-centering of the edge Mn cations within the Mn–Mn–Mn trimers, which, by comparison with Ce- and Pr-containing analogs,^9^ emerges as a general structural motif of the 12R polytype. This behavior is consistent with a second-order Jahn–Teller distortion, providing new insight into the latent vibronic instability of Mn^4+^ and its role in shaping the local structure of correlated oxides.^33,44,45^

These findings establish 12R-stacked hexagonal perovskites as a tunable platform, where variations in lanthanoid spin, oxygen stoichiometry, and local structure give rise to diverse magnetic ground states beyond conventional compositional trends. This therefore offers a promising route to systematically probe and control magnetic ground-state selection in correlated oxides.

## Methods

### Materials

All chemicals were purchased and used as-received: Barium nitrate (Ba­(NO_3_)_2_, Alfa Aesar ACS, 99+%), neodymium­(III) nitrate hexahydrate (Nd­(NO_3_)_3_·6H_2_O, Alfa Aesar, 99.9%), manganese­(II) nitrate tetrahydrate (Mn­(NO_3_)_2_·4H_2_O, Alfa Aesar, 98%), and anhydrous citric acid (Fisher Scientific, certified ACS).

### Synthesis

For a 3 g scale reaction, barium nitrate (2.9857 g), neodymium­(III) nitrate hexahydrate (1.2519 g), manganese­(II) nitrate tetrahydrate (2.1507 g), and anhydrous citric acid (3.2923 g) were suspended in ∼75 mL of deionized water. On a hot plate with stirring, most of the water was evaporated until a viscous liquid was obtained. This was then dried in an oven set at 110 °C overnight. The resulting solids were ground into powder then self-combusted on a hot plate. From this, two samples were prepared. The first, which hereafter we refer to as 12R-BNM-S1, was then ground, calcined at 800 °C (5 °C/min) in air for 12 h, then sintered at 1350 °C (10 °C/min) for another 24 h with intermediate grinding. The second sample followed a similar synthesis route, except the 1350 °C sintering stage was performed at 800 °C, followed later by an additional annealing step in air at 1300 °C for 12 h without intermediate grinding. This sample we refer to as 12R-BNM-S2. Both samples exhibit identical magnetic and structural properties, to the extent studied in this work. All data presented in this manuscript are measured on 12R-BNM-S2, with data on 12R-BNM-S1 presented in the Supporting Information to show the repeatability of the structural and magnetic properties, and the invariance of properties with synthesis route.

We note, as shown in Figure 1c, that a single-phase sample was not obtained; for 12R-BNM-S2, a substantial impurity (∼11.8 wt %) of Ba_4_Mn_3_O_10_ is observed, here refined using the orthorhombic *Cmca* structure reported by Zubkov et al.^13^ A similar impurity fraction was obtained for 12R-BNM-S1 (Figure S6). As will be shown, care is taken to separate out the contributions of this impurity phase from the magnetic properties intrinsic to 12R-BNM.

### Synchrotron X-ray Diffraction

An aliquot of 12R-BNM was placed in a 0.5 mm diameter quartz capillary and measured on BL2-1 at SSRL^103^ using an X-ray beam of energy 17 keV. Wavelength was refined using a LaB_6_ standard.

Low-temperature synchrotron XRD measurements between 80 and 300 K were performed on the same beamline, using a cryostream capillary cooling system. Data were measured on a silica standard in addition to the 12R-BNM sample in order to calibrate temperature, using thermal expansion coefficients taken from Pein et al.^104^ and discussed in detail in the Supporting Information.

### Magnetometry

DC and AC susceptibility data were collected on both a commercial Quantum Design Physical Properties Measurement System (PPMS) and a Quantum Design Magnetic Properties Measurement System 3 (MPMS3) magnetometer. For PPMS measurements (12R-BNM-S1 variable field measurements and all 12R-BNM-S2 measurements), an aliquot of the sample was loaded into a plastic straw. For MPMS measurements (12R-BNM-S1 variable-temperature measurements), an aliquot of the sample was loaded into an encapsulated nonmagnetic plastic container and held in a brass sample holder for variable-temperature measurements. For variable-temperature DC susceptibility, measurements were taken during warming after cooling in zero field (ZFC-W) and in a high field (FC-W), where multiple measurements were performed with different external fields. Variable-field DC magnetization measurements were assumed to be performed under an isothermal condition in a magnetic field stabilized at each set point between ±140 kOe or ±70 kOe depending on the temperature. AC susceptibility was measured in the PPMS with a 1.5 Oe drive current at frequencies greater than 1500 Hz and with a 5 Oe drive current at frequencies lower than 1500 Hz.

### Heat Capacity

Heat capacity was measured using a commercial Quantum Design PPMS, with sample pressed into pellets of powder and subjected to applied fields up to μ_0_ *H* = 14 T. Apiezon N grease was used to adhere the pellet to the puck. Three data points were collected at each temperature; the value used was the average of the second and third data points.

The heat capacity of the addenda was measured separately and subtracted from the total heat capacity. As shown in Figure S39, the Debye equation^105^ could not be used to model the lattice component of the entropy, so all nonmagnetic contributions, *C*_bkg_, to the heat capacity were subtracted using the following empirical relation

3 $$C_{bkg}=aT^{3}+bT^{2}$$

where *T* is temperature and *a* and *b* are empirical parameters. This same empirical relation has been used previously.^9^ Fitting was performed using scipy^106^ in the temperature range 20 to 40 K, on data measured under an applied field of 14 T. Resulting data, *C*_mag_ = *C*_total_ – *C*_bkg_, are assumed to be entirely magnetic in origin.

### X-ray Absorption Spectroscopy

XAS was measured at Stanford Synchrotron Radiation Lightsource. For the hard XAS (Nd L-edges, Mn K-edge, and Ba L-edge), measurements were performed on Beamline 2–2 ^107^ in transmission mode using an Si(111) monochromator (ϕ = 90° from the beam axis), with sample prepared in a metal grid held in place using kapton tape. For soft XAS (Mn L-edge, Nd M_5_-edge, and O K-edge), total electron yield and total fluorescence yield measurements were performed in vacuo on Beamline 8–2 ^108,109^ For all measurements, reference data were also taken and later used for alignment (by matching the maximum in the first derivative, corresponding to the first inflection point, of the reference data). Repeated scans were taken and averaged to improve signal quality. Athena^110,111^ was used for data normalization and averaging. We note that the Mn L-edge data for 12R-BCM and 12R-BPM, reproduced from Regier et al.,^10^ was measured on beamline 10–1 but compared to 12R-BNM data measured on beamline 8–2.

For the Nd L_1_-, L_2_-, and L_3_-edges of 12R-BNM, Nd_2_O_3_ reference data were measured using an Nd_2_O_3_ standard with diffraction pattern shown in Figure S33.

### Neutron Diffraction

PND was measured both on the WAND^2^ constant-wavelength neutron diffractometer^112^ at the High Flux Isotope Reactor, and on frame 3 of the POWGEN time-of-flight powder diffractometer^94^ at the Spallation Neutron Source, both at Oak Ridge National Laboratory. Samples were cooled in WAND^2^ data using an Orange cryostat with belt sample changer. For the constant-wavelength neutron diffraction data at WAND^2^, the wavelength was obtained as 1.48642(5) Å via refinement against a NIST Si standard. For the POWGEN experiment, approximately 2 g of powder was loaded into a 6 mm diameter vanadium can and placed in the POWGEN automatic sample changer. Data were collected at 8, 20, 60, and 200 K using central wavelength of λ = 1.5 Å (covering a *d*-spacing range of 0.8–11.8 Å) for 1.5 hrs at each temperature.

### Diffraction Analysis

Diffraction data were analyzed in topas 7 ^113^ using Rietveld refinement^26^ assuming a *R*3̅*m* space group the same as 12R-BCM and 12R-BPM as reported in refs 9 and 10 . In these Rietveld refinements, background due to instrumental contributions/diffuse scattering was modeled using a Chebyshev polynomial. Diffraction peakshape was modeled using the Thompson-Cox-Hastings approach.^114^ Anisotropic peak broadening was modeled using the Stephens’ approach.^115^ For Rietveld refinement of magnetic neutron scattering, the form factors used are given in Table S7.

For corefinement of synchrotron X-ray (beamline 2–1, SSRL) and neutron (POWGEN) diffraction data, a relative weighting scheme was established such that both data sets contributed equally to the refinement despite the inequivalent signal-to-noise. All parameters were linked equally except background parameters, peak shapes, scale factors, and lattice parameters, with lattice parameters refined separately to account for tiny differences in temperature.

Bond valence sum calculation used bond lengths obtained by Rietveld refinement to obtain a proxy for valence, *V*, using the following equation

4 $$V=\sum_{i=1}^{6}\exp\left[\frac{r_{0}-r_{i}}{B}\right]$$

where *r*_*i*_ are the *M*–O bond lengths (*M* = Nd, Mn), the empirical parameter *r*_0_ is 1.750 Å for Mn^4+^ and 2.086 Å for Nd, and *B* = 0.37 Å (taken from ref 116).

### Thermogravimetric Analysis

Netzsch STA 449 F1 Jupiter thermal analyzer was used for performing thermogravimetric analysis. Baseline correction was performed with an empty crucible with no sample. Experiments were conducted under gas flow rates of 100 mL/min. Argon gas (Matheson, UHP grade) and air (Matheson, ultrazero grade) were used as received.

### Scanning Electron Microscopy

SEM was conducted at 10 keV accelerating voltage on a ThermoFisher Phenom XL Desktop SEM. To prepare the samples for measurement, powder was deposited on carbon tape.

### First-Principles Density Functional Theory (DFT) Calculations

DFT total energy calculations were performed using the projector augmented wave (PAW) method^117,118^ as implemented in the VASP (Vienna Ab-initio Simulation Package) code.^119,120^ The generalized gradient approximation of Perdew et al.^121^ was used for DFT exchange and correlation along with a Hubbard U correction term^122^ with parameter U–J of 3 eV for Mn-d and 1.5 eV for Nd-d and 2 eV for Nd-f orbitals. The PAW potentials “Ba_sv”, “O_s”, “Mn,” and “Nd”, distributed with VASP were used with a plane wave energy cutoff of 380 eV, consistent with the computational approach employed in previous DFT studies on transition metal oxides.^123–125^ Note that the U values used here are motivated by thermochemical considerations,^124,126^ and do not intend to correct for the well-known DFT band gap problem, for which DFT + U alone is not suitable. We performed DFT + U calculations on a 12R phase (space group *R*3̅*m*, no. 166) of 12R-BNM. The calculations were based on a 40-atom cell with the previously determined lowest-energy collinear AFM configurations for Mn in 12R-BCM^127^ and Pr in 12R-BPM,^10^ and we used a Γ-centered 2 × 4 × 2 *k*-mesh for DOS calculations. The local magnetizations and orbital-resolved partial DOS were determined with integration radii of 0.66, 1.50, 2.15, and 2.01 Å for O, Mn, Ba, and Nd, respectively, taken as the covalent radii of ref 128. A Gaussian smoothing with σ = 0.05 and 0.20 eV was applied to the DOS shown in the vicinity of the Fermi level and up to 50 eV above the conduction band maximum, respectively.

## Supplementary Material



## Data Availability

The crystal structure of 12R-Ba_4_NdMn_3_O_12_ is available at CDC 2562296.

## Footnotes

1 Note that these DFT + U values are not expected to accurately predict the true band gaps, but they should correctly reflect the chemical trend.

## Notes

SEM; structural and magnetic characterization; thermogravimetric analysis; low-temperature synchrotron X-ray diffraction; Rietveld refinements of neutron diffraction data; XAS; X-ray diffraction of the Nd_2_O_3_ standard; Curie–Weiss fitting; heat-capacity analysis; theoretical magnetic entropy calculation; magnetic form factor calculation; and DFT details (PDF)
