# Supplementary material for: Mixed Nd4+/3+ and Cluster Magnetism in Hexagonal Perovskite 12R-Ba4NdMn3O12−γ
Source: J Am Chem Soc. 2026 Jul 24;148(30):32396–411. doi: 10.1021/jacs.6c08795 (PMC13450426; doi:10.1021/jacs.6c08795)
Supplement: Supplementary file 1 [file ja6c08795_si_001.pdf]

Mixed $Nd^{4+/3+}$ and cluster magnetism in hexagonal perovskite 12R-$Ba_4NdMn_3O_{12-\gamma}$

Liam A. V. Nagle-Cocco,[*,†] Madeline G. Port,[†,‡] James Eujin Park,[¶] Dennis Nordlund,[†] Oliver Mueller,[†] Stephan Lany,[§] Anna A. Berseneva,[§] Alicia M. Manjón-Sanz,[‖] Danielle R. Yahne,[‖] Mathias A. Kiefer,[†] Yuntian Li,[⊥] Benito Ariceaga Gonzalez,[⊥] Eric N. Coker,[¶] Andrea Ambrosini,[¶] Ryan A. Klein,[#] Rebecca W. Smaha,[*,§] and Nicholas A. Strange[*,†]

†*Stanford Synchrotron Radiation Lightsource, SLAC National Accelerator Laboratory, Menlo Park, California 94025, United States of America*

‡*Department of Materials Science and Engineering & Department of Chemistry, University of Pennsylvania, Philadelphia, Pennsylvania 19104, United States of America*

¶*Sandia National Laboratories, Albuquerque, New Mexico 87185, United States of America*

§*Materials, Chemical, and Computational Sciences Directorate, National Laboratory of the Rockies, Golden, Colorado 80401, United States of America*

‖*Neutron Scattering Division, Oak Ridge National Laboratory, Oak Ridge, Tennessee 37831, United States of America*

⊥*Geballe Laboratory for Advanced Materials and Department of Applied Physics, Stanford University, California 94305, United States of America*

#*Department of Chemistry, University of California, Berkeley, California 94720, United States of America*

E-mail: lnc@slac.stanford.edu; rebecca.smaha@nlr.gov; nstrange@slac.stanford.edu

Supplementary Information

Contents

	List of Tables	3
	List of Figures	3
S1	Scanning Electron Microscopy	6
S2	Testing Nd reactivity with alumina crucible	9
S3	Structural and magnetic characterisation of 12R-BNM-S1	13
S4	Thermogravimetric analysis	19
S5	Low-temperature synchrotron X-ray diffraction	22
S6	Rietveld refinements of neutron diffraction data	24
S7	X-ray absorption spectroscopy	35
S8	X-ray diffraction on neodymium (III) oxide standard	43
S9	Curie-Weiss fitting to magnetometry data	44
S10	Expected magnetic moment in the paramagnetic regime	50
S11	Heat capacity analysis details	53
S12	Calculation of theoretical maximum magnetic entropy change during ordering	57
S13	Magnetic form factor calculation	59
S14	Density Functional Theory	61
	References	63

List of Tables

S1 Thermal expansion coefficients for SiO_2 used for temperature calibration . . 22

S2 Oxygen vacancy content from Rietveld refinement of POWGEN neutron diffraction data on 12R-BNM-S2 25

S3 Refined crystallographic parameters from 200 K neutron diffraction data on 12R-BNM-S2 26

S4 Magnetic structure from neutron diffraction at 8 K 28

S5 Expected effective magnetic moments for $Ba_4LnMn_3O_{12}$ compounds 51

S6 Comparison of calculated magnetic moments for 12R-$Ba_4NdMn_3O_{12}$ under different assumptions 52

S7 Empirical parameters for magnetic form factor calculations 59

S8 DFT-calculated oxygen vacancy formation energies for 12R-$Ba_4LnMn_3O_{12}$ (Ln = Ce, Pr, Nd) 61

List of Figures

S1 SEM images for 12R-BNM and 12R-BCM 7

S2 SEM/EDX images for 12R-BNM and 12R-BCM 8

S3 Alumina substrate following annealing of Nd-containing materials 9

S4 Grazing-incidence X-ray diffraction of alumina substrate following annealing of Nd_2O_3 11

S5 Grazing-incidence X-ray diffraction of alumina substrate following annealing of 12R-BNM 12

S6 Rietveld refinement using synchrotron XRD of 12R-BNM-S1 13

S7 DC magnetic susceptibility of 12R-BNM-S1 14

S8 Inverse DC magnetic susceptibility of 12R-BNM-S1 16

S9 DC magnetic susceptibility of 12R-BNM-S1 17

S10 AC susceptibility of 12R-BNM-S1 17
S11 Comparison of magnetic heat capacity and entropy for 12R-BNM-S1 and 12R-BNM-S2 18
S12 Powder neutron diffraction data on 12R-BNM-S1 collected on the WAND^2 diffractometer 18
S13 Thermogravimetric analysis on 12R-BNM for water-splitting screening 20
S14 Thermogravimetric analysis on 12R-BNM during reduction 21
S15 Temperature-dependence of lattice parameters in 12R-BNM-S2 23
S16 Neutron diffraction data from POWGEN at 200 K with various O vacancy scenarios 29
S17 Combined Rietveld refinement of synchrotron and neutron diffraction assuming a single O vacancy parameter 30
S18 Combined Rietveld refinement of synchrotron and neutron diffraction assuming two O vacancy parameters 31
S19 Neutron diffraction data from POWGEN at 60 K 32
S20 Neutron diffraction data from POWGEN at 20 K 33
S21 Neutron diffraction data on 12R-BNM-S2 at 8 K with fixed O vacancy concentration 34
S22 Normalized XAS spectra at the Nd M-edge for BNM 36
S23 Normalized XAS spectra at the Nd L-edge for BNM 36
S24 Normalized XAS spectra at the Ba L-edge for BNM and BCM 37
S25 Normalized XAS spectra at the Mn L-edge without area-normalisation . . . 37
S26 Normalized raw and merged XAS spectra at the Mn L-edge for BNM 38
S27 Normalized raw and merged XAS spectra at the Mn K-edge for BNM and BCM 39
S28 Normalized XAS spectra at the Mn K-edge without area-normalisation . . . 39
S29 Normalized raw and merged XAS spectra at the O K-edge for BNM 40
S30 Normalized raw and merged XAS spectra at the Nd L-edges for BNM 40

S31 Normalized raw and merged XAS spectra at the Nd M-edges for BNM . . . 41
S32 Normalized raw and merged XAS spectra at the Ba L-edges for BNM and BCM 42
S33 Diffraction data and Rietveld refinement on Nd_2O_3 standard 43
S34 Inverse DC magnetic susceptibility for 12R-BNM-S2 45
S35 Curie-Weiss fit parameters as a function of T_{min} for 12R-BNM-S2 47
S36 Curie-Weiss fit to inverse susceptibility data for 12R-BNM-S2 48
S37 Comparison of high-temperature linear fits with and without $Ba_4Mn_3O_{10}$ impurity correction 49
S38 Experimentally-measured heat capacity datasets on 12R-BNM-S2 53
S39 Evidence that lattice heat capacity is not Debye-like 54
S40 Heat capacity fits for BNM 55
S41 Heat capacity data with background subtracted 56
S42 Oxygen vacancy formation energy from DFT+U calculations 61
S43 Density-of-States for stoichiometric 12R-$Ba_4Mn_3\mathit{Ln}O_{12}$ (Ln=Pr,Nd) 62

S1 Scanning Electron Microscopy

Scanning electron microscopy (SEM) was conducted at 10 keV accelerating voltage on a ThermoFisher Phenom XL Desktop SEM. To prepare the samples for measurement, powder was deposited on carbon tape. Figure S1 compares the sample morphology of 12R-BNM and 12R-BCM. The average particle size for 12R-BNM is far smaller than that of 12R-BCM, though not sufficently small to manifest in diffraction data *via* Scherrer broadening, and so we do not believe particle size impacts the results discussed elsewhere in this work.

Additionally, electron dispersive spectroscopy was conducted on the Thermo Fisher Phenom XL Desktop SEM using a 15 keV accelerator voltage. This is shown in Figure S2.

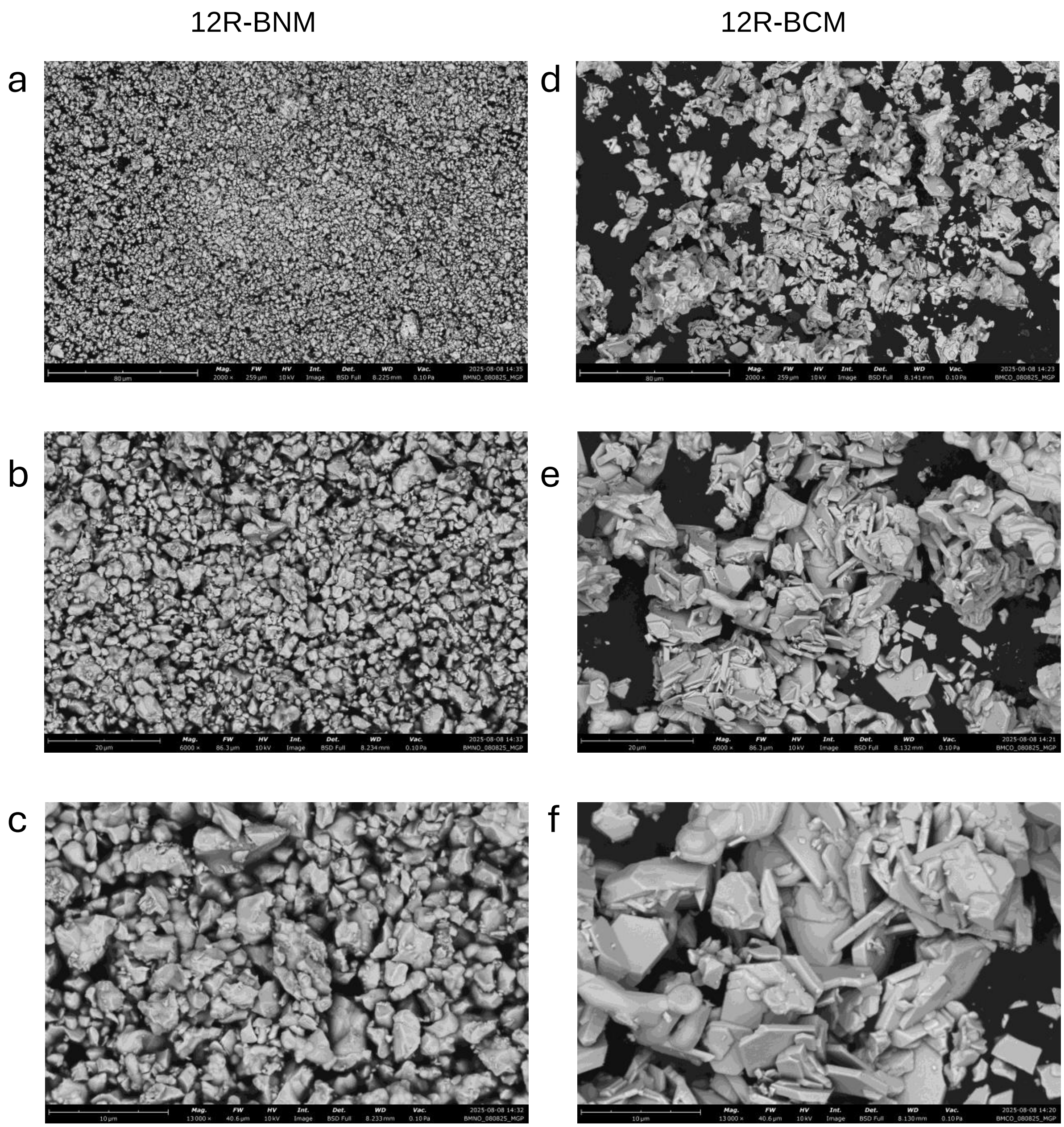

Figure S1: SEM images for 12R-BNM (a-c) and 12R-BCM (d-f). Figures are at (a,d) 2000x, (b,e) 6000x, and (c,f) 13000x magnification. The scale bars within the panels are (a,d) 80 micron, (b,e) 20 micron, and (c,f) 10 micron.

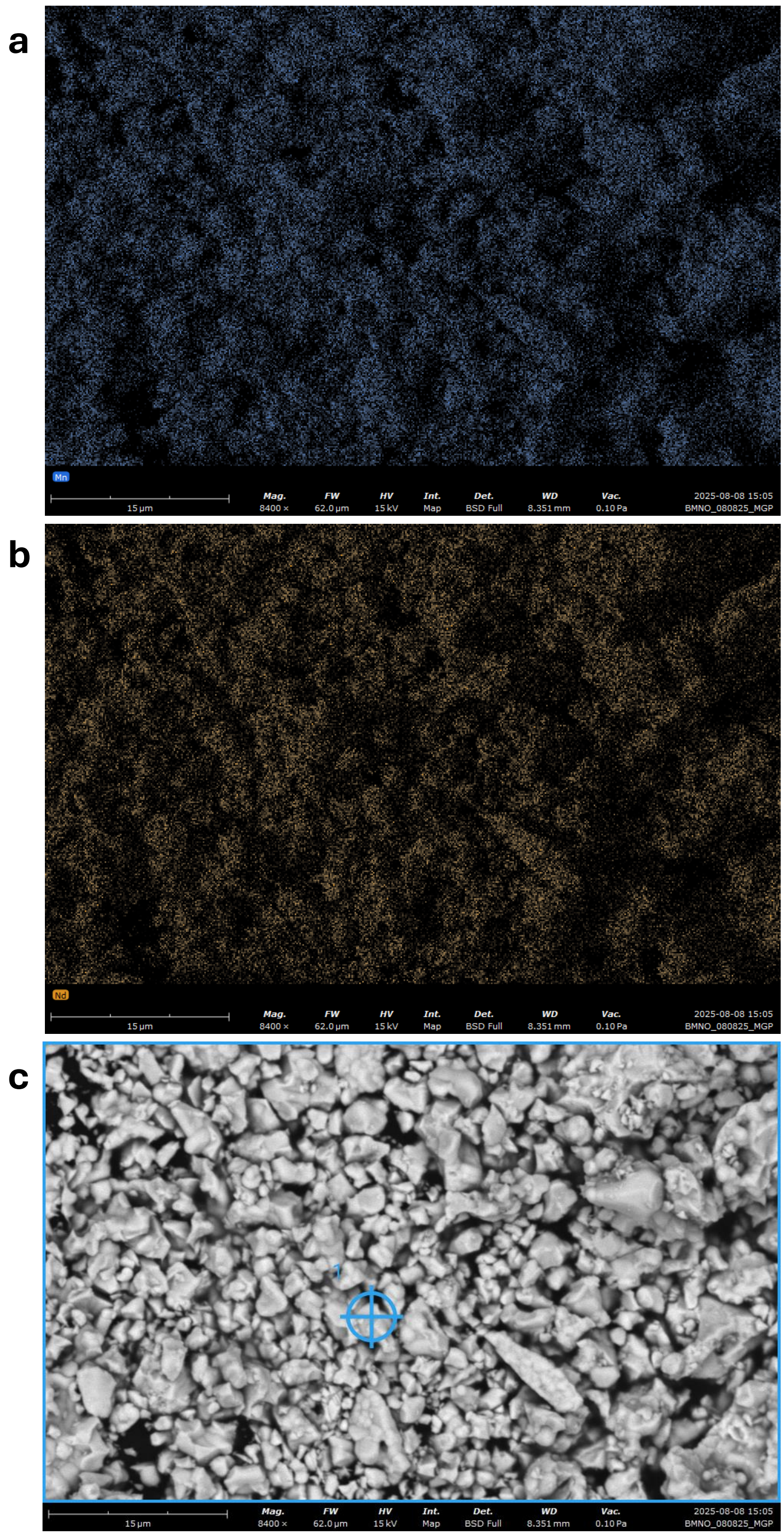

Figure S2: Electron-dispersive X-ray spectroscopy data for (a) Mn and (b) Nd, for a region imaged via SEM in (c).

S2 Testing Nd reactivity with alumina crucible

As noted in the main text of the manuscript, and shown in Figures 1(c) and S6, there is the formation of a $Ba_4Mn_3O_{10}$ impurity phase during synthesis of 12R-BNM. This might indicate loss of Nd during the reaction. We note that the work on the Ce- and Pr-containing analogs[1,2] also observed formation of this impurity phase, albeit in smaller quantities, suggesting that lanthanide loss during synthesis may be common amongst the compositional series. While lanthanides such as Nd are known to volatise at high-temperatures,[3,4] suggesting sublimation of Nd as a possible driver of Nd loss and consequent $Ba_4Mn_3O_{10}$ formation, we also note some evidence for reaction with alumina.[5,6] To test this, we scooped an aliquot of each of powdered, non-pelletised Nd_2O_3 and 12R-BNM onto two ends of an alumina substrate (MSE supplies; claimed purity > 99%) and annealed in air at high-temperatures (1320°C for 14 hrs) comparable to the synthesis of 12R-BNM. While this led to no colour change for Nd_2O_3, we saw a clear colour change for the 12R-BNM heating, as shown in Figure S3.

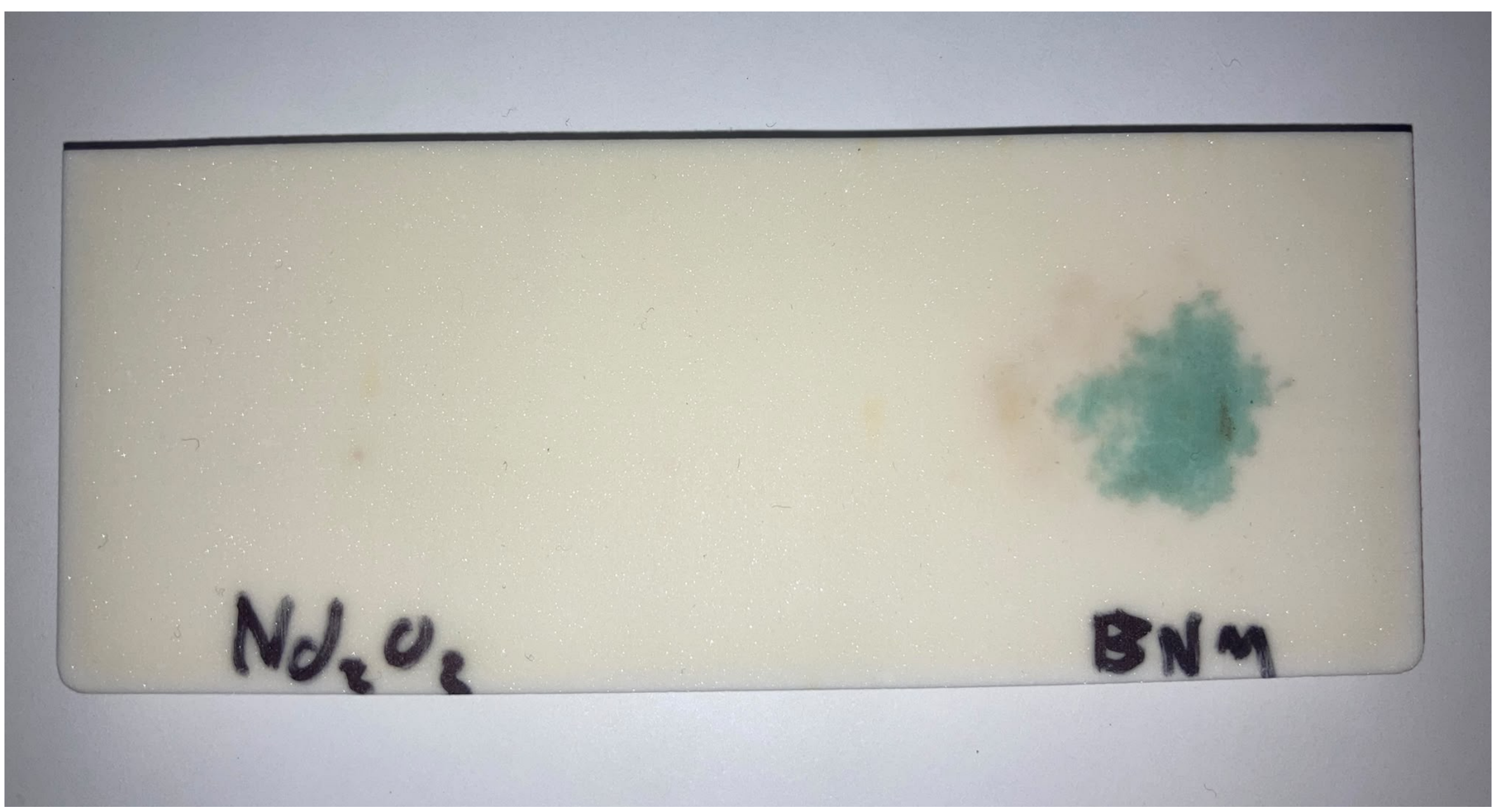

Figure S3: Photograph of the alumina substrate (MSE supplies; claimed purity > 99%) upon which powdered, non-pelletised Nd_2O_3 (left) and 12R-BNM (right) were annealed at 1320°C for 14 hrs in air, then removed, leaving a colour change for the 12R-BNM annealing. The crucible was then subjected to grazing-incidence X-ray diffraction (with incident angle of 5° and energy 17 keV). Small yellow spots are due to X-ray beam damage.

To investigate this further, we performed grazing-incidence X-ray diffraction on both sides of the alumina substrate using beamline 2-1 at Stanford Synchrotron Radiation Lightsource. This experiment used a fixed incident angle of 5° and X-ray beam energy 17 keV. We find clear evidence of reaction with the alumina substrate in both cases. We note that Rietveld refinement is not possible for this data due to the unknown extent of polycrystallinity and hence unconstrained ratios of peak intensities; preliminary investigation used Pawley analysis[7] but ultimately fitting is presented here as phase identification is not definitive. We note we were not able to match all the Bragg peaks that appeared from the reaction for either of the heated powders. One reason for this may be that Mg-containing impurities in the alumina crucible might also react and produce phases, but due to the vast phase space of possible materials and the non-criticality of comprehensive phase identification for testing our hypothesis of sample-alumina reaction, we did not explore Mg-containing impurities.

In the case of Nd_2O_3@alumina, we see potential signatures of $Nd_4Al_2O_9$ (ICSD 249018; space group $P2_1/c$) and $NdAl_{11}O_{18}$ ($P6_3/mmc$[8]), along with NdO(OH) (space group $P2_1/m$). The former two likely formed due to high-temperature reaction of Nd_2O_3 with the alumina; the latter phase would likely have formed at room-temperature after the annealing but before the X-ray diffraction measurement due to reaction of surface Nd-containing phases with moisture. The present phase identification is sufficient for determining the plausibility of Nd loss to the crucible. This data is shown in Figure S4.

In the case of 12R-BNM@alumina, we see clear evidence of reaction between 12R-BNM and the alumina substrate during annealing, identifying two likely reaction products alongside the substrate: $Nd_4Al_2O_9$ (space group $P2_1/c$) and $BaAl_{1.88}Mn_{0.12}O_4$ (space group $P6_3$). Both phases require alumina as a reactant, and both require constituents of 12R-BNM — Nd in the former case, Ba and Mn in the latter — indicating that the 12R-BNM reacts with the crucible at such temperatures. This data is shown in Figure S5.

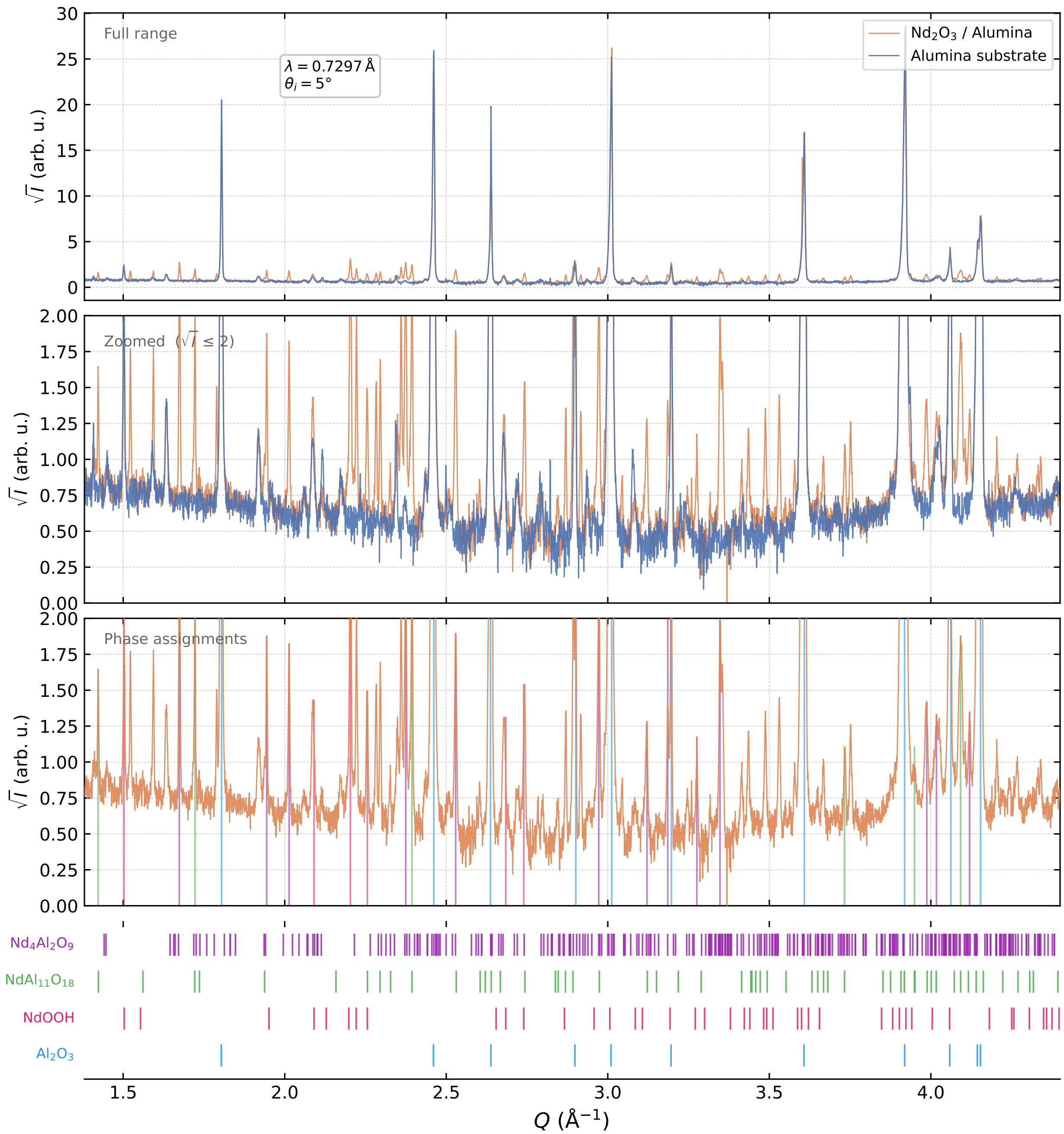

Figure S4: Grazing-incidence X-ray diffraction patterns (incident angle $\theta_i = 5°$, energy 17 keV, $\lambda = 0.7297$ Å) of the bare alumina substrate (MSE Supplies; claimed purity > 99%) and the same substrate following annealing of powdered, non-pelletised Nd_2O_3 at 1320°C for 14 hrs in air. The upper panel shows the full intensity range; the middle panel shows the same data with the intensity axis capped at $\sqrt{I} \leq 2$ (arb. u.) to reveal weaker reflections. The lower panel shows predicted peak positions for the four phases identified: Al_2O_3 (corundum), NdOOH, $NdAl_{11}O_{18}$, and $Nd_4Al_2O_9$, indicating reaction between the Nd_2O_3 powder and the alumina substrate during annealing. There remain numerous unassigned peaks.

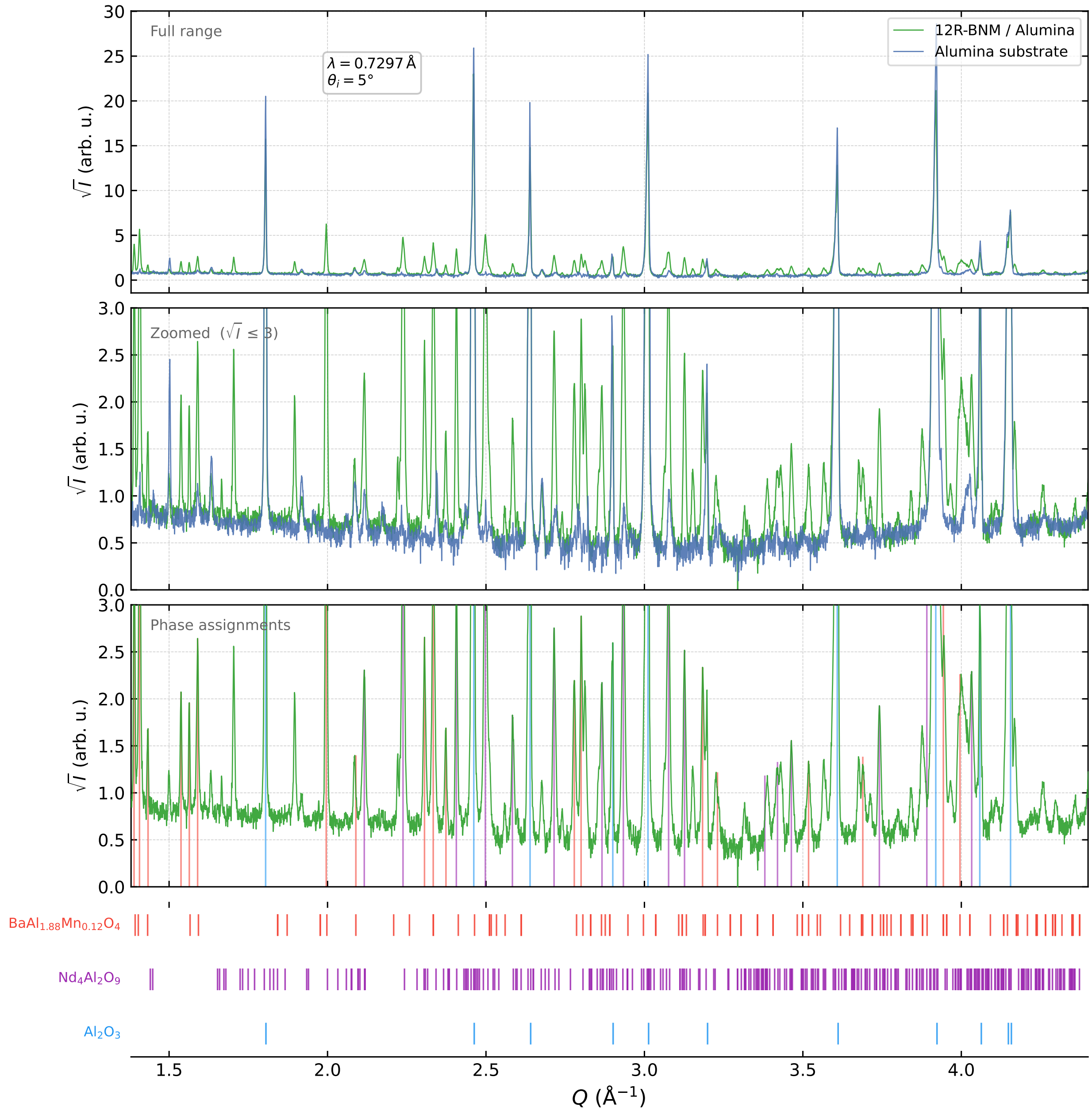

Figure S5: Grazing-incidence X-ray diffraction patterns (incident angle $\theta_i = 5°$, energy 17 keV, $\lambda = 0.7297$ Å) of the bare alumina substrate (MSE Supplies; claimed purity > 99%) and the same substrate following annealing of powdered, non-pelletised 12R-$Ba_4NdMn_3O_{12}$ (12R-BNM) at 1320°C for 14 hrs in air. The upper panel shows the full intensity range; the middle panel shows the same data with the intensity axis capped at $\sqrt{I} \leq 3$ (arb. u.) to reveal weaker reflections. The lower panel shows predicted peak positions for the three phases identified by Pawley refinement: Al_2O_3 (corundum), $Nd_4Al_2O_9$, and $BaAl_{1.88}Mn_{0.12}O_4$, indicating partial decomposition of 12R-BNM and reaction with the alumina substrate during annealing. Several peaks remain unassigned within this three-phase model, and we note that the $Nd_4Al_2O_9$ assignment is tentative given the large number of peaks.

S3 Structural and magnetic characterisation of 12R-BNM-S1

As discussed in Methods, two samples of 12R-BNM were synthesised, 12R-BNM-S1 and 12R-BNM-S2. All data in the main text were obtained for 12R-BNM-S2. In this section of the SI, we present the structural and magnetic characterisation of 12R-BNM-S1.

Figure S6 shows the Rietveld refinement of synchrotron X-ray diffraction data on 12R-BNM-S1. Like 12R-BNM-S2, there is clearly a significant fraction of $Ba_4Mn_3O_{10}$ impurity phase. This Rietveld refinement gives lattice parameters for 12R-BNM-S1 $a = 5.82264(19)$ Å and $c = 28.8389(10)$ Å.

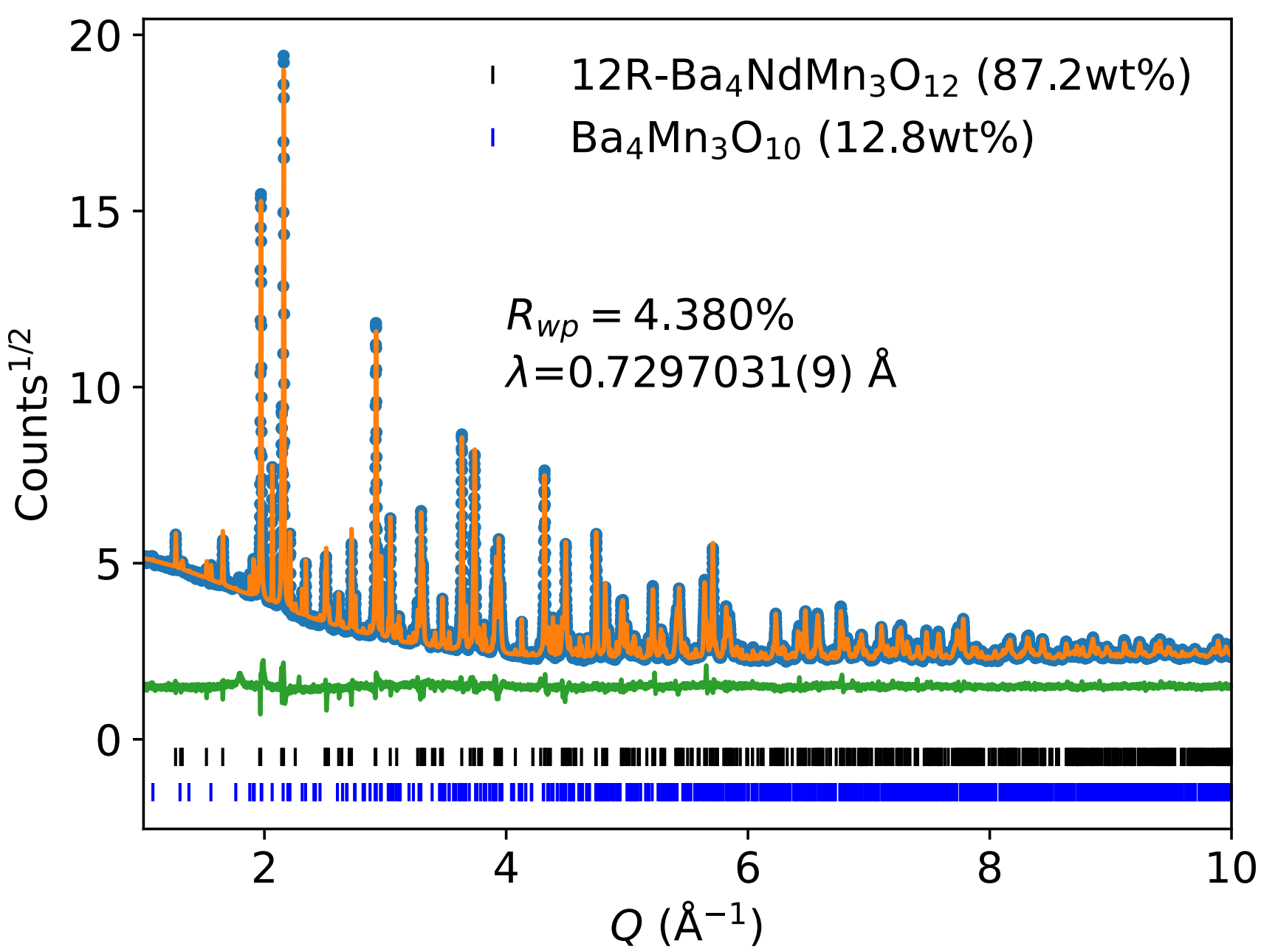

Figure S6: Synchrotron XRD of 12R-BNM-S1 at room-temperature, with calculated data based on Rietveld[9] refinement.

Figure S7 shows the variable-temperature DC magnetic susceptibility of 12R-BNM-S1 in applied fields of 0.1 kOe, 1 kOe, and 50 kOe, and Figure S8 shows the inverse of this same data. These measurements are consistent with those performed for 12R-BNM-S2. Interestingly, we see that at low field (0.1 kOe), the moment per formula unit (inversely proportional to high-temperature linear gradient) is higher for the ZFC sample than the

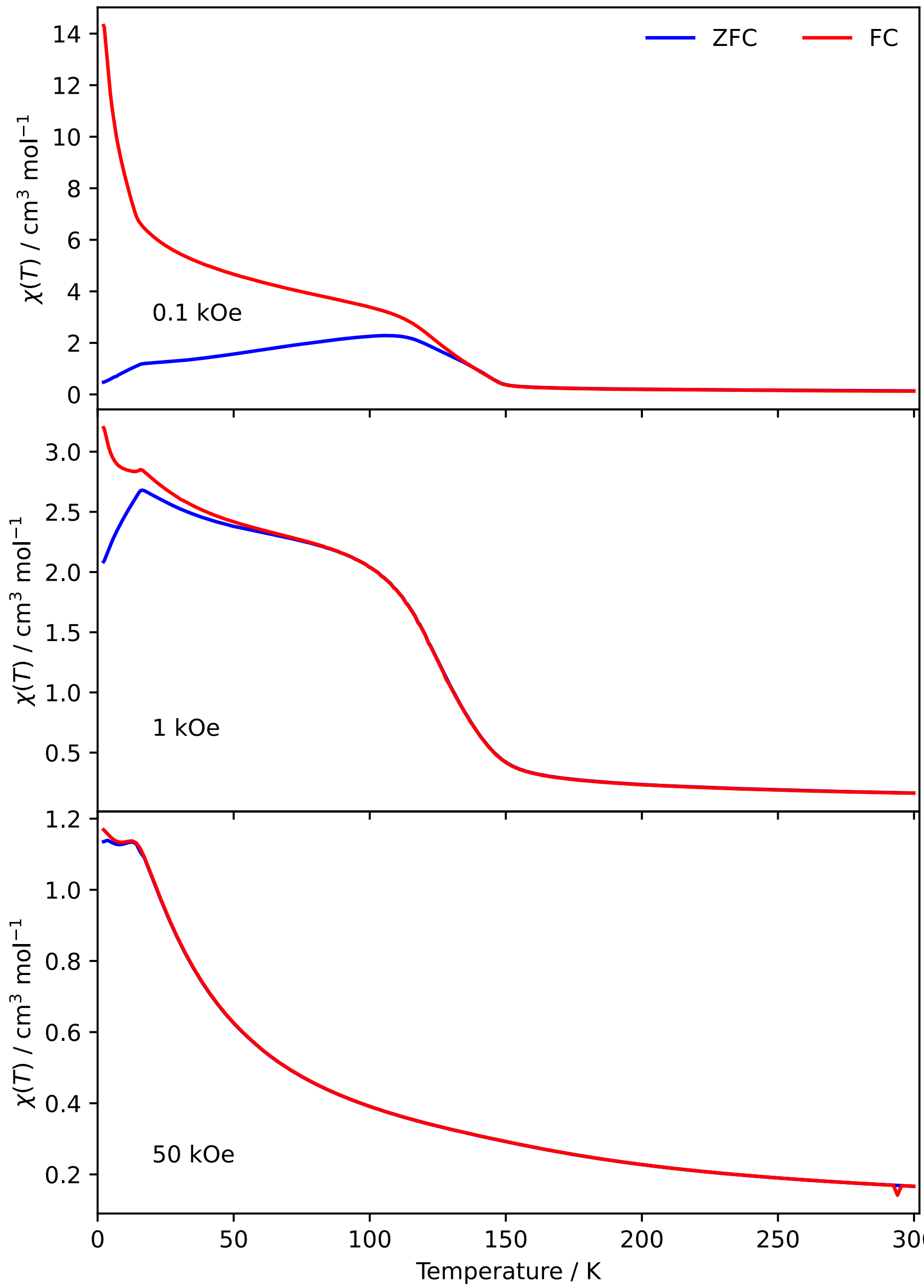

Figure S7: DC magnetic susceptibility as a function of temperature under applied fields of 0.1 kOe, 1 kOe, and 50 kOe, for 12R-BNM-S1. For FC-W (field-cooled, measured on warming) measurements, a high field of 10 kOe was used during the cooling before measurement.

FC sample. This is likely a "memory effect", whereby the field-cooling induces a locally correlated arrangement of magnetic clusters (perhaps medium range order) which prevents clusters from behaving as non-interacting units and inhibits the effective moment. The data measured on heating after zero-field-cooling is free of this effect due to the lack of magnetic history.

Additional magnetometry measurements were also performed. Figure S9 shows the variable-field DC magnetic susceptibility on 12R-BNM-S1. Figure S10 shows the AC susceptibility of 12R-BNM-S1, where no frequency-dependence is resolved within the temperature resolution and frequency range of our measurements.

Figure S11 compares the temperature-dependence of heat capacity $\frac{C_{\mathrm{mag}}}{T}$ for both samples of 12R-BNM over various fields. There is a clear difference in the magnitude of the heat capacity at the top of the bump; this is most likely attributable to a mass error.

Powder neutron diffraction data on 12R-BNM-S1 were collected on the WAND^2 powder diffractometer, Figure S12, at 21.8 K (above the ordering temperature) and 5 K (below the ordering temperature). Additional peaks emerged, consistent with the magnetic peaks observed in the main text on 12R-BNM-S2 and attributed to the reported magnetic structure. However, because the sample was very small ($\sim$0.2 g) the resolution and data quality were insufficient for structure solution, so extensive analysis was only performed on the POWGEN data measured on 12R-BNM-S2 shown in the main text.

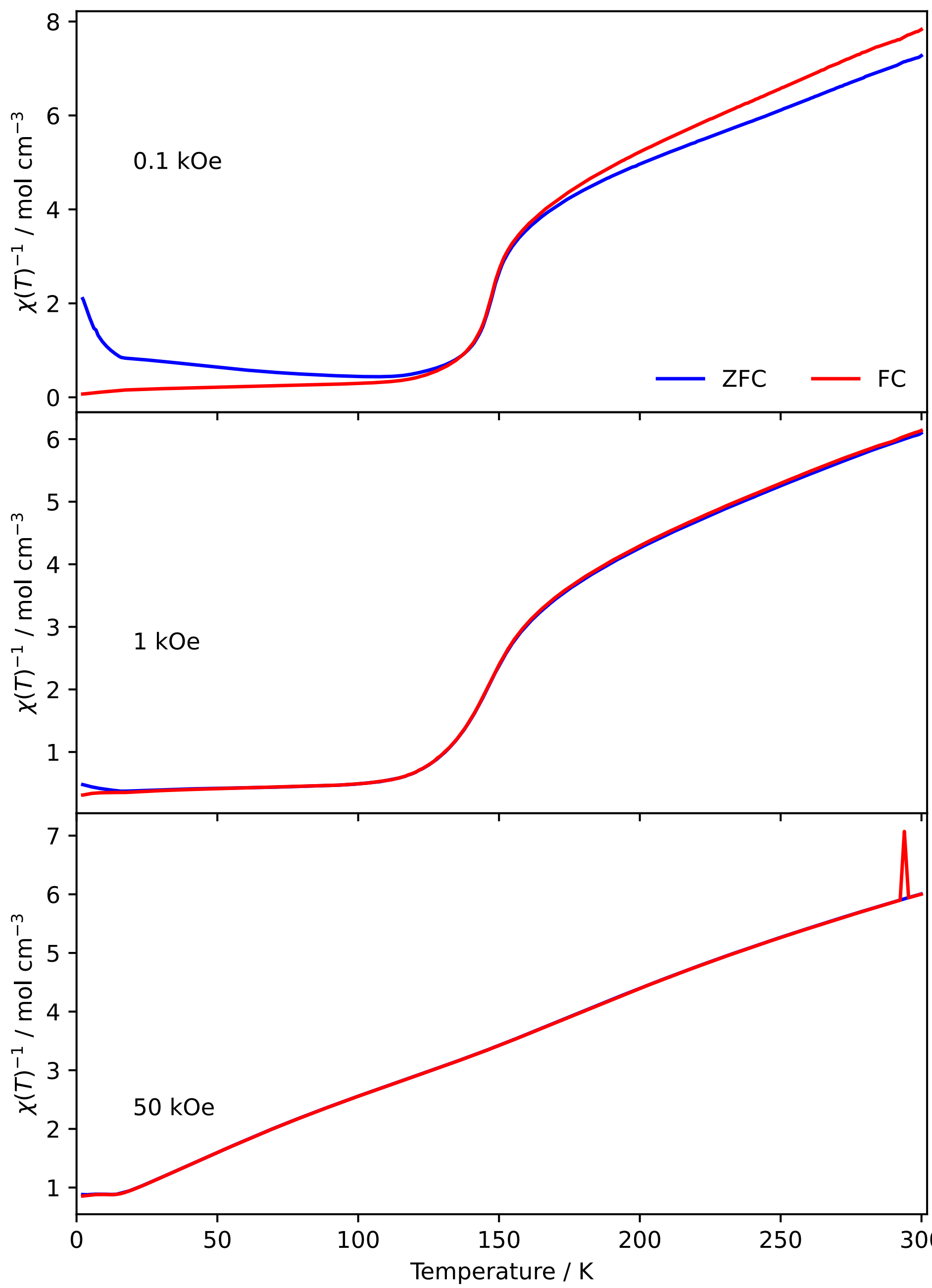

Figure S8: Inverse DC magnetic susceptibility for 12R-BNM-S1. For FC-W (field-cooled, measured on warming) measurements, a high field of 10 kOe was used during the cooling before measurement.

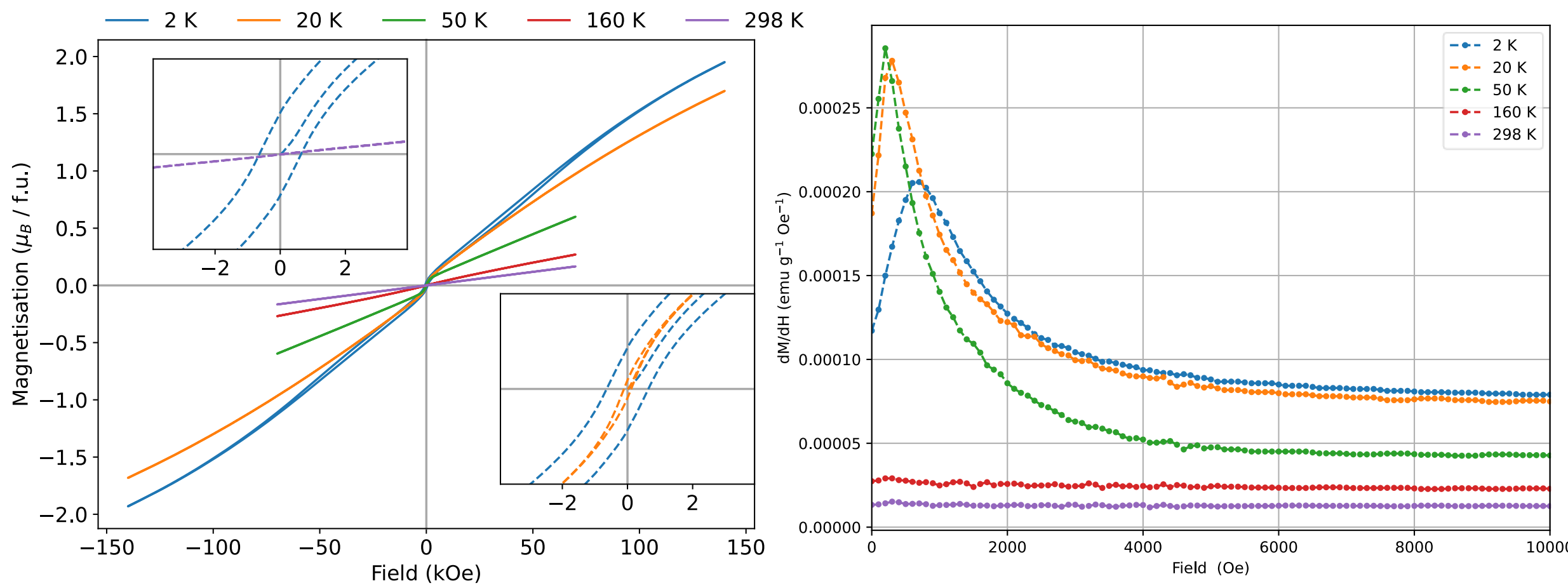

Figure S9: Left: DC magnetic moment as a function of applied field, with insets showing hysteresis loops, for 12R-BNM-S1. Right: The gradient of magnetisation with field for the first quadrant of the measured hysteresis loop.

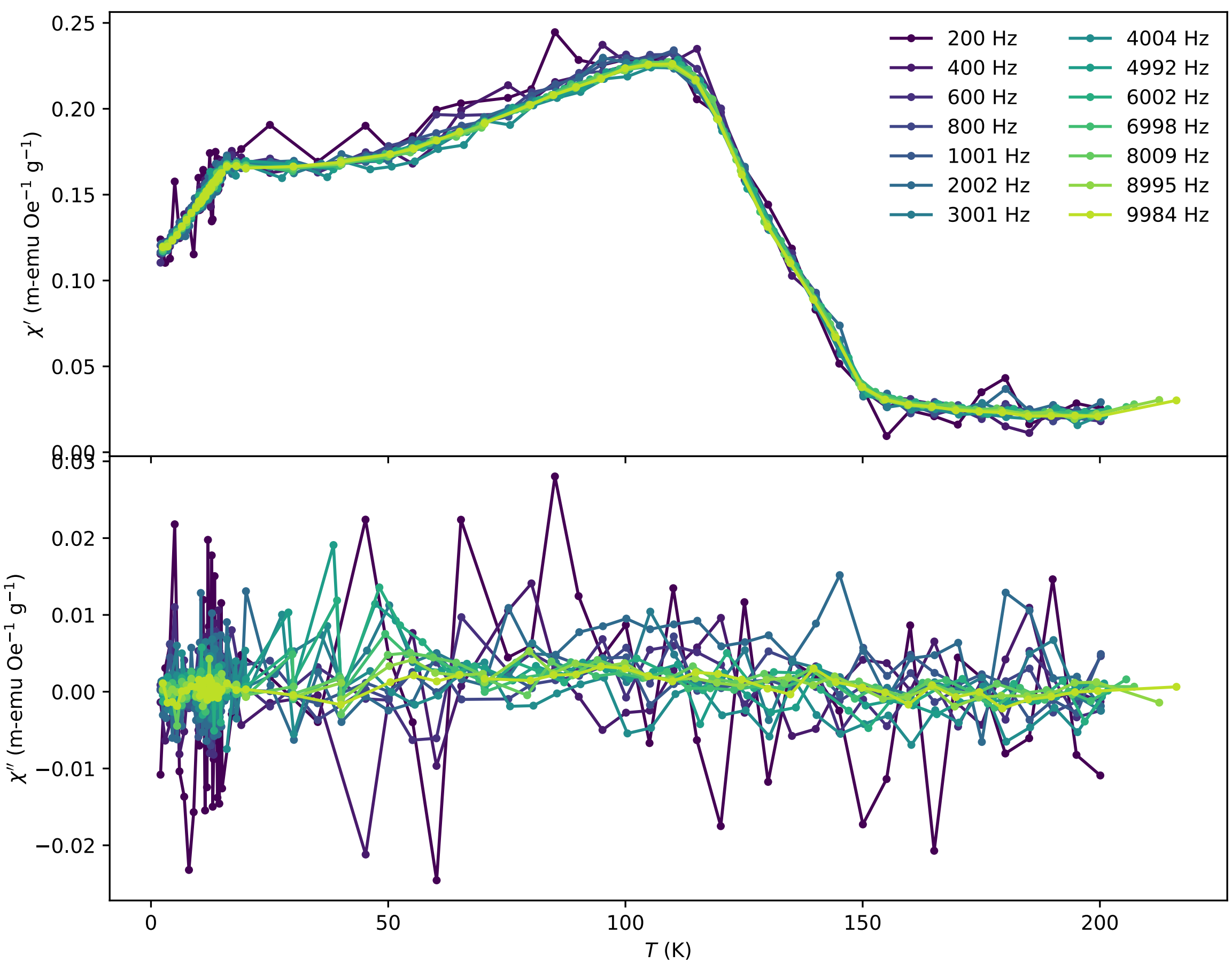

Figure S10: Real χ' and imaginary χ'' components of AC susceptibility data as a function of temperature in an oscillating incident field at frequencies of 200, 400, 600, 800, 1001, 2002, 3001, 4004, 4992, 6002, 6998, 8009, 8995, and 9984 Hz for 12R-BNM-S1.

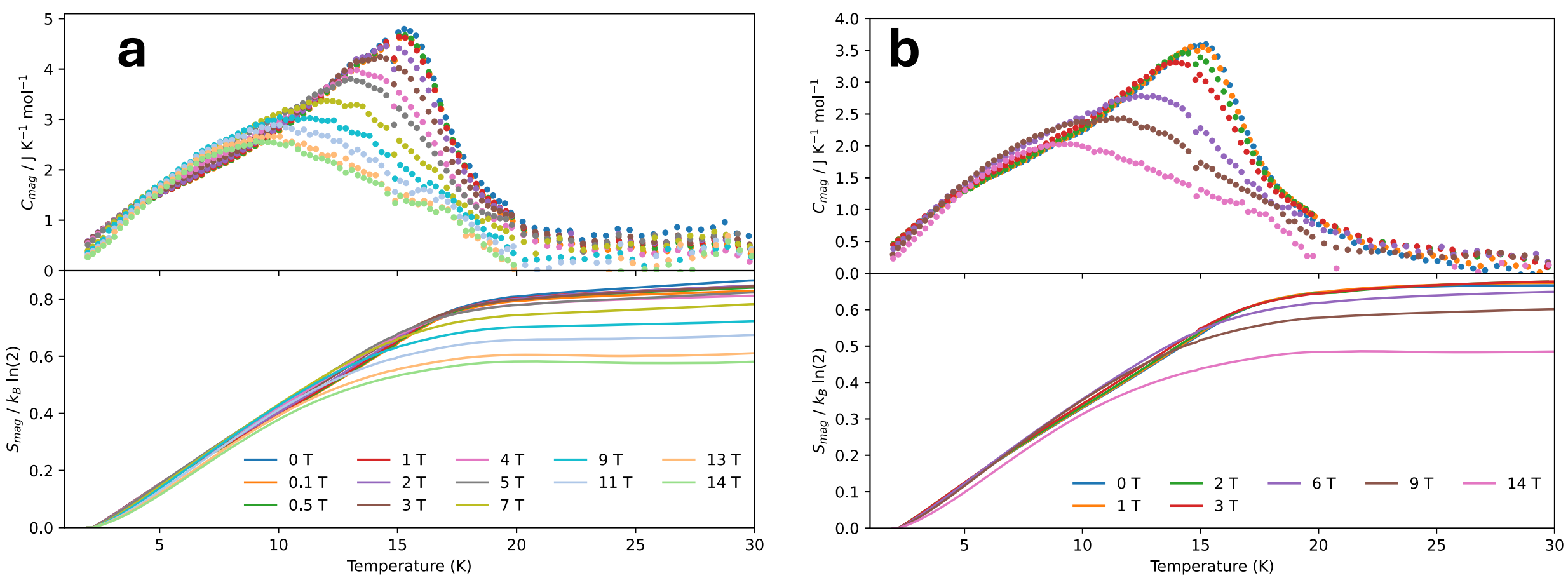

Figure S11: A comparison of magnetic heat capacity and entropy for (a) 12R-BNM-S1 and (b) 12R-BNM-S2.

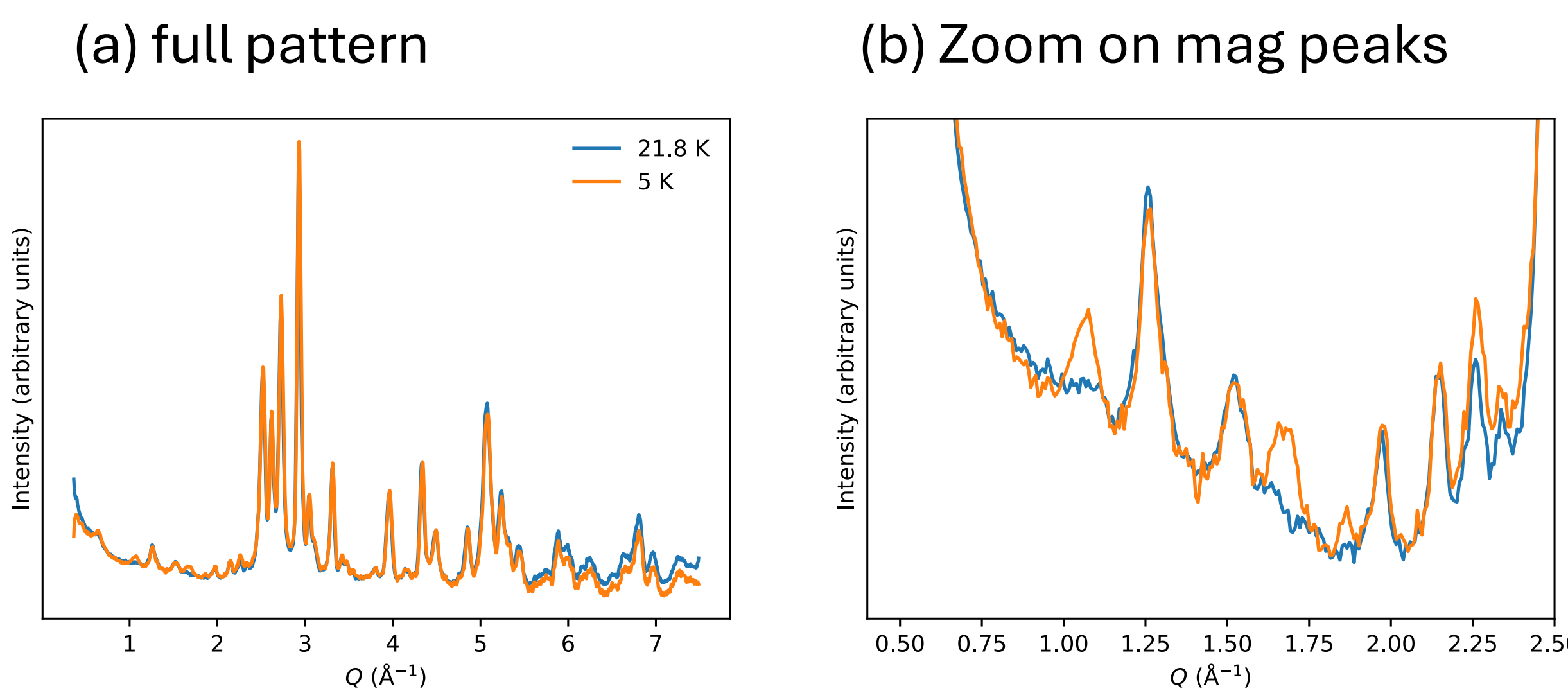

Figure S12: The powder neutron diffraction data of 12R-BNM-S1 presented (a) over the full range and (b) in the vicinity of the magnetic neutron peaks, at 21.8 K (blue) and 5 K (orange). Data were measured on WAND^2 at HFIR, Oak Ridge, with wavelength $\lambda = 1.48642(5)$ Å. The resolution and data quality were insufficient for structure solution due to the small sample size, so extensive analysis was only performed on the POWGEN data measured on 12R-BNM-S2 shown in the main text.

S4 Thermogravimetric analysis

For water splitting material screening, the sample was heated to 1350 °C (10 °C/min) under Ar, held isothermally for 30 minutes, then cooled to 400 °C (25 °C/min), and held isothermally for 30 minutes to ensure reduction. For re-oxidation, the gas was then changed to a mixture of air (80%) and argon (20%), and the sample was heated to 1100 °C (10 °C/min), held isothermally for 30 minutes, then cooled to 200 °C (25 °C/min). The results of this measurement are shown in Figure S13.

To reduce the material, the sample was heated to 1350 °C (20 °C/min), holding isothermally for 1 hour, cooling to 50 °C (50 °C/min), then holding isothermally for 30 minutes under argon gas. After the isothermal hold at 50 °C for 30 minutes, the atmosphere was changed to a mixture of air (80%) and argon (20%) to ensure no mass gain, i.e. due to oxidation, was observed. The results of this measurement are shown in Figure S14.

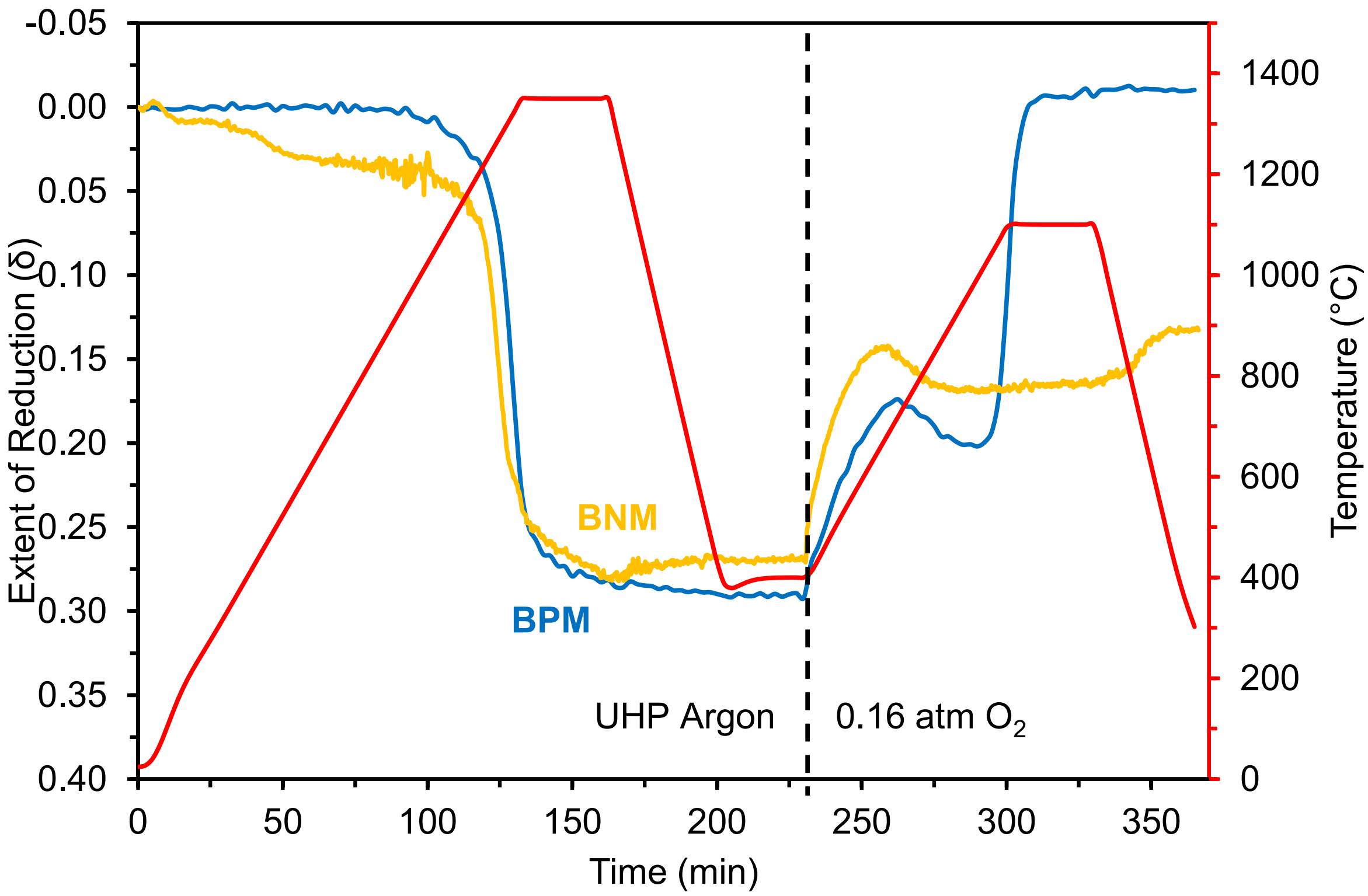

Figure S13: A thermogravimetric analysis measurement on 12R-BNM-S1, with comparison to the Pr-containing analog, as a function of time under varying temperature (with temperature shown as a red plot). The measurement was partially performed under ultra-high pressure (UHP) argon, and upon cooling under an Ar/O_2 mixture.

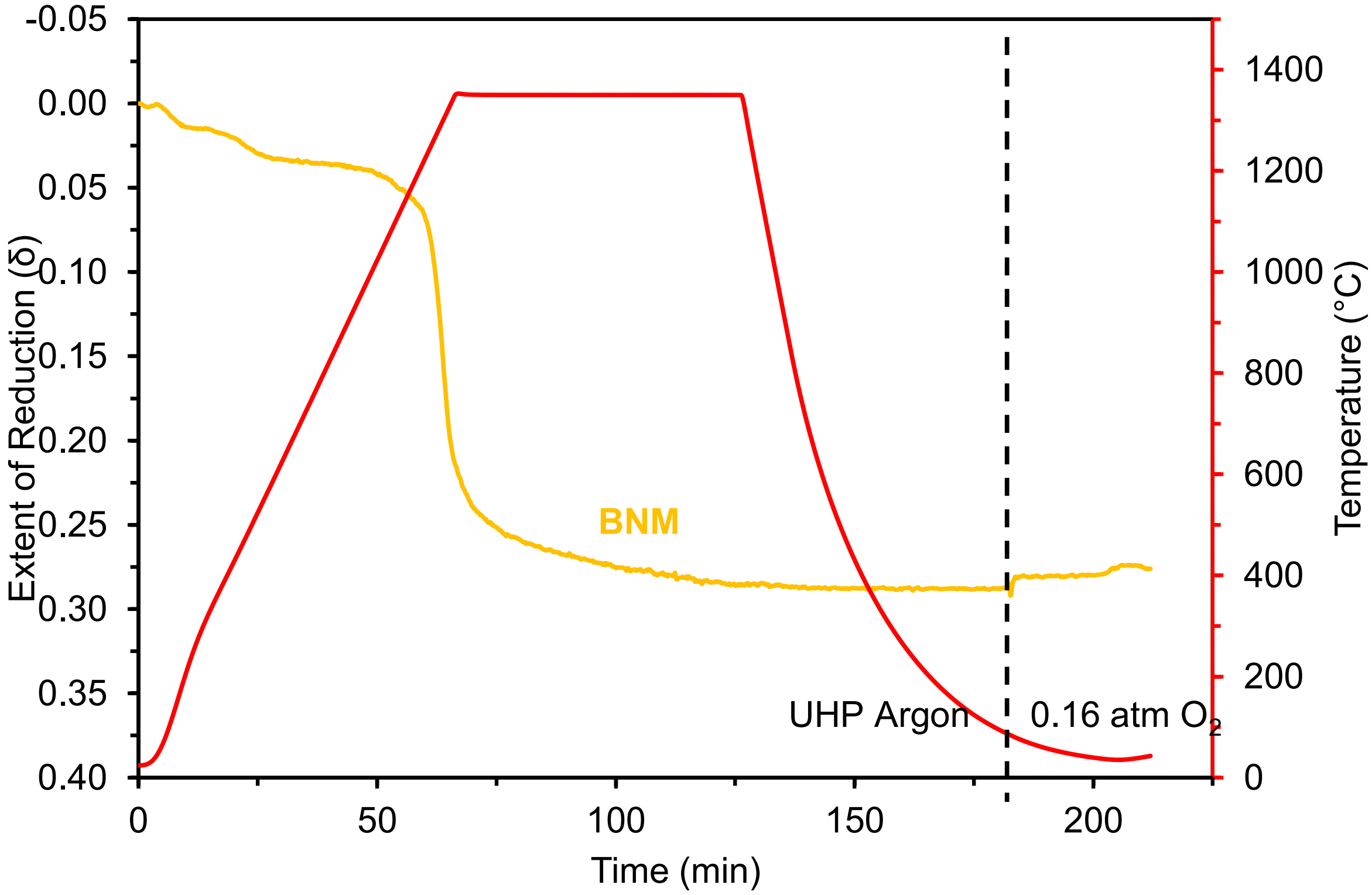

Figure S14: A thermogravimetric analysis measurement on 12R-BNM-S1 as a function of time under varying temperature (with temperature shown as a red plot). The measurement was partially performed under ultra-high pressure (UHP) argon, and later under an Ar/O_2 mixture.

S5 Low-temperature synchrotron X-ray diffraction

We performed low-temperature synchrotron X-ray diffraction using a capillary cryostream cell on beamline 2-1 at Stanford Synchrotron Radiation Lightsource. Measurements were performed between 80 K and 300 K. Increments were 5 ° increments between 80 K and 180 K and 10 ° between 180 K and 300 K. Due to uncertainty in the accuracy in temperature reported by our air blower, we performed the measurement twice: once with a silica (SiO_2) calibrant and once with our 12R-BNM-S2 sample. For each target temperature, we obtained by Rietveld refinement the lattice parameters of silica, and then used this to calculate the true temperature. Sequential Rietveld refinement was performed on the 12R-BNM sample to obtain its properties as a function of the true, silica-derived temperature.

S5.1 Temperature determination from silica

For temperature determination, we used the temperature dependence of lattice parameters for silica as tabulated by Pein *et al.*[10] We reproduce their tabulated values below:

Table S1: Polynomial coefficients describing the temperature-dependent thermal expansion of quartz along the crystallographic a and c directions. The valid temperature interval for the parameterization is 50–800 K, with the reference temperature $T_{\text{ref}} = 0$ K. Data taken from Pein *et al.*[10]

Axis	T interval (K)	T_{ref} (K)	a_0	a_1 (K^{-1})	a_2 (K^{-2})	a_3 (K^{-3})
a	50–800	0	-0.29	8.987×10^{-4}	-1.690×10^{-7}	1.633×10^{-9}
c	50–800	0	-0.136	3.121×10^{-4}	3.174×10^{-7}	6.854×10^{-10}

These parameters are used in the following equation:

$$\frac{\Delta L(T)}{L_0} = a_0 + a_1(T - T_{\text{ref}}) + a_2(T - T_{\text{ref}})^2 + a_3(T - T_{\text{ref}})^3 \tag{S1}$$

where $\Delta L(T) = L(T) - L_0$, $L(T)$ is the cell dimension at temperature T, T is the temperature in K, L_0 is the length at the 293 K, T_{ref} is the reference temperature in K ($T_{\text{ref}} = 0$ K), and a_0, a_1, a_2, and a_3 are the coefficients of thermal expansion.

Temperature calibration gave essentially the same results using both lattice parameters. The temperature values we used are based on the values obtained from the a lattice parameter of silica.

S5.2 Results of sequential Rietveld refinement on 12R-BNM

Lattice parameters with temperature are shown in Figure S15.

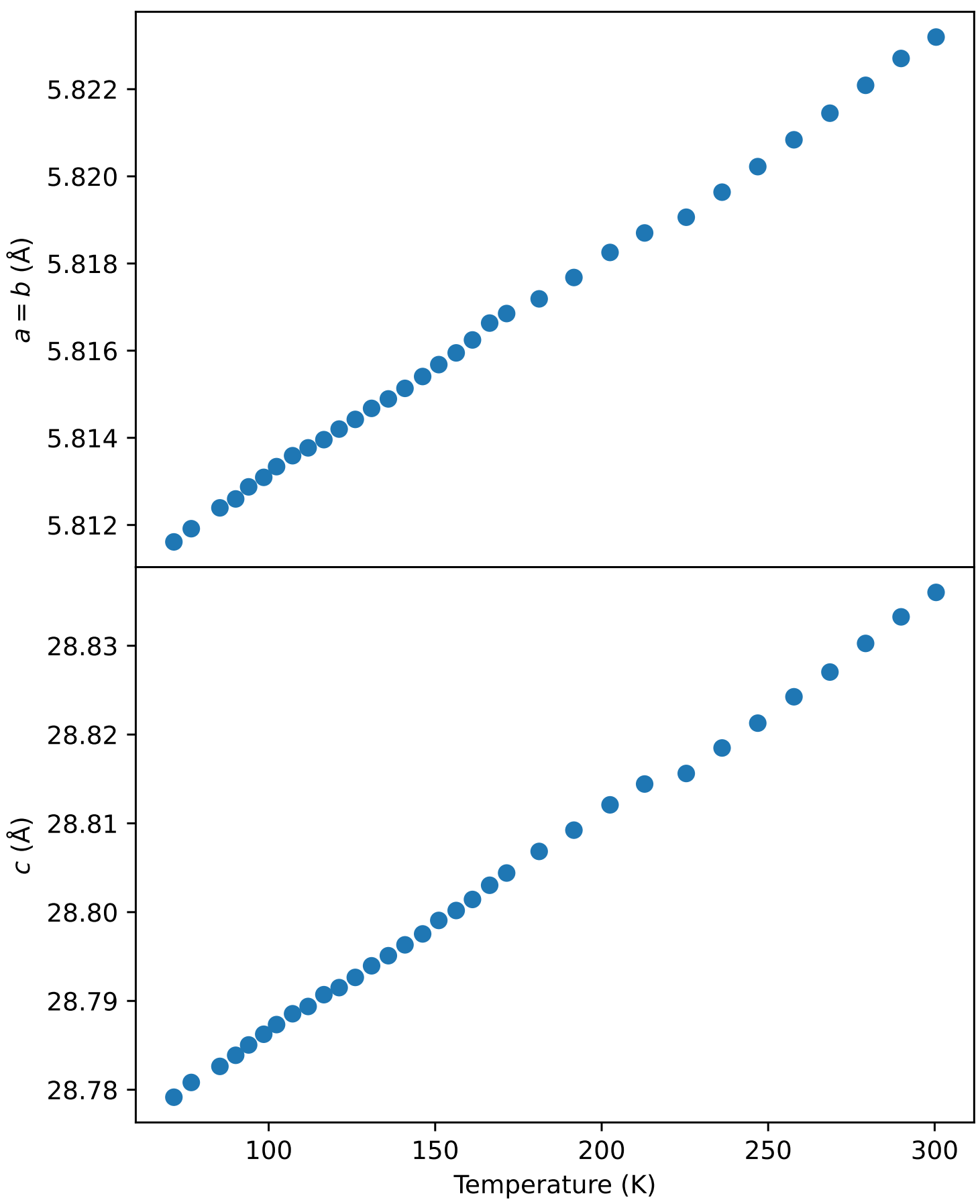

Figure S15: Temperature-dependence of lattice parameters in 12R-BNM-S2 obtained by sequential Rietveld refinement to low-temperature synchrotron X-ray diffraction data. Data were collected on beamline 2-1 at the Stanford Synchrotron Radiation Lightsource, using a capillary cryostream cell. Temperature were calibrated using a silica (SiO_2) standard, with the temperature dependence of lattice parameters for silica described by the coefficients in Table S1 for Equation S1 taken from Pein *et al.*[10]

S6 Rietveld refinements of neutron diffraction data

In this section we show the results of some additional Rietveld refinements[9] to the neutron diffraction data from the POWGEN instrument[11] on sample 12R-BNM-S2. It was necessary to determine O vacancy content, which was performed by Rietveld refinement against the 200 K neutron diffraction data, where the material is paramagnetic and therefore the data can be fit using just nuclear phases. Table S2 shows the O vacancy content obtained by Rietveld refinement to the neutron diffraction data, and the combined X-ray/neutron data, under each scenario at 200 K. In all neutron Rietveld refinements described here, including the 200 K vacancy refinements and the lower-temperature magnetic refinements, we model thermal motion and atomic disorder using anisotropic displacement parameters. Based on these results, we conclude that there are certainly vacancies on at least some of the O sites. However, there are significant features in the difference patterns from these Rietveld refinements, and so we are not certain that the refined vacancy parameters are quantitatively accurate.

In subsequent fits to the neutron diffraction data at 60 K, 20 K and 8 K, where the material is magnetically ordered, the O vacancy content was fixed to the values obtained from the Rietveld refinement at 200 K. We use the O vacancy content obtained for the scenario where there is a single O vacancy parameter across both sites, as there seems to be negligible improvement in R_{wp} from decoupling these values. The refined lattice parameters, atomic coordinates, occupancy factors, and anisotropic displacement parameters for the 200 K neutron refinement with a single O vacancy parameter are provided in Table S3.

Figure S16 and S19 shows the results of Rietveld refinement of the neutron diffraction data collected at 200 K and 60 K, respectively, where the material and its $Ba_4Mn_3O_{10}$ impurity are both paramagnetic. Data are fit therefore using two nuclear phases: 12R-BNM ($R\bar{3}m$ in the hexagonal setting) and $Ba_4Mn_3O_{10}$ ($Cmca$, using the crystal structure reported by Zubkov *et al.*[12]).

We additionally performed combined Rietveld refinement of O vacancies against both

Table S2: Oxygen vacancy content from Rietveld refinement of POWGEN neutron diffraction data on 12R-BNM-S2. In the single parameter case, oxygen vacancy content of both O sites is refined but fixed equal. In the two parameters case, the vacancy contents are refined separately for both sites. "Dataset" refers to the diffraction data being refined, and is either neutron or "Combined" which indicates a combined refinement of the neutron diffraction data with the synchrotron data from beamline 2-1 at SSRL.

**Dataset**	**O vacancy model**	**Site**	**O vacancies (%)**	R_{wp} (%)
Neutron	None	Both O sites	0.0	5.714
	Single parameter	Both O sites	3.66(14)	5.188
	Two parameters	O1	4.1(2)	5.185
		O2	3.2(3)	
		Mean	3.66(18)	
Combined	None	Both O sites	0.0	6.304
	Single parameter	Both O sites	4.1(3)	6.277
	Two parameters	O1	4.9(4)	6.274
		O2	3.2(4)	
		Mean	4.0(3)	

the synchrotron (beamline 2-1) and neutron (POWGEN) diffraction data at 200 K, with the resulting values included in Table S2. Figure S17 shows the Rietveld refinement assuming a single oxygen vacancy parameter for both O sites, and Figure S18 shows the Rietveld refinement assuming each O site has a unique vacancy parameter. The results are qualitatively the same as what is found for refinement just against neutron diffraction data.

Figure S20 shows the result of Rietveld refinement of the neutron diffraction data collected at 20 K, using these two nuclear phases, plus a magnetic phase to account for the magnetic ordering of the $Ba_4Mn_3O_{10}$ phase ($Pb'c'a$, or #61.436, using the BNS notation[13]).

Figure S21 shows the Rietveld refinement of the data at 8 K, including our proposed magnetic structure for 12R-BNM, where O vacancy concentrations at the O1 and O2 sites are fixed to the values obtained from the refinement at 200 K. A close-up of the magnetic peaks for this latter case is shown in Figure 8.

Table S4 shows the magnetic structure and moment magnitudes we obtain for the 12R-BNM phase at 8 K. Magnetic moment and vacancy content both impact peak intensities and will correlate with eachother when there is overlap between magnetic and structural Bragg peaks, as is the case for 12R-BNM. For this reason, O vacancy content in this mag-

Table S3: Refined crystallographic parameters from Rietveld refinement of the 200 K POWGEN neutron diffraction data for 12R-BNM-S2. Only non-unity occupancy factors are reported; the O1 and O2 occupancies were constrained to be equal. Anisotropic displacement parameters are in Å^2. Estimated standard deviations are shown in parentheses. The corresponding fit is shown in Figure S16.

**Item**	**Value**					
Compound	$Ba_4NdMn_3O_{12-\gamma}$					
Space group	$R\bar{3}m$					
Unit cell	$a = 5.81847(6)$ Å					
	$c = 28.8220(4)$ Å					
	$V = 845.030$ Å^3					
**Atomic coordinates and occupancies**						
Site	Wyckoff	x	y	z	occ	
Ba1	$6c$	0	0	0.13081(7)	1	
Ba2	$6c$	0	0	0.28198(10)	1	
Nd1	$3a$	0	0	0	1	
Mn1	$3b$	0	0	0.5	1	
Mn2	$6c$	0	0	0.41289(9)	1	
O1	$18h$	0.48087(10)	0.51913(10)	0.12547(4)	0.9634(14)	
O2	$18h$	0.48791(11)	0.51209(11)	0.28773(4)	0.9634(14)	
**Anisotropic displacement parameters**						
**Site**	U_{11}	U_{22}	U_{33}	U_{12}	U_{13}	U_{23}
Ba1	0.0108(6)	0.0108(6)	0.0076(11)	0.0054(3)	0	0
Ba2	0.0030(6)	0.0030(6)	0.0441(16)	0.0015(3)	0	0
Nd1	0.0020(5)	0.0020(5)	0.0010(8)	0.0010(2)	0	0
Mn1	0.006(1)	0.006(1)	0.0081(17)	0.0029(5)	0	0
Mn2	0.0025(6)	0.0025(6)	0.0097(13)	0.0013(3)	0	0
O1	0.0084(3)	0.0084(3)	0.0078(4)	0.0047(4)	−0.00010(19)	−0.00010(19)
O2	0.0106(4)	0.0106(4)	0.0223(6)	0.0063(5)	0.0038(2)	0.0038(2)

netic refinement is fixed to the values obtained from the Rietveld refinement to the dataset measured at 200 K.

Table S4: Magnetic structure obtained by Rietveld refinement of the magnetic unit cell against neutron diffraction data collected at 8 K on the POWGEN instrument for 12R-BNM-S2. O vacancy content in these refinements was fixed to the value obtained from Rietveld refinement to the 200 K neutron diffraction data, assuming a single O vacancy parameter for both sites.

**Item**	**Value**
Compound	$Ba_4NdMn_3O_{12}$
Parent space group	$R\bar{3}m$
Propagation vector(s)	$(0, \frac{1}{2}, 1)$
MSG number (BNS)	10.49
Unit-cell parameters	$a = 10.071(11)$ Å
	$b = 5.81197(6)$ Å
	$c = 10.136(11)$ Å
	$\alpha = 90°$
	$\beta = 109.27(9)°$
	$\gamma = 90°$
**MSG symmetry operations**	
	$-x, -y, -z, \ +1$
	$-x, y, -z, \ +1$
	$-x + \frac{1}{2}, -y + \frac{1}{2}, -z, \ -1$
	$-x + \frac{1}{2}, y + \frac{1}{2}, -z, \ -1$
	$x, -y, z, \ +1$
	$x, y, z, \ +1$
	$x + \frac{1}{2}, -y + \frac{1}{2}, z, \ -1$
	$x + \frac{1}{2}, y + \frac{1}{2}, z, \ -1$
**Non-magnetic atom positions**	
Ba1	Ba at $4i(0.13100, \ 0.00000, \ 0.39300)$
Ba2	Ba at $4i(0.28206, \ 0.00000, \ 0.84618)$
O1_1	O at $8j(0.88447, \ 0.27736, \ 0.37605)$
O1_2	O at $4i(0.60711, \ 0.00000, \ 0.37605)$
O2_1	O at $8j(0.04392, \ 0.26851, \ 0.86325)$
O2_2	O at $4i(0.77541, \ 0.00000, \ 0.86325)$
**Magnetic atom positions**	
Nd1	Nd at $2a(0, \ 0, \ 0)$
Mn1	Mn at $2d(0, \ 0.5, \ 0.5)$
Mn2	Mn at $4i(0.41288, \ 0, \ 0.23864)$
**Magnetic moments (μ_B), crystal-axis components**	
Nd1	$(0.00000, \ -0.75(14), \ 0.00000)$
Mn1	$(0.00000, \ 0.89(11), \ 0.00000)$
Mn2	$(0.00000, \ 0.99(10), \ 0.00000)$

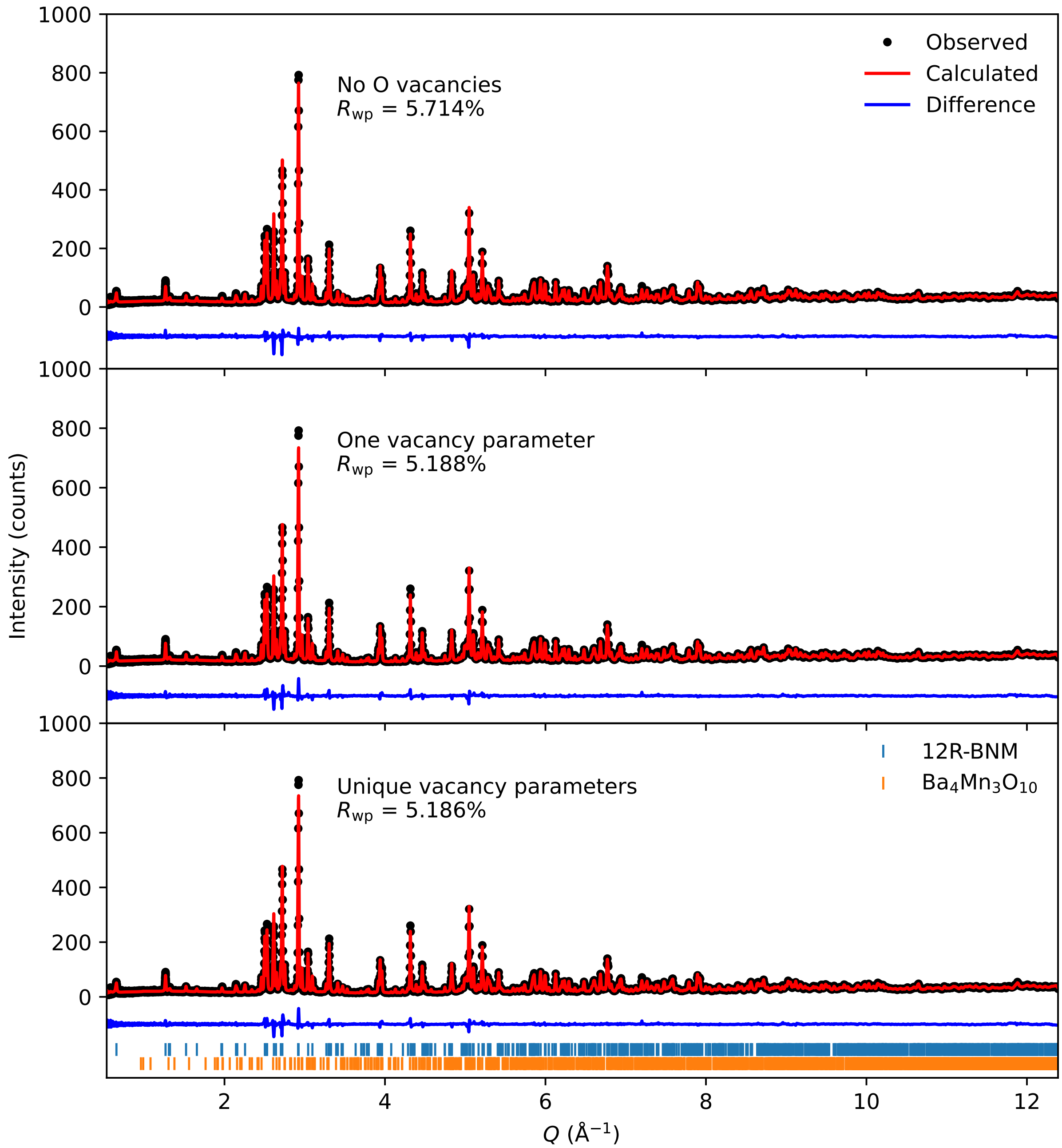

Figure S16: Neutron diffraction data on 12R-BNM-S2 at 200 K obtained from the POWGEN instrument[11] at Oak Ridge National Laboratory, with resulting fit obtained by Rietveld refinement using two nuclear phases: 12R-BNM ($R\bar{3}m$ in the hexagonal setting) and $Ba_4Mn_3O_{10}$ ($Cmca$, using the crystal structure reported by Zubkov *et al.*[12]). Three scenarios were considered: (top) assumed no O vacancies, (middle) a single refined vacancy parameter across both O sites, and (bottom) a unique vacancy parameter for each O site. Vertical tickmarks indicate the position of Bragg reflections.

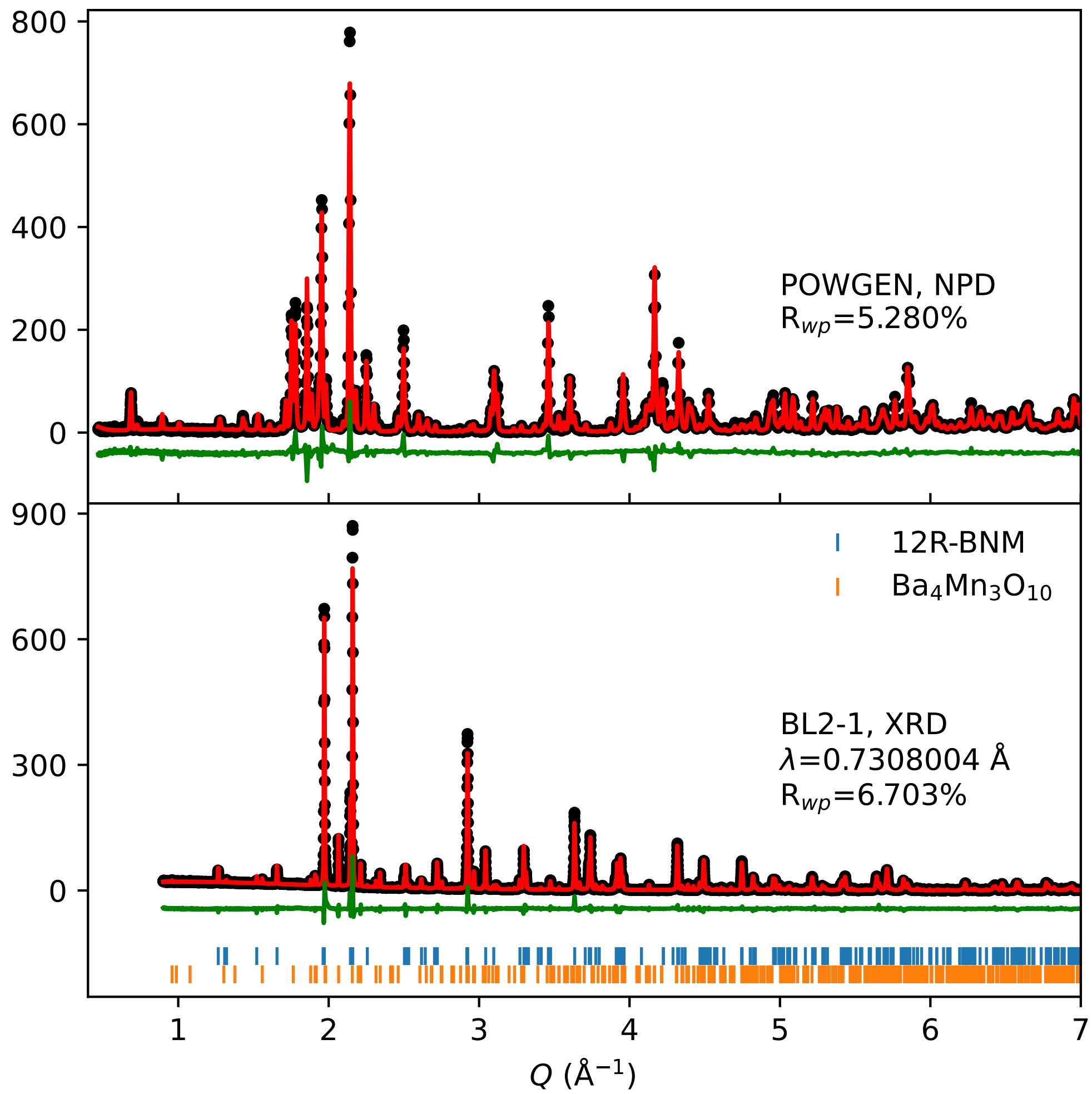

Figure S17: Neutron diffraction data on 12R-BNM-S2 at 200 K obtained from the POWGEN instrument[11] at Oak Ridge National Laboratory, and synchrotron diffraction from beamline 2-1 on the same sample and at the same temperature, with resulting fit obtained by Rietveld refinement using two nuclear phases: 12R-BNM ($R\bar{3}m$ in the hexagonal setting) and $Ba_4Mn_3O_{10}$ ($Cmca$, using the crystal structure reported by Zubkov *et al.*[12]). Vertical tickmarks indicate the position of Bragg reflections.

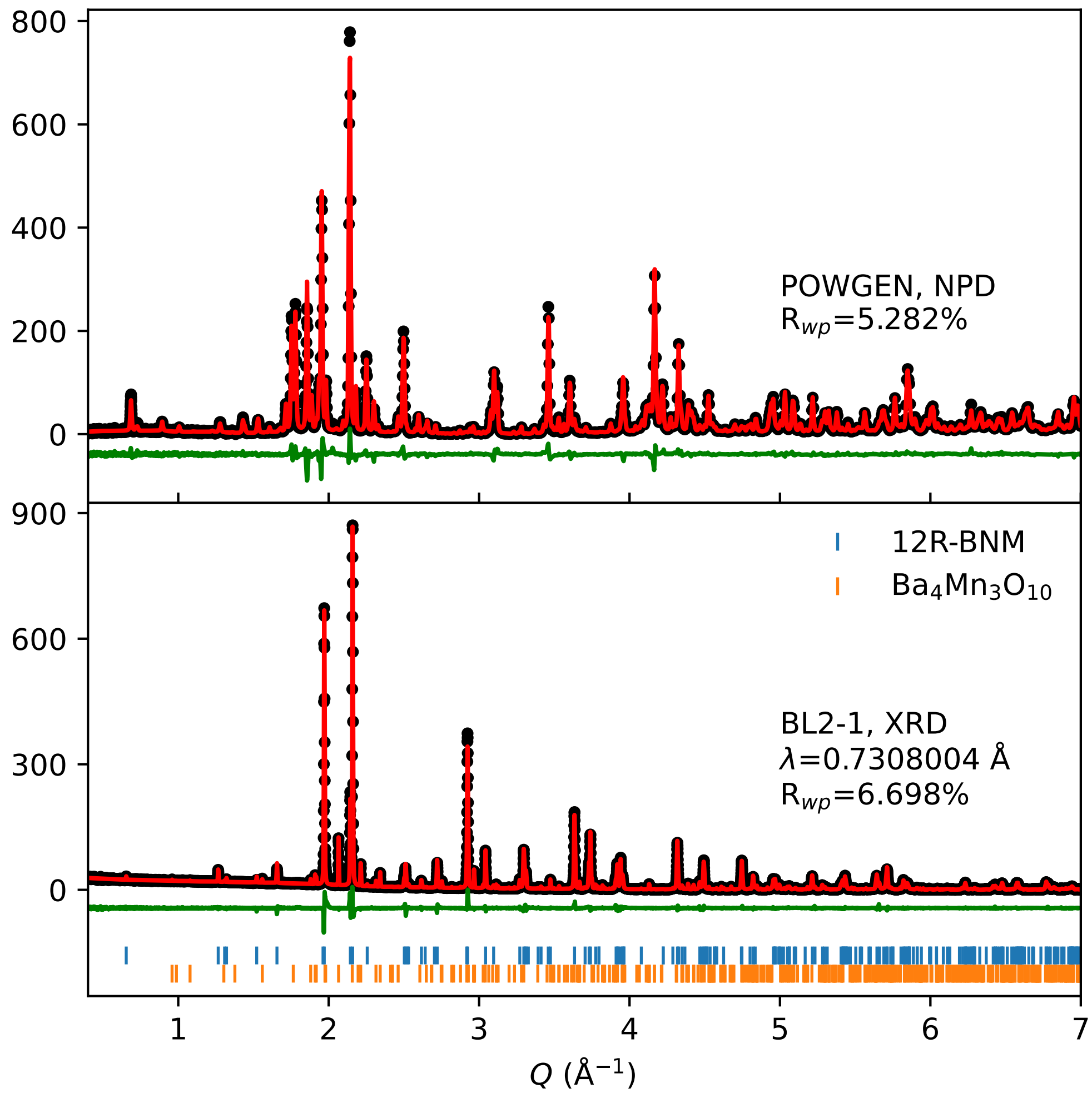

Figure S18: Neutron diffraction data on 12R-BNM-S2 at 200 K obtained from the POWGEN instrument[11] at Oak Ridge National Laboratory, and synchrotron diffraction from beamline 2-1 on the same sample and at the same temperature, with resulting fit obtained by Rietveld refinement using two nuclear phases: 12R-BNM ($R\bar{3}m$ in the hexagonal setting) and $Ba_4Mn_3O_{10}$ ($Cmca$, using the crystal structure reported by Zubkov *et al.*[12]). Vertical tickmarks indicate the position of Bragg reflections. The 12R-BNM model assumes two O vacancy parameters (one for each site).

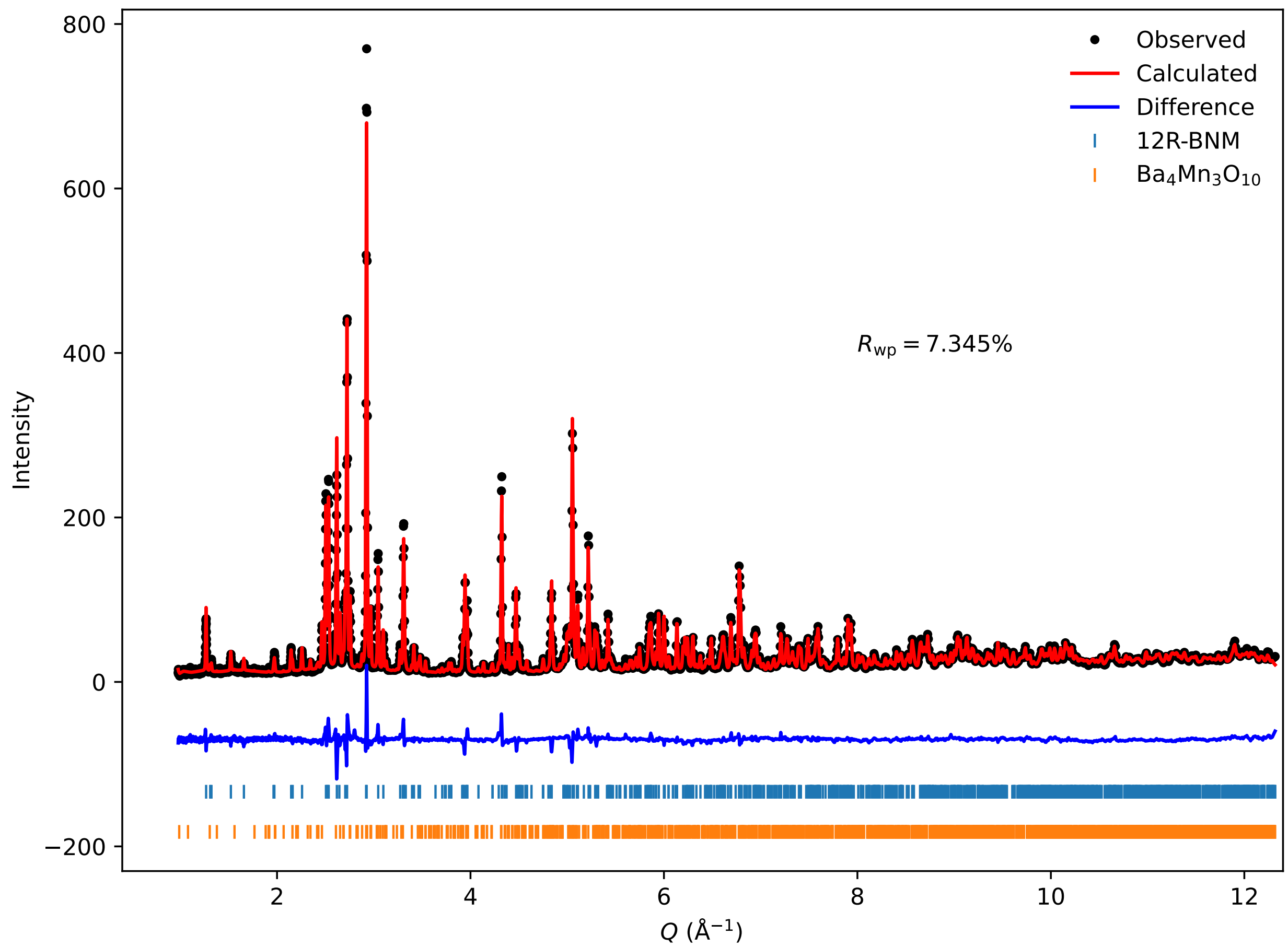

Figure S19: Neutron diffraction data on 12R-BNM-S2 at 60 K obtained from the POWGEN instrument[11] at Oak Ridge National Laboratory, with resulting fit obtained by Rietveld refinement using two nuclear phases: 12R-BNM ($R\bar{3}m$ in the hexagonal setting) and $Ba_4Mn_3O_{10}$ ($Cmca$, using the crystal structure reported by Zubkov *et al.*[12]). O vacancy content was fixed to the value obtained from Rietveld refinement to the 200 K neutron diffraction data, assuming a single O vacancy parameter for both sites. Vertical tickmarks indicate the position of Bragg reflections.

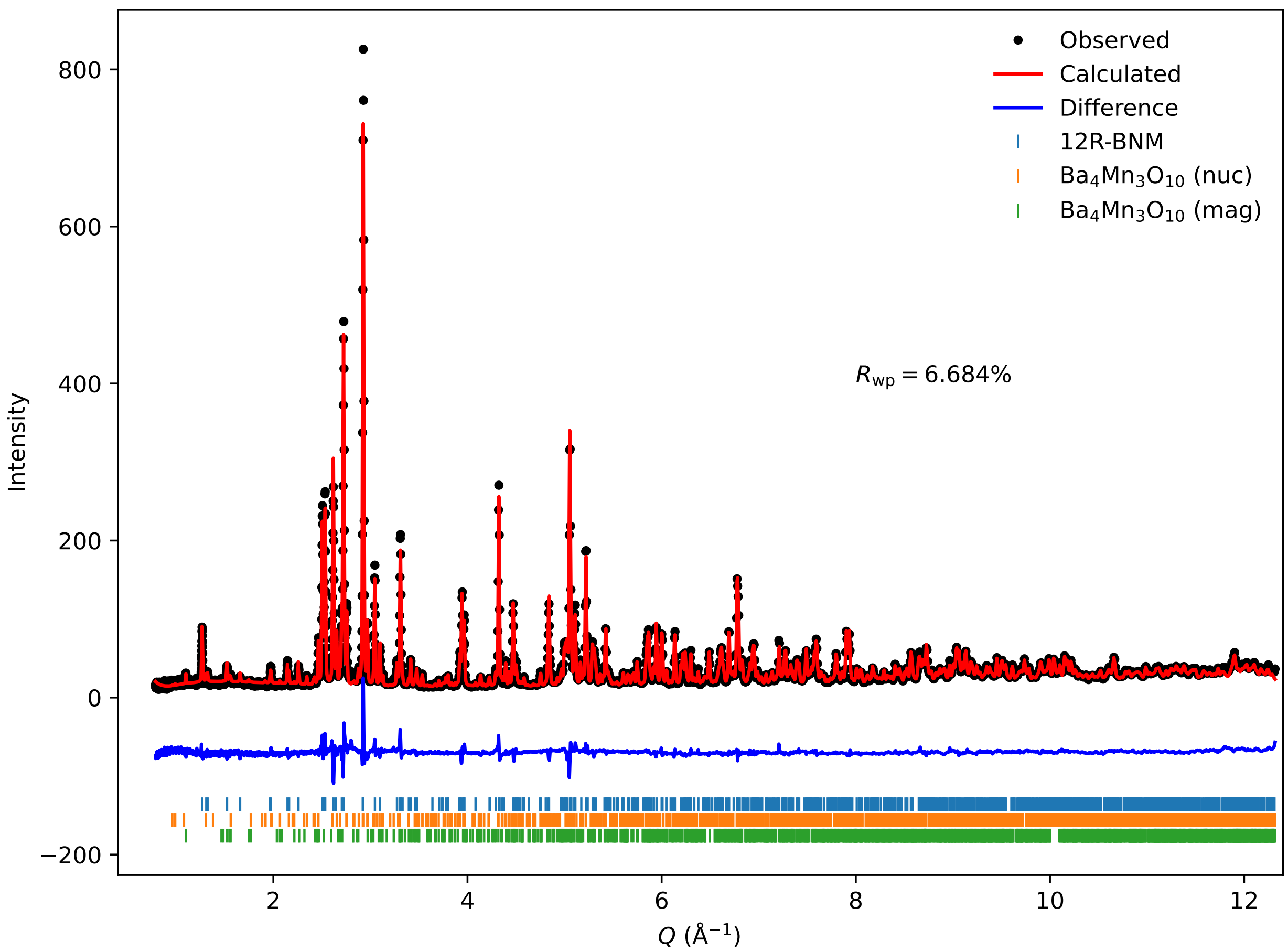

Figure S20: Neutron diffraction data on 12R-BNM-S2 at 20 K obtained from the POWGEN instrument[11] at Oak Ridge National Laboratory, with resulting fit obtained by Rietveld refinement using two nuclear phases: 12R-BNM ($R\bar{3}m$ in the hexagonal setting) and $Ba_4Mn_3O_{10}$ ($Cmca$, using the crystal structure reported by Zubkov *et al.*[12]), and one magnetic phase for $Ba_4Mn_3O_{10}$ ($Pb'c'a$, or #61.436, using the BNS notation[13]). O vacancy content was fixed to the value obtained from Rietveld refinement to the 200 K neutron diffraction data, assuming a single O vacancy parameter for both sites. Vertical tickmarks indicate the position of Bragg reflections.

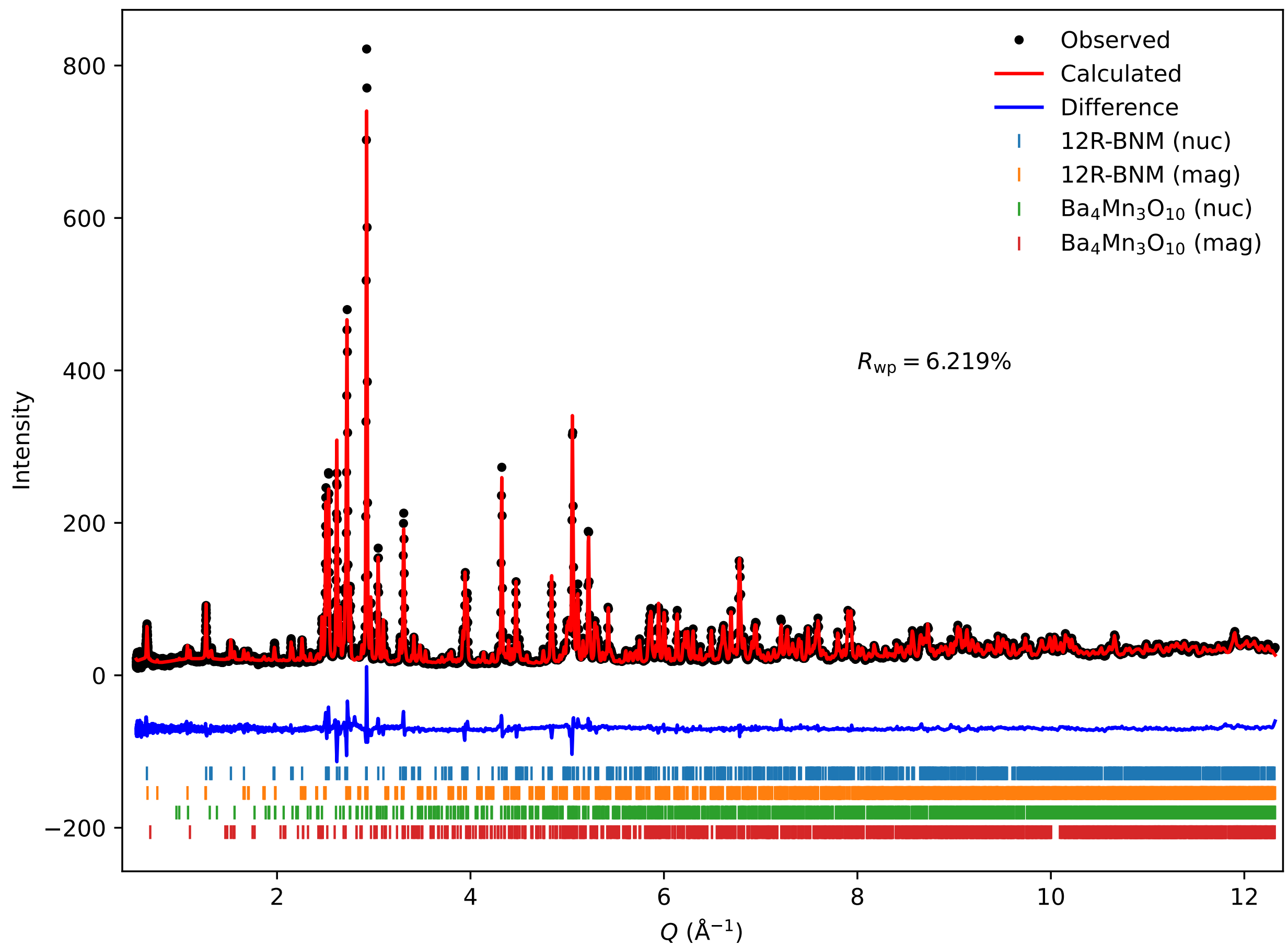

Figure S21: Neutron diffraction data on 12R-BNM-S2 at 8 K obtained from the POWGEN instrument[11] at Oak Ridge National Laboratory, with resulting fit obtained by Rietveld refinement using two nuclear phases: 12R-BNM ($R\bar{3}m$ in the hexagonal setting) and $Ba_4Mn_3O_{10}$ ($Cmca$, using the crystal structure reported by Zubkov *et al.*[12]), and two magnetic phases: $Ba_4Mn_3O_{10}$ ($Pb'c'a$, or #61.436) and our newly-reported magnetic structure for 12R-BNM (P_C2/m, or #10.49 in BNS notation). In this refinement, O vacancy concentration were fixed to that obtained in the Rietveld refinement of the 200 K neutron diffraction dataset. For a zoom on the magnetic peaks, see Figure 8.

S7 X-ray absorption spectroscopy

As discussed in the main text of the manuscript, we have collected XAS data at multiple edges in 12R-BNM-S2. Our experimental data was collected at the Stanford Synchrotron Radiation Lightsource, Beamline 2-2 for hard XAS (Nd L-edge, Mn K-edge) and Beamline 8-2 for soft XAS (Mn L-edge, O K-edge, Ba L-edge, Nd M_4-, and M_5-edge).

The data that is key to the story is presented in Figure 2 in the main text. There, the Mn K-edge, Mn L-edge, Nd L_3-edge, and O K-edge XAS data are presented. Additionally, here we present additional data on this material.

Figure S22 shows the Nd M_4- and M_5-edge XAS data measured on Beamline 8-2. We do not have a reference for Nd at this edge and so the data is presented in isolation.

Figure S23 shows the Nd L_2- and Nd L_1-edges XAS data measured on Beamline 2-2. For these datasets, there were energies at which the diffracting monochromator crystal had multiple reflections resulting in a drop in photon transmission and contributing unwanted features which are not related to the material. Despite these features, the data at these edges is still consistent with conclusions presented for the Nd L_3-edge in the main text, i.e. that Nd is likely in mixed 3+/4+ oxidation states.

Figure S24 shows the Ba L-edge XAS data measured on Beamline 2-2.

These data are prepared by merging together several spectra measured sequentially. Figure S26 shows the merging procedure for the Mn L-edge data used to create the plot shown in Figure 2(c). Figure S27 shows the merging procedure for the Mn K-edge data used to create the plot shown in Figure 2(a). Figure S29 shows the merging procedure for the O K-edge data used to create the plot shown in Figure 2(d). Figure S30(a) shows the merging procedure for the Nd L_3-edge data used to create the plot shown in Figure 2(b); likewise Figure S30(b) and Figure S30(c) show the merging procedures for the far lower-quality Nd L_2- and L_1-edges. Figure S32 shows the merging procedure for the Ba L-edge data used to create the plot shown in Figure S24.

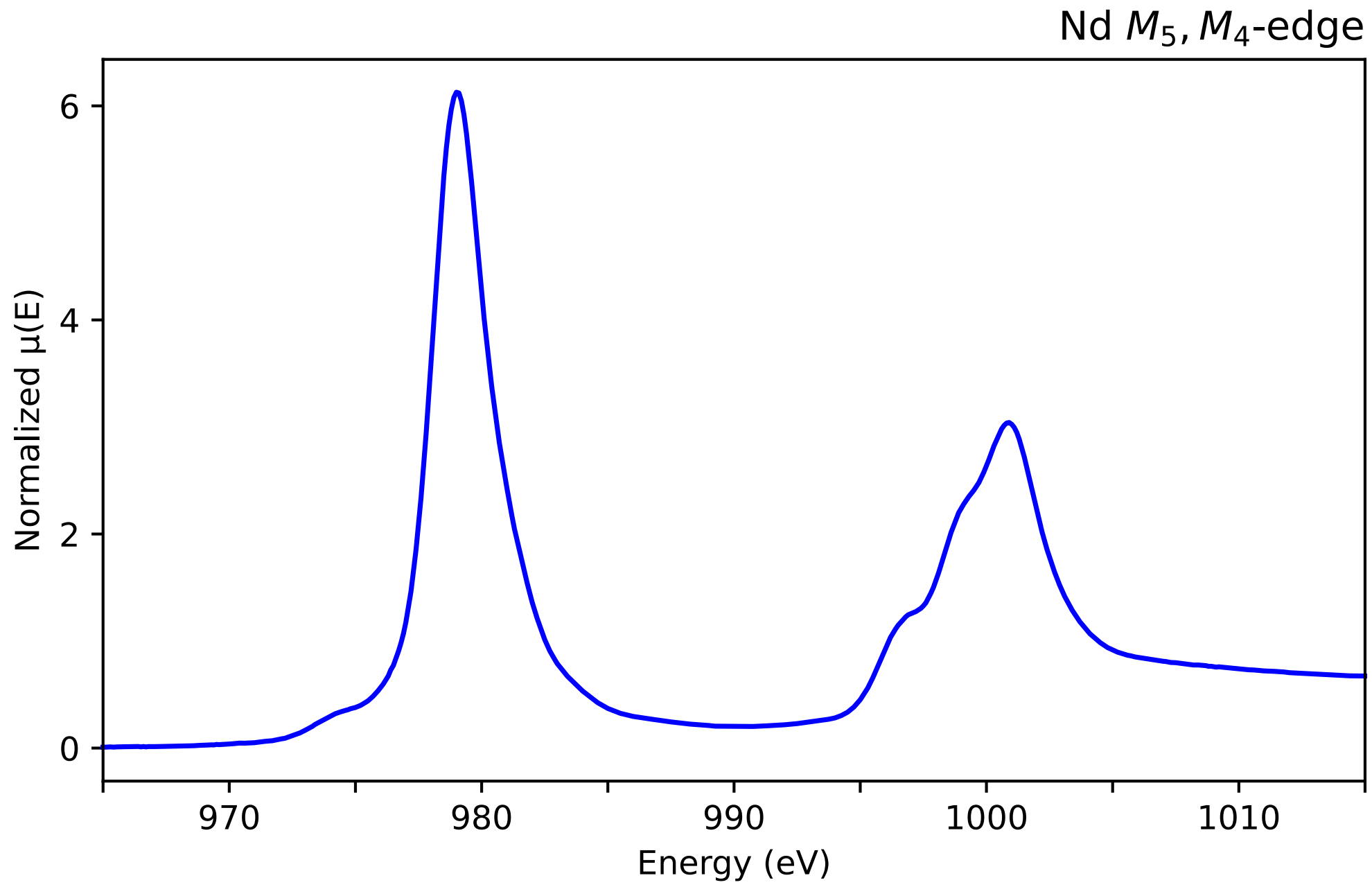

Figure S22: The normalized X-ray absorption spectra of the Nd M_4- and M_5-edge for 12R-BNM-S2 collected at the Stanford Synchrotron Radiation Lightsource Beamline 8-2. A reference comparison for the Nd^{3+} state at this edge is not readily available. We note that the features on the M_4 edge ($\sim 1000\,\text{eV}$) appear smeared and poorly resolved compared with prior studies on single-valent Nd,[14–16] supporting our interpretation of mixed-valence Nd.

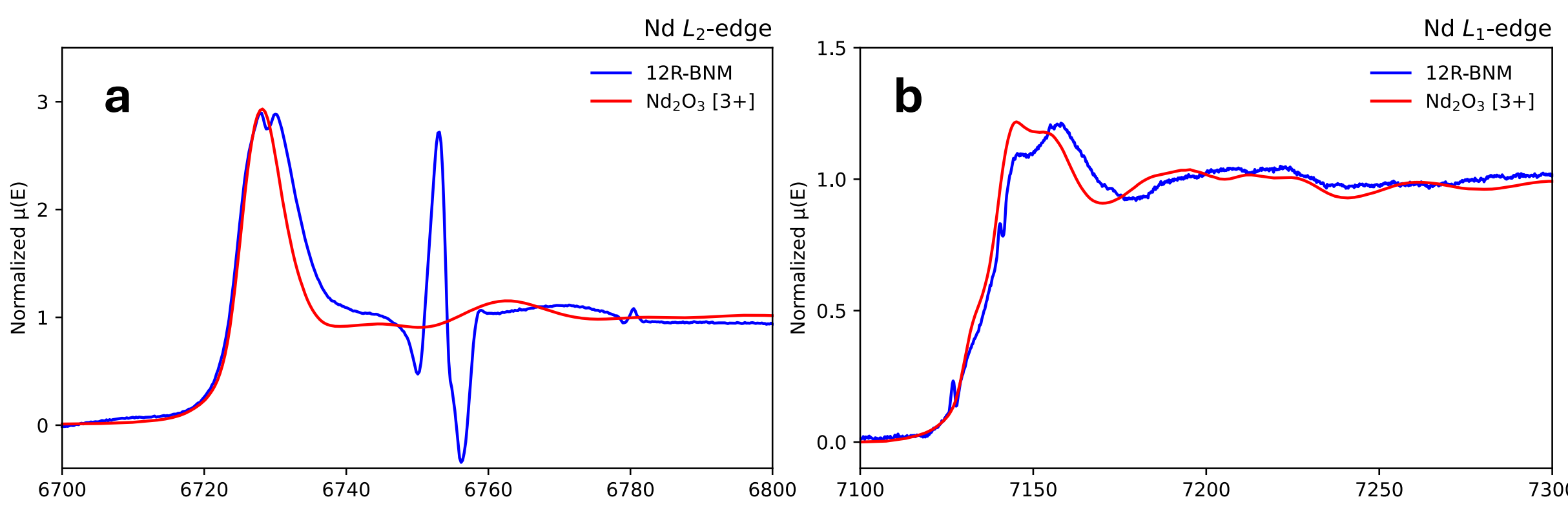

Figure S23: The normalized X-ray absorption energy of the Nd (a) L_2 and (b) L_1-edge for 12R-BNM-S2 collected at the Stanford Synchrotron Radiation Lightsource Beamline 2-2. Data measured on our Nd_2O_3 sample [Figure S33] is presented alongside 12R-BNM. However, secondary Bragg scattering within the Si monochromator contributed unwanted features in the data here.

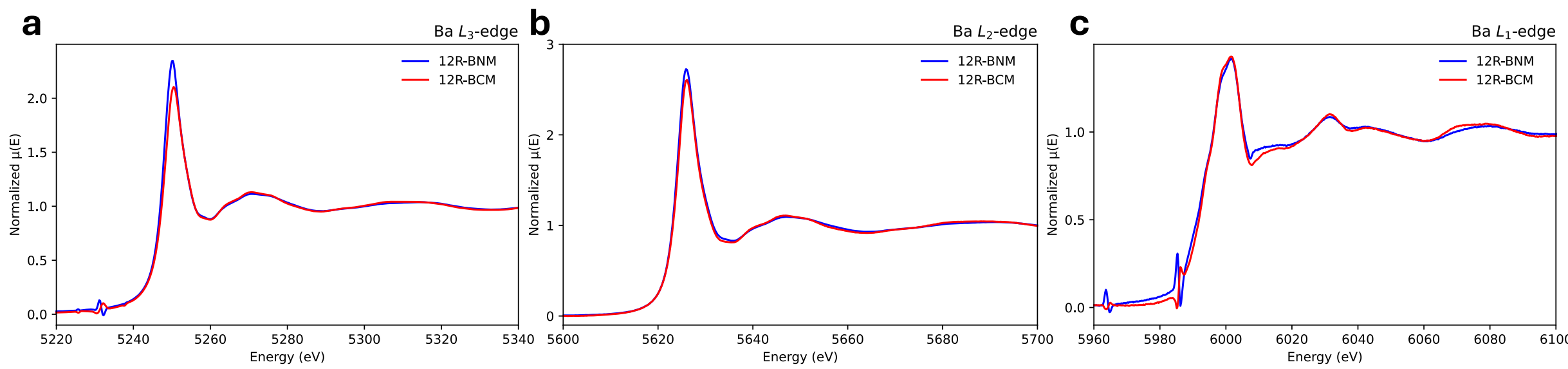

Figure S24: Normalized XAS spectra of the Ba (a) L_3, (b) L_2, and (c) L_1-edges for both 12R-BNM-S2 and 12R-BCM, collected at the Stanford Synchrotron Radiation Lightsource Beamline 2-2.

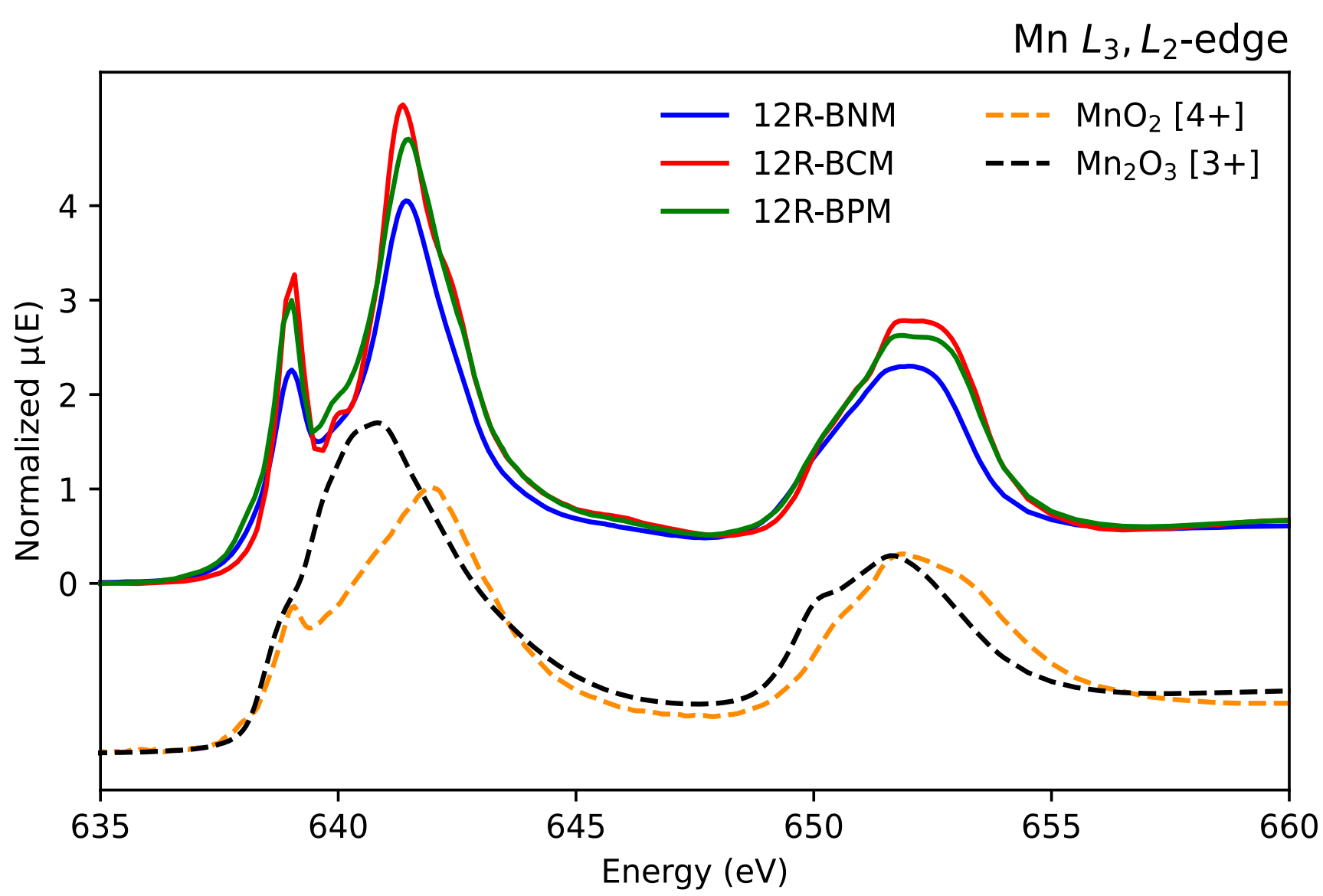

Figure S25: Mn L-edge spectra for 12R-BCM, 12R-BPM, and 12R-BNM with an MnO_2 standard. The data for 12R-BPM and 12R-BCM were previously published by Regier *et al.*[2] Data are normalised, but not area-normalised. Corresponding spectra with area normalisation are shown in Figure 2(c).

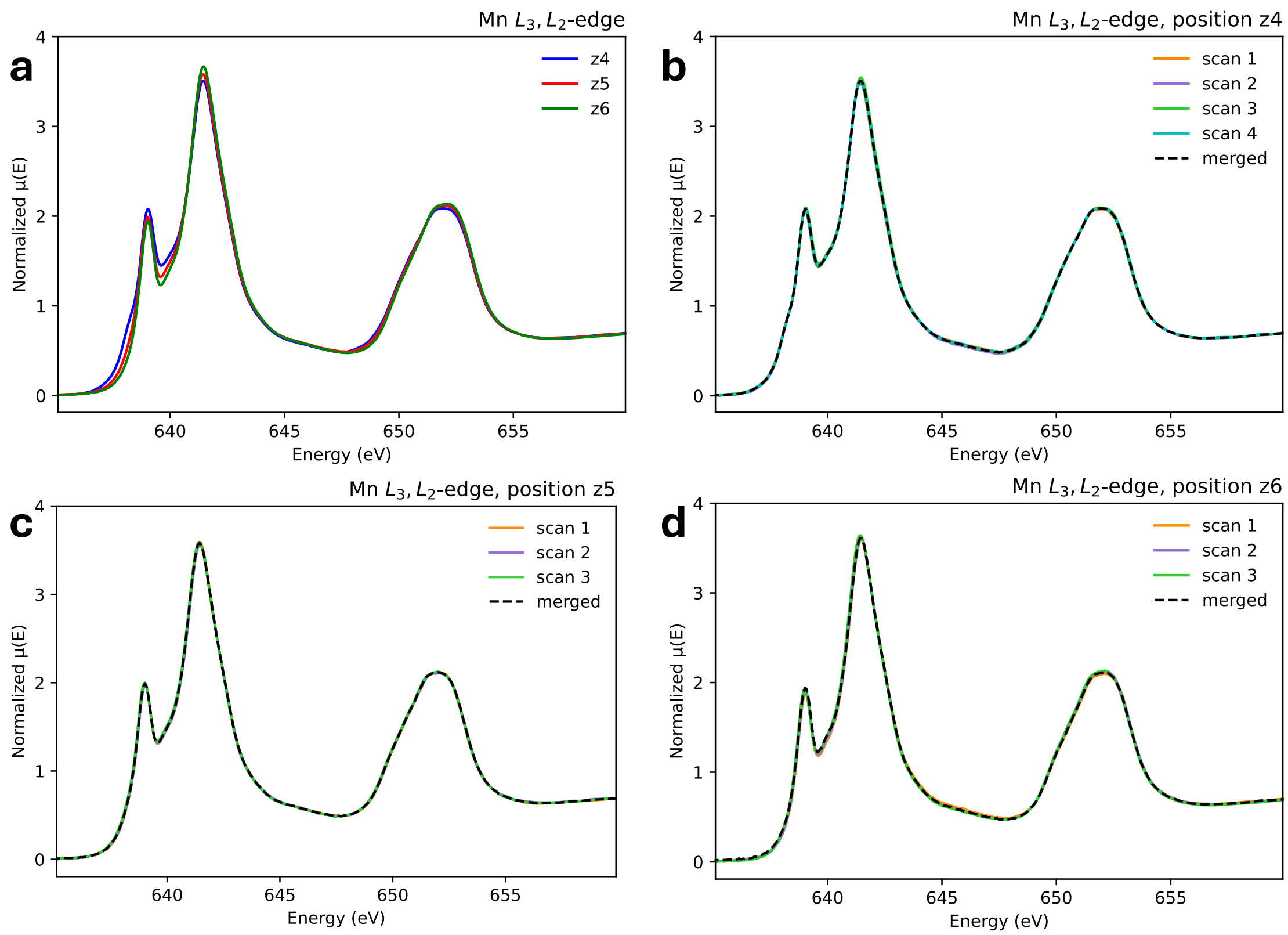

Figure S26: Normalized 12R-BNM-S2 XAS spectra at the Mn L-edge obtained from the Stanford Synchrotron Radiation Lightsource Beamline 8-2. (a) presents the merged raw data from positions z4 (b), z5 (c), and z6 (d), where z4, z5, and z6 are positions on the sample-holder spaced 1 mm apart. Noticeable variation was observed between measurement positions, each horizontally offset 1 mm in the sample holder. z5 had data between that of z4 and z6, and was chosen as representative for comparison to 12R-BCM and 12R-BPM [Figure 2(c)]. The change in pre-peak shape in z4 suggests a higher proportion of the reduced Mn^{3+} state in this measurement.

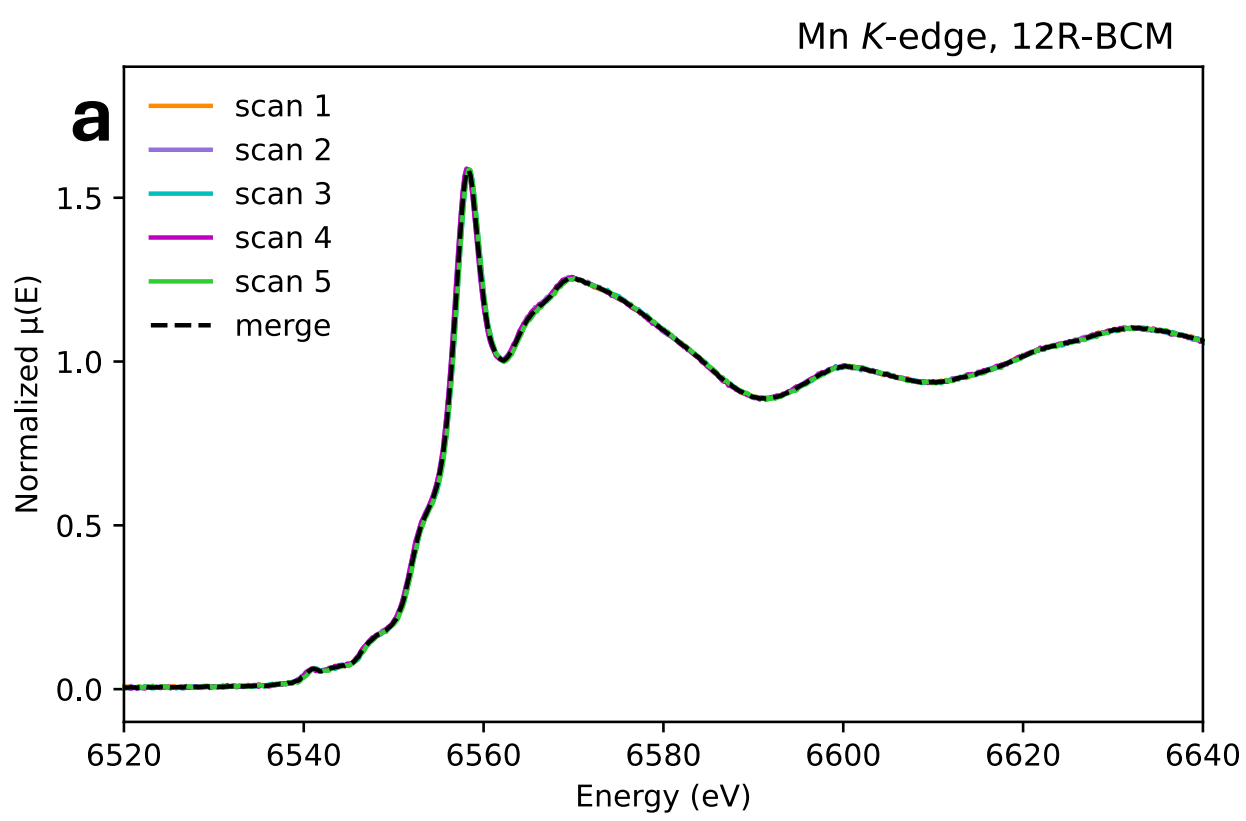

Figure S27: Normalized raw and merged XAS spectra at the Mn *K*-edge at (a) 12R-BCM and (b) 12R-BNM-S2 collected at the Stanford Synchrotron Radiation Lightsource Beamline 2-2. No appreciable differences between scans were observed. The resulting merged spectra are compared in Figure 2(a) with area normalisation and Figure S28 without area normalisation.

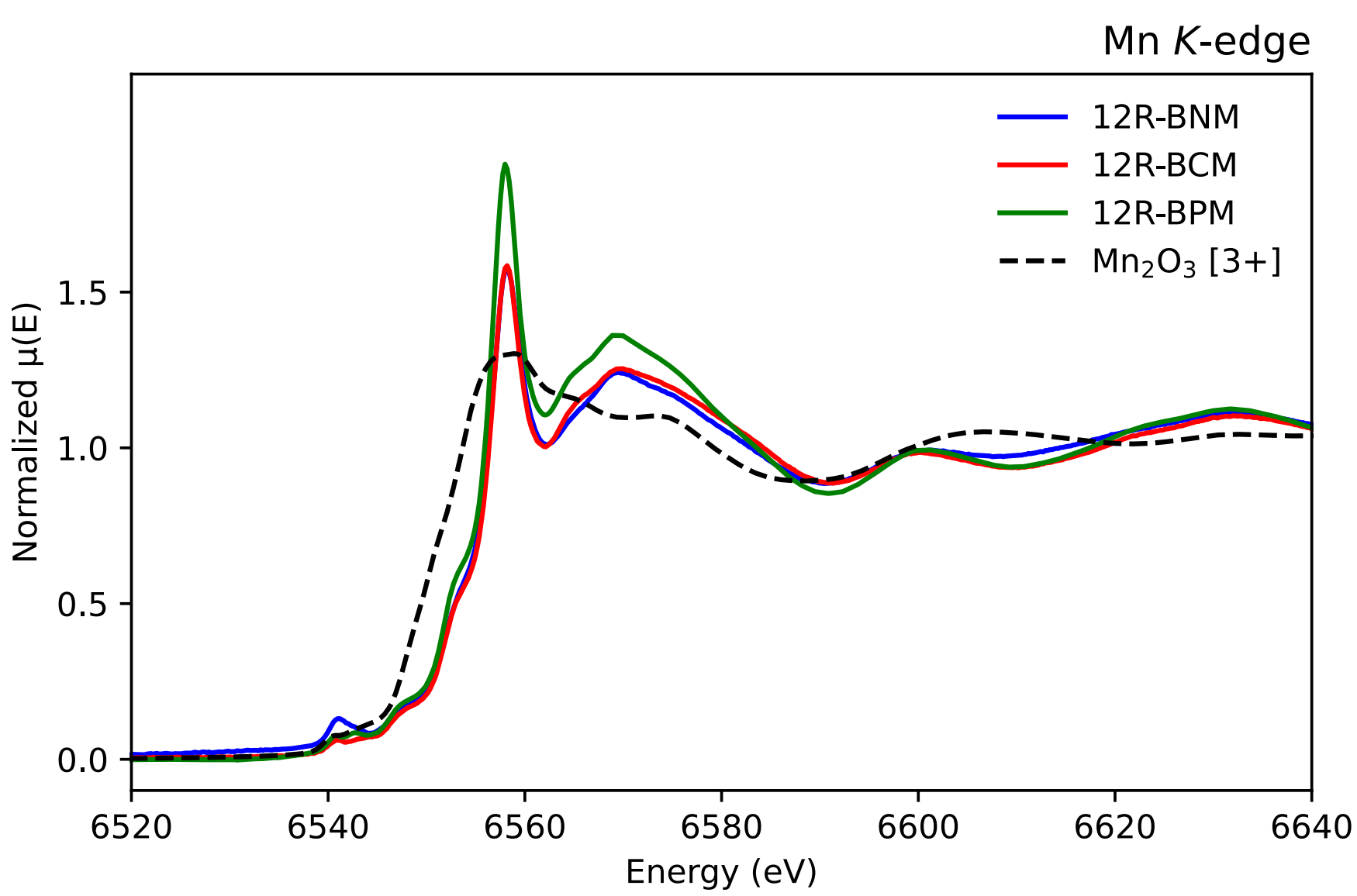

Figure S28: Mn *K*-edge spectra for 12R-BCM, 12R-BPM, and 12R-BNM with an MnO_2 standard. The data for 12R-BPM and 12R-BCM were previously published by Regier *et al.*[2] Data are normalised, but not area-normalised. Corresponding spectra with area normalisation are shown in Figure 2(a).

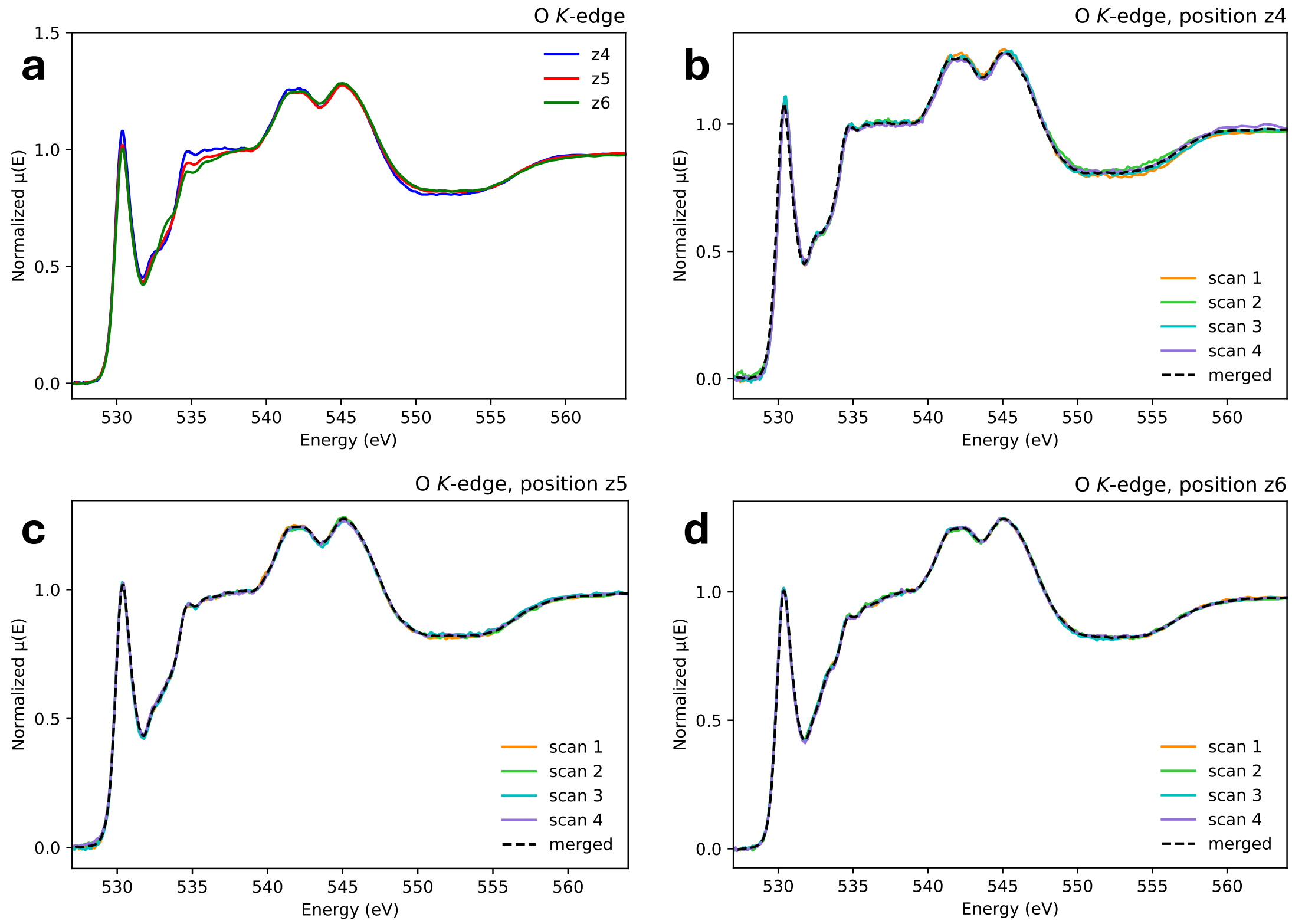

Figure S29: Normalized 12R-BNM-S2 XAS spectra at the O K-edge obtained from the Stanford Synchrotron Radiation Lightsource Beamline 8-2. (a) presents the merged raw data from positions z4 (b), z5 (c), and z6 (d), where z4, z5, and z6 are positions on the sample-holder spaced 1 mm apart. Because noticeable variation was again observed between measurement positions, z5, with spectrum between that of z4 and z6, was chosen as representative for comparison to 12R-BCM and 12R-BPM [Figure 2(d)].

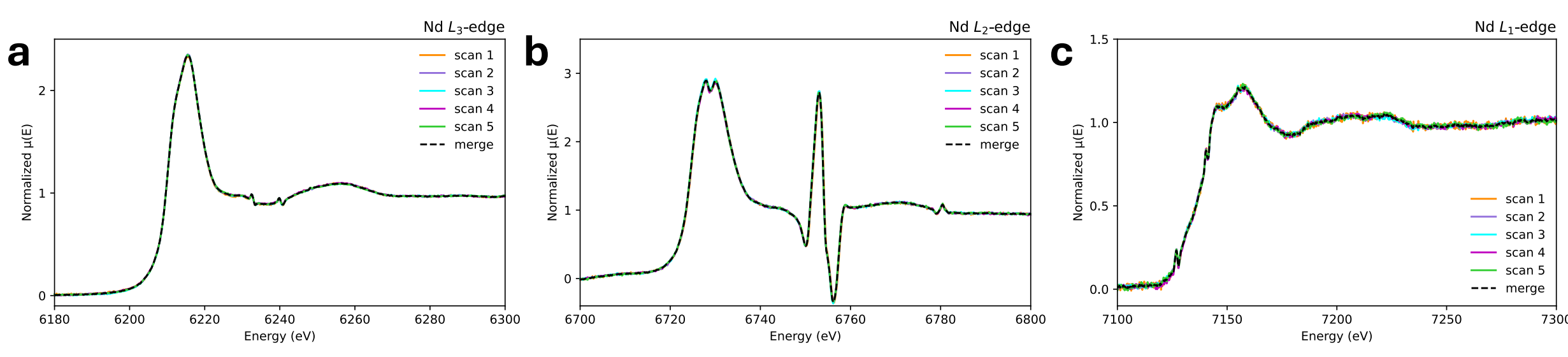

Figure S30: The normalized raw and merged X-ray absorption energy of the Nd (a) L_3-, (b) L_2- and (c) L_1-edge for 12R-BNM-S2 collected at the Stanford Synchrotron Radiation Lightsource Beamline 2-2.

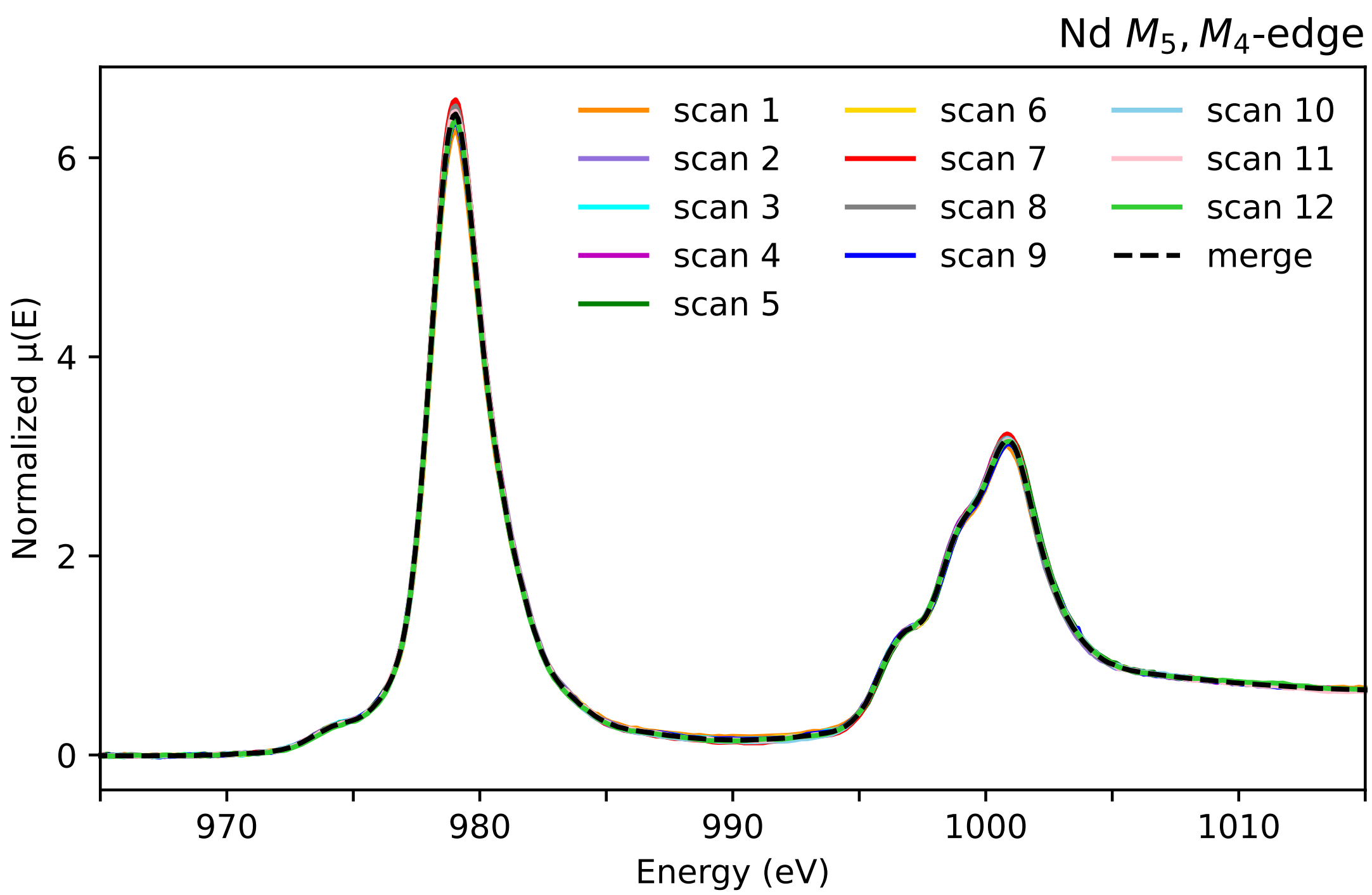

Figure S31: The normalized raw and merged X-ray absorption spectra of the Nd M_4 and M_5-edge for 12R-BNM-S2 collected at the Stanford Synchrotron Radiation Lightsource Beamline 8-2.

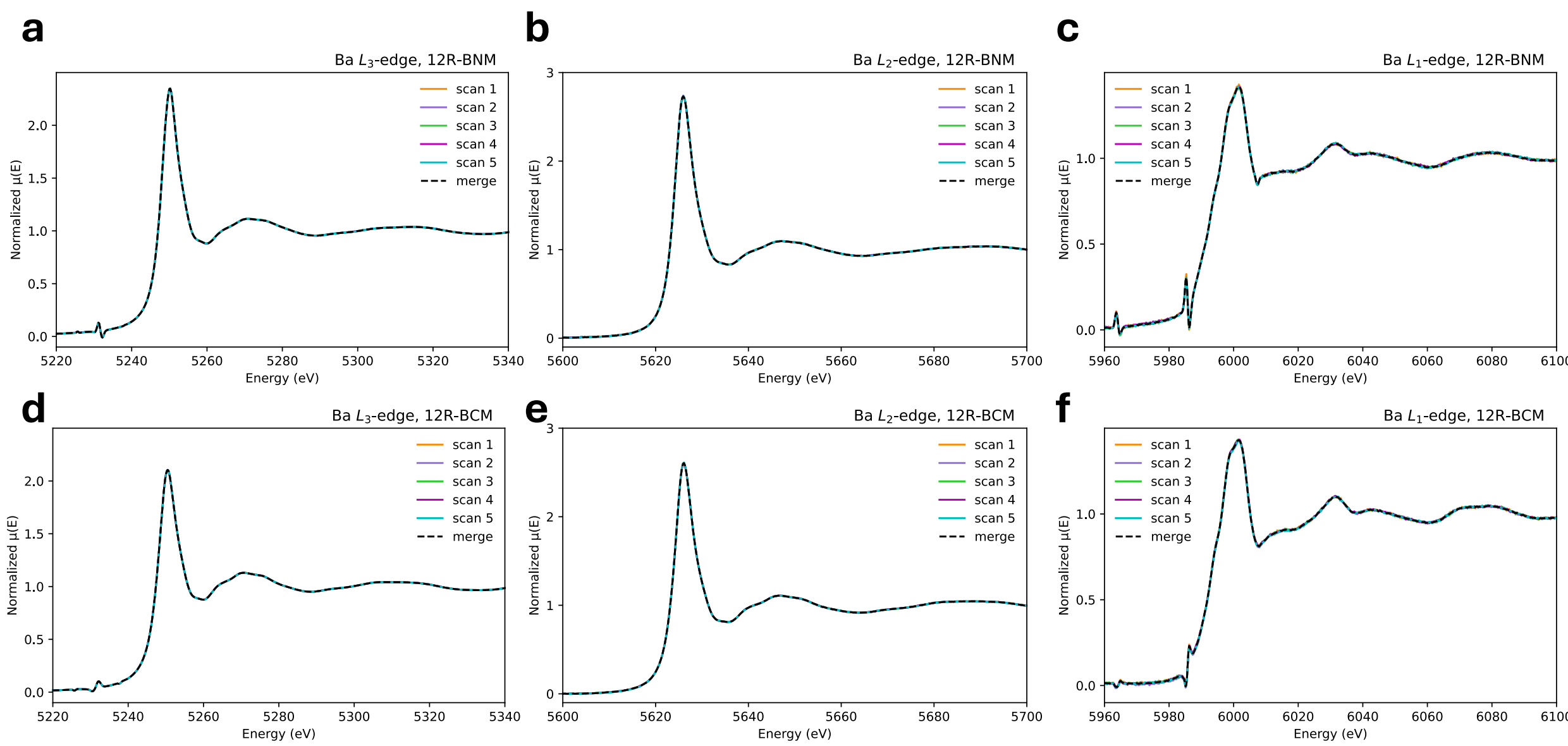

Figure S32: Normalized raw and merged XAS spectra at the Ba L_3-, L_2-, and L_1-edges for (a, b, c) 12R-BNM-S2 and (d, e, f) 12R-BCM collected at the Stanford Synchrotron Radiation Lightsource Beamline 2-2. No appreciable differences between scans were observed. The resulting merged plots are compared directly in Figure S24.

S8 X-ray diffraction on neodymium (III) oxide standard

In the XAS data on the Nd edges, we use Nd_2O_3 as the standard. Figure S33 shows a diffraction pattern measured on beamline 2-1 at SSRL, with associated Rietveld refinement, for the Nd_2O_3 standard used. Diamond was used as a diluent due to the highly absorbing nature of neodymium.

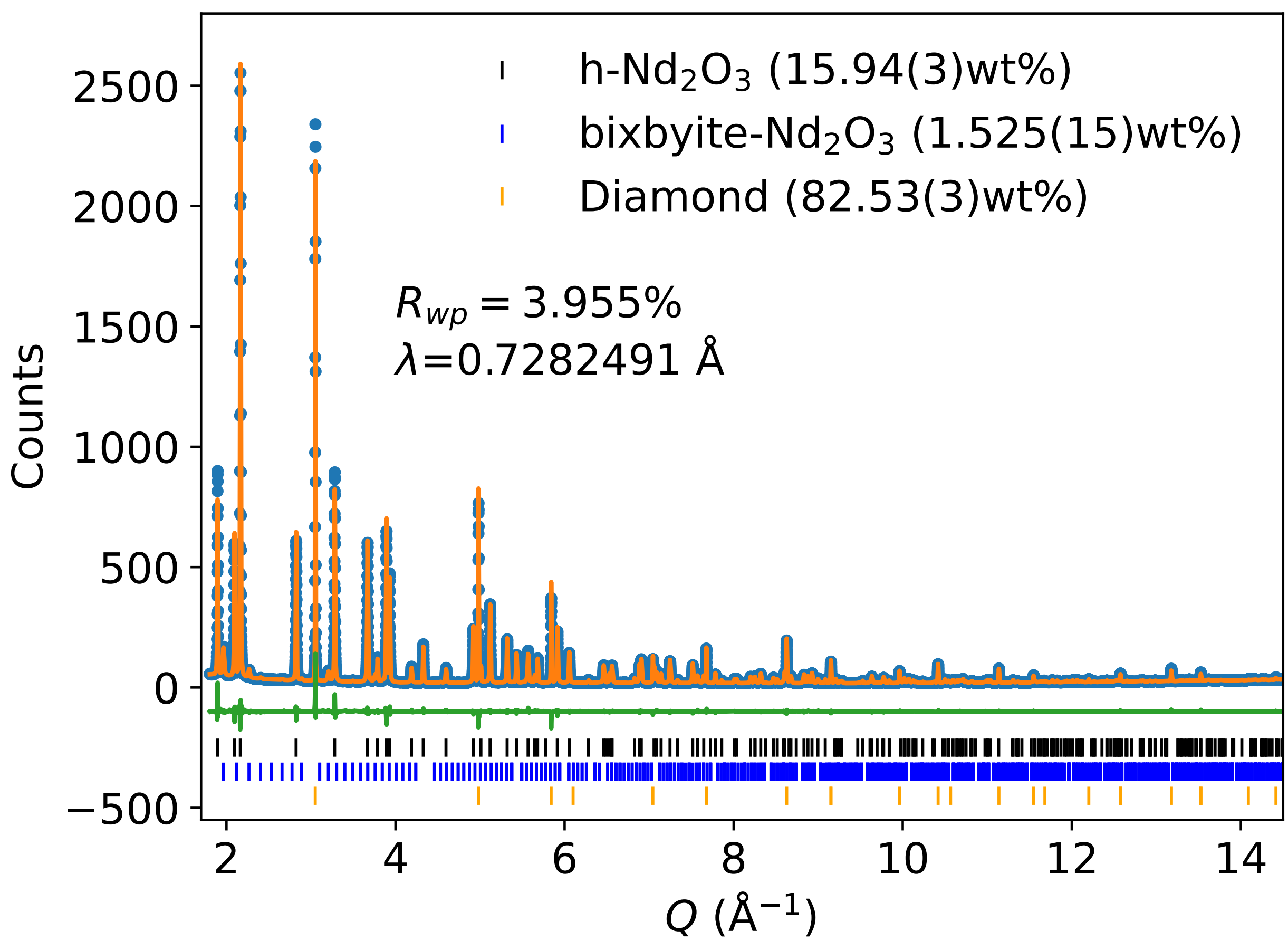

Figure S33: Diffraction data from beamline 2-1 on the Nd_2O_3 sample used as an Nd^{3+} standard in the X-ray absorption spectroscopy measurements presented in Figure 2(b), S23, and S22. Rietveld refinement was performed using Topas-Academic version 7. Two polymorphs of Nd_2O_3 are present: hexagonal Nd_2O_3 (15.94wt%) and bixbyite Nd_2O_3 (1.52wt%). The remaining material is a diamond diluent (82.53wt%) included due to the highly absorbing nature of neodymium.

S9 Curie-Weiss fitting to magnetometry data

The Curie-Weiss equation is typically used to fit magnetometry data if inverse susceptibility has a linear temperature dependence:

$$\chi^{-1}(T) = \frac{T - T_{\mathrm{CW}}}{C} \tag{S2}$$

Inverse susceptibility data, Figure S34, can be fit with a modified Curie-Weiss equation when there is non-linearity at high-temperatures. The modified Curie-Weiss equation is given as Equation 1 in the main text, and reproduced here for ease of access:

$$\chi^{-1}(T) = \frac{T - T_{\mathrm{CW}}}{C + \chi_0(T - T_{\mathrm{CW}})} \tag{S3}$$

When fitting the modified Curie-Weiss equation to inverse susceptibility data which has a temperature-independent component χ_0, the results of the fit are highly sensitive to the range of temperatures over which the fit is performed. To account for this, we have performed a fit of the modified Curie-Weiss equation to the susceptibility data between a range of temperatures T_{min} and T_{end}, where $T_{\mathrm{end}} \approx 400\,\mathrm{K}$ and T_{min} is varied. The result of this fitting is shown in Figure S35.

The values seem to plateau with $T_{\mathrm{min}} = 270\,\mathrm{K}$, below which the data deviates from modified Curie-Weiss equation. On the basis of this plot we therefore select this value for T_{min}, which gives fitted values as presented in Table 1 with comparisons to the Pr and Ce-containing analogs to 12R-BNM. The fit is shown in Figure S36.

We note that in this calculation we assume zero O vacancies in the molar mass calculation of 12R-BNM for simplicity.

To evaluate the influence of the $Ba_4Mn_3O_{10}$ impurity phase, we extracted the $Ba_4Mn_3O_{10}$ susceptibility data reported in Figure 2 of Zubkov *et al.*[12] using data extraction software and compared linear fits to the high-temperature data with and without subtraction of this contribution. We do not know from Ref[12] the errors in their data, so we do not attempt

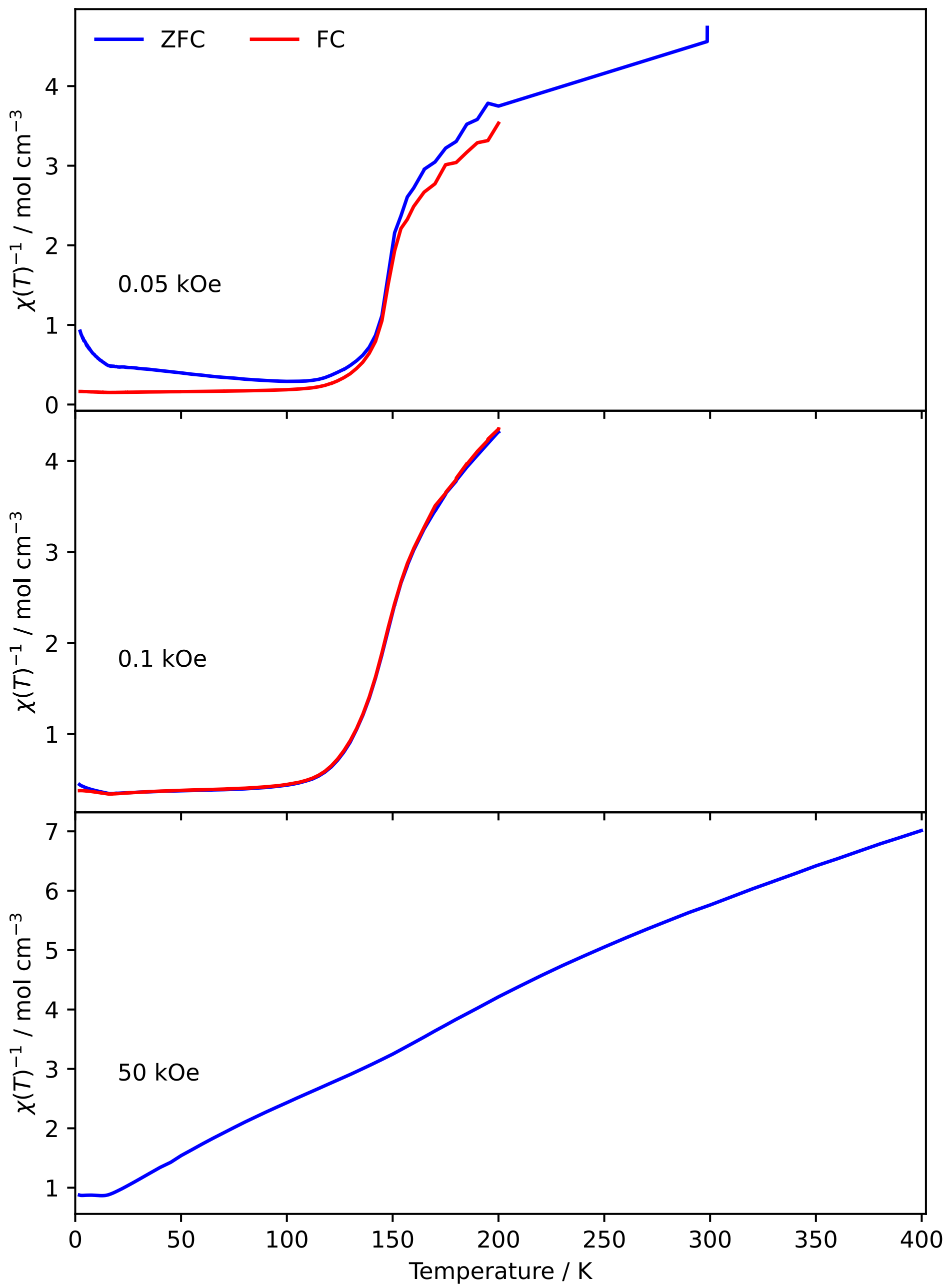

Figure S34: Inverse DC magnetic susceptibility for 12R-BNM-S2, as measured during heating. For FC measurements, the field applied on cooling before heating matches the field applied during measurement on heating.

to propagate errors in this analysis. As shown in Figure S37, the near-identical gradients of these high-temperature fits show that the impurity contribution does not meaningfully impact the calculated Bohr magneton per formula unit.

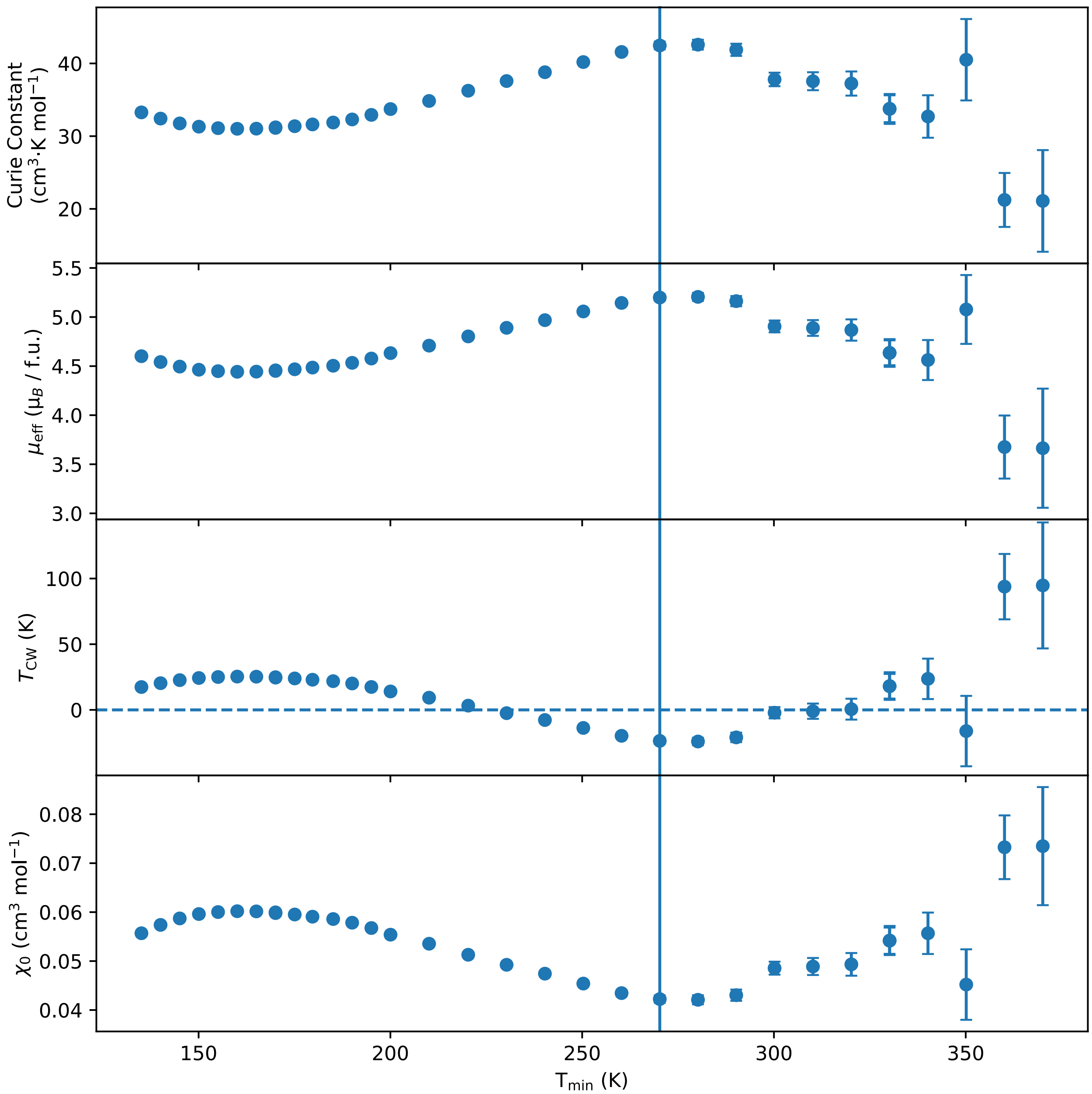

Figure S35: Results of a fit of the modified Curie-Weiss equation (Equation 1) to the susceptibility data (measured on heating in a field of 50 kOe after zero-field-cooling) between a range of temperatures T_{min} and T_{end}, where $T_{\mathrm{end}} \approx 400\,\mathrm{K}$, as a function of T_{min}, for 12R-BNM-S2. The vertical line indicates the temperature we selected for the fit presented in the main text.

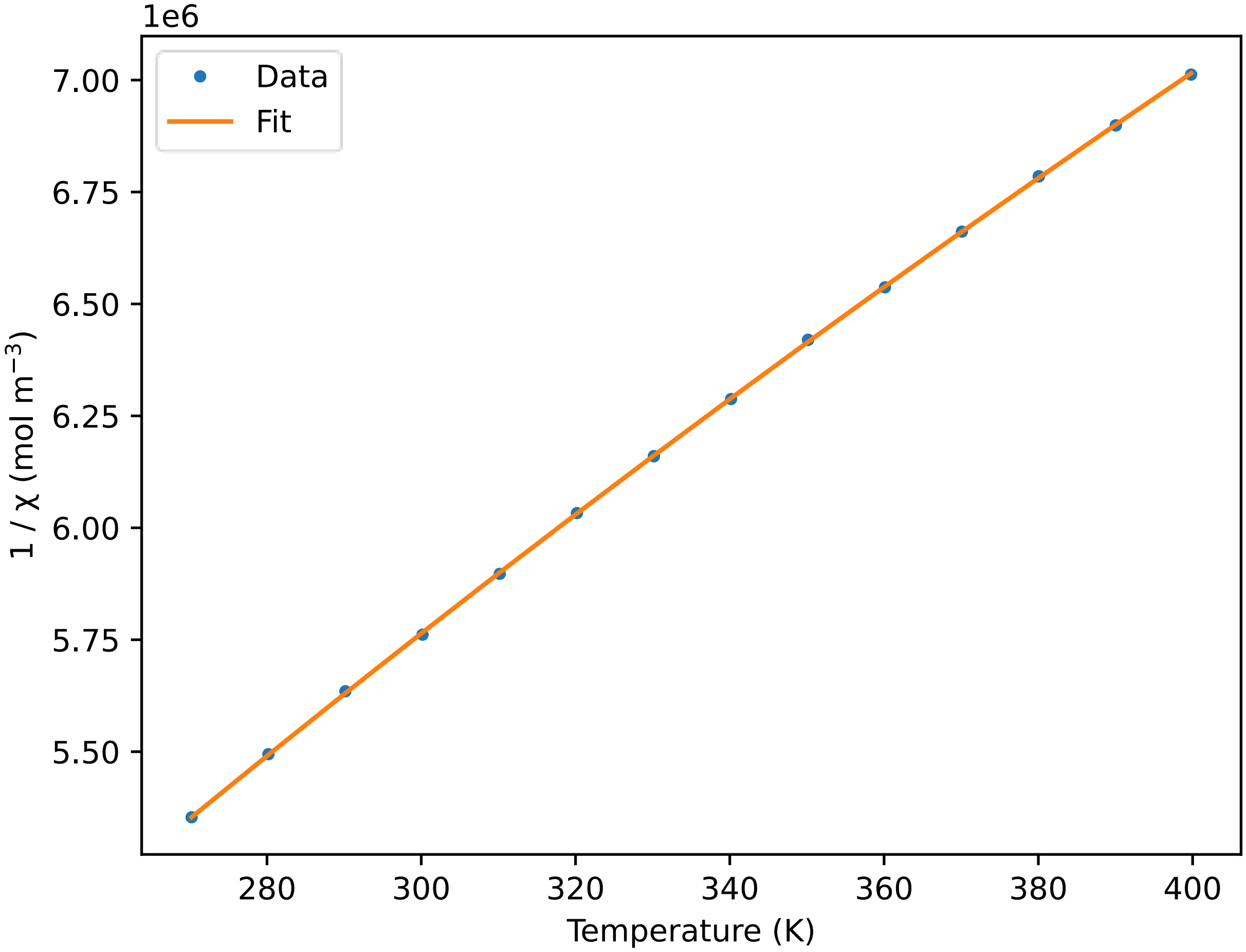

Figure S36: A fit of the modified Curie-Weiss equation (Equation 1) to the inverse susceptibility data on 12R-BNM-S2, measured in an external field of 50 kOe after cooling in zero-field.

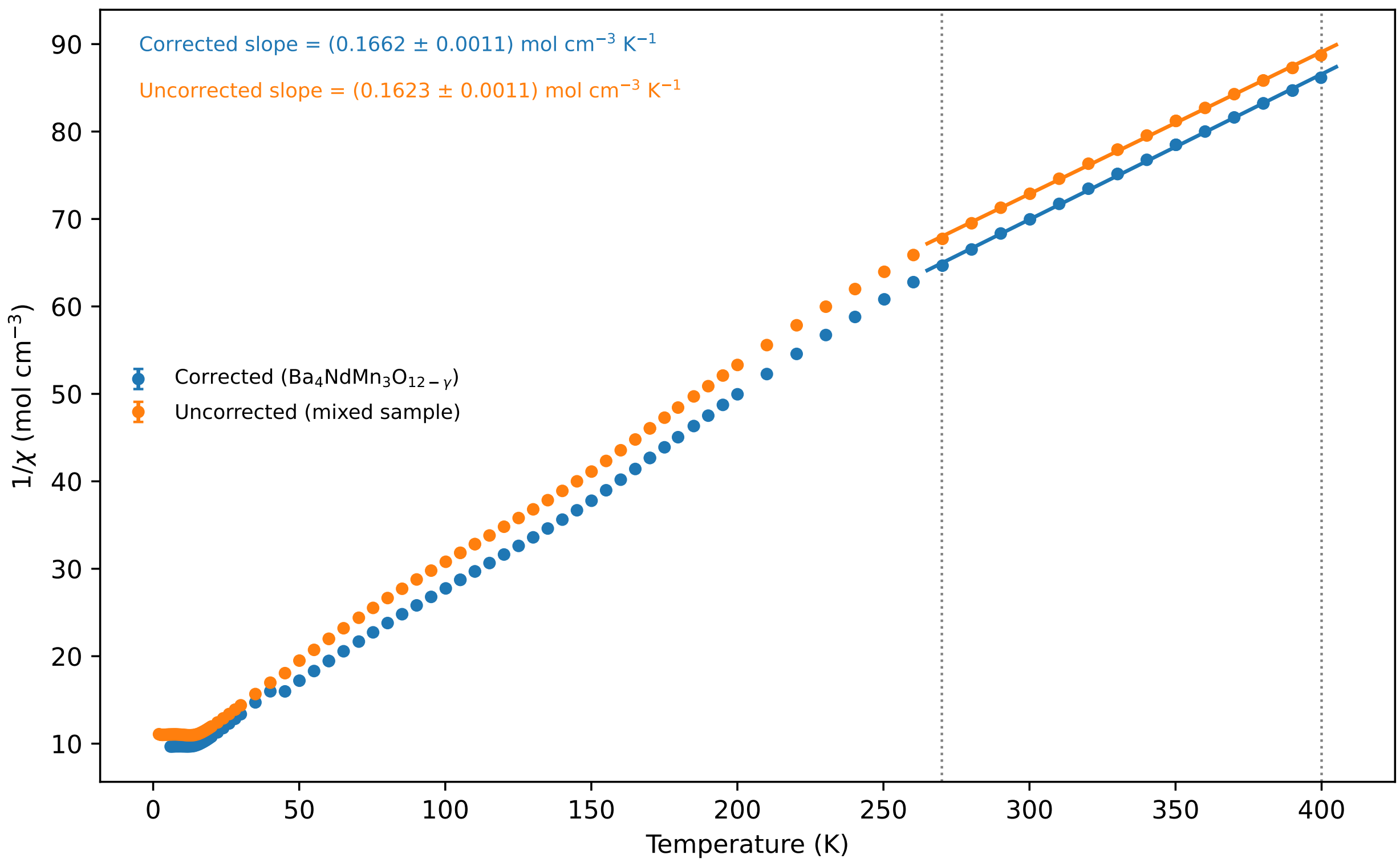

Figure S37: Comparison of inverse susceptibility and a linear fit to the high-temperature data for 12R-BNM-S2 with and without correction for the $Ba_4Mn_3O_{10}$ impurity phase. The $Ba_4Mn_3O_{10}$ susceptibility contribution was estimated from data extracted from Figure 2 of Zubkov *et al.*[12] The error in gradients use the weight percent error from Rietveld analysis and assume a sample mass error of 0.1 mg; error in the $Ba_4Mn_3O_{10}$ data is not included and so errors are likely an underestimate. The near-identical gradients of the high-temperature fits show that the impurity contribution does not meaningfully affect the high-T gradient, and hence the calculated Bohr magneton per formula unit.

S10 Expected magnetic moment in the paramagnetic regime

In this section, we calculate the expected magnetic moment per formula unit in the paramagnetic regime for 12R-$Ba_4LnMn_3O_{12-\gamma}$ (Ln=Ce,Pr,Nd).

The spin-only magnetic moment of an atom, ignoring orbital contributions, is given by:

$$\mu_{\text{eff}} = g\sqrt{S(S+1)} \tag{S4}$$

where g is the Landé g-factor for the spin-only case and S is the spin of the unpaired electrons on the atom. S is 0 for Ce^{4+}, Ba^{2+}, and O^{2-}, 1/2 for Pr^{4+}, 1 for Nd^{4+}, and $\frac{3}{2}$ for Mn^{4+} and Nd^{3+}. To calculate the expected magnetic moment per formula unit which we gain from fitting the Curie-Weiss law to susceptibility data in the paramagnetic regime, we sum the moments in quadrature, i.e.:

$$\mu_{\text{total}} = \sqrt{\sum_i \mu_i^2} \tag{S5}$$

For the spin-only case (i.e. assuming no orbital contributions to magnetism) we can assume $g = 2$. However, for f orbitals in the lanthanoid cations, we must account for the orbital effects by using a different value of the Landé g-factor calculated as follows:

$$g_J = 1 + \frac{J(J+1) + S(S+1) - L(L+1)}{2J(J+1)} \tag{S6}$$

where S is the total spin angular momentum quantum number, L is the total orbital angular momentum quantum number, and J is the total angular momentum quantum number ($\vec{J} = \vec{L} + \vec{S}$). In turn we then calculate the effective moment using J as follows:

$$\mu_{\text{eff}} = g_J\sqrt{J(J+1)} \tag{S7}$$

To calculate g_J we assume maximum spin S and orbital angular momentum L. For Pr^{4+}, this corresponds to $S = \frac{1}{2}$ and $L = 3$, which is the case for a single f electron. Nd^{4+} has two f electrons, which are maximized by assuming occupation of different f orbitals to give parallel spin, resulting in $S = 1$, and the highest M_L total orbital occupancy occurs when the two electrons occupy $m_L = 3$ and $m_L = 2$ states, resulting in $L = 3 + 2 = 5$. We then use $J = |L - S|$, giving $J = \frac{5}{2}$, 4, and $\frac{9}{2}$ for Pr^{4+}, Nd^{4+}, and Nd^{3+} respectively.

These equations can then be used to calculate a spin-only effective moment and an effective moment including orbital effects. The results of these calculations are shown in Table S5 for 12R-$Ba_4CeMn_3O_{12}$, 12R-$Ba_4PrMn_3O_{12}$, and 12R-$Ba_4NdMn_3O_{12}$ various configurations. Note that in the calculations in Table S5, we assume all moments behave independently of one another; i.e. that there is no magnetic clustering effect.

Table S5: Expected effective magnetic moments for $Ba_4\mathit{Ln}Mn_3O_{12}$ compounds. The spin-only case assumes $g = 2$ and spin-only contributions from Mn and *Ln*, whereas the "orbital included" case uses Equation S6 to calculate the Landé g-factor for the lanthanoid cation. In this table, it is assumed that all moments behave independently of one another; i.e. that there is no magnetic clustering effect.

Compound	Mag ions	Predicted μ_{eff} (μ_B / f.u.)	
		Spin-only	Orbital included
12R-$Ba_4CeMn_3O_{12}$	3×Mn^{4+}	∼6.71	
12R-$Ba_4PrMn_3O_{12}$	3×Mn^{4+}, Pr^{4+}	∼6.93	∼7.18
12R-$Ba_4NdMn_3O_{12}$	3×Mn^{4+}, Nd^{4+}	∼7.28	∼7.60

Given previous work which shows a magnetic cluster effect suppressing the magnetic moment obtained by Curie-Weiss fitting,[17,18] and our own finding in this work that the magnetic moment obtained from Curie-Weiss fitting (5.24(3) μ_B per formula unit) is less than that predicted in Table S5, we may alternatively wish to calculate the effective magnetic moment considering the Mn_3O_{12} trimers as single units. For the case where all three Mn cations are in the Mn^{4+} oxidation state, we can model 12R-BNM as having magnetic contributions from the Nd^{4+} cation and an $S_{\text{eff}} = \frac{3}{2}$ magnetic cluster, obtaining μ_{eff} per formula unit using Equation S7 to calculate the moment in the lanthanoid, Equation S4 to calculate the moment in the cluster, and Equation S5 to obtain the total moment. The result of this

calculation is shown in Table S6.

An alternative possibility worthy of consideration is that the Nd cation becomes Nd^{3+} and one of the Mn cations in the cluster is Mn^{5+}. In this case, Nd has three $4f$ electrons and one of the three Mn cations becomes d^2. Here we assume that within the clusters the two Mn^{4+} cations interact antiferromagnetically, resulting in $S_{\text{eff}} = 1$. Table S6 compares the calculated moments assuming the absence of magnetic clusters (i.e. free spins), and the two possible magnetic clustering arrangements, for both the spin-only calculation and the calculation including orbital contributions (Equation S6) for the Nd cation.

Table S6: Expected effective magnetic moments for 12R-$Ba_4NdMn_3O_{12}$ under various assumptions. In the spin-only case, Equation S4 is used to calculate the Nd moment, whereas in the "orbital-included" case, Equation S7 is used to calculate the Nd moment. The four cases we then consider for the Mn_3O_{12} trimers are: (1) the free-spin case with no interactions between Mn^{4+} cations in the paramagnetic regime and Nd^{4+}, (2) the magnetic cluster case with Nd^{4+} and clusters with $S_{\text{eff}} = \frac{3}{2}$, (3) the free-spin case with no interactions between Mn^{4+}/Mn^{5+} cations in the paramagnetic regime and Nd^{3+}, and (4) the magnetic cluster case with Nd^{3+} and magnetic clusters with $S_{\text{eff}} = 1$. For reference, we note that the experimentally-obtained value for 12R-BNM-S2 is $5.24(3)\,\mu_B$, which is closest to the calculated case where $S_{\text{eff}} = \frac{3}{2}$ and Nd exists in the 4+ oxidation state.

Magnetic cations	Nd^{4+}, $3\times Mn^{4+}$		Nd^{3+}, $2\times Mn^{4+}$,Mn^{5+}	
Mn interaction	Free-spin	$S_{\text{eff}} = \frac{3}{2}$ clusters	Free-spin	$S_{\text{eff}} = 1$ clusters
Spin-only	$\sim$7.28 μ_B	$\sim$4.79 μ_B	$\sim$7.28 μ_B	$\sim$4.79 μ_B
Orbital-included	$\sim$7.60 μ_B	$\sim$5.27 μ_B	$\sim$7.14 μ_B	$\sim$4.59 μ_B

We can see from Table S6 that the situation giving the closest moment to our experimental value, $\mu_{\text{eff}} = 5.24(3)\,\mu_B$ per formula unit, is the case where $S_{\text{eff}} = \frac{3}{2}$ and Nd exists in its 4+ oxidation state, with a value $\sim 5.27\mu_B$ per formula unit. These calculations support our claim that Nd exists as Nd^{4+} and not the far more common Nd^{3+}.

S11 Heat capacity analysis details

The full experimentally-measured heat capacity is shown in Figure S38. Typically, when analysing the magnetic contribution to heat capacity, the lattice component is obtained by fitting the data with the Debye equation[19] defined as follows:

$$C_{\text{lat}} = \frac{9nRT^3}{T_{\text{D}}^3} \int_0^{\frac{T_{\text{D}}}{T}} \frac{x^4 e^x}{(e^x - 1)^2} dx \tag{S8}$$

where T_D is the Debye temperature, n is proportional to the amount of substance, R is the ideal gas constant, T is temperature, and e is the base of the exponential function. Unfortunately, 12R-BNM does not appear to follow the behaviour predicted by the Debye equation over any temperature range above the lowest-measured temperature (which was $\sim$2 K). We show this in Figure S39(a), where we present the heat capacity data with the Debye behaviour for various chosen values of T_D showing that none match our experimental data, and Figure S39(b) which shows that heat capacity data divided by T^3 lacks a plateau against temperature indicative of Debye equation behaviour.

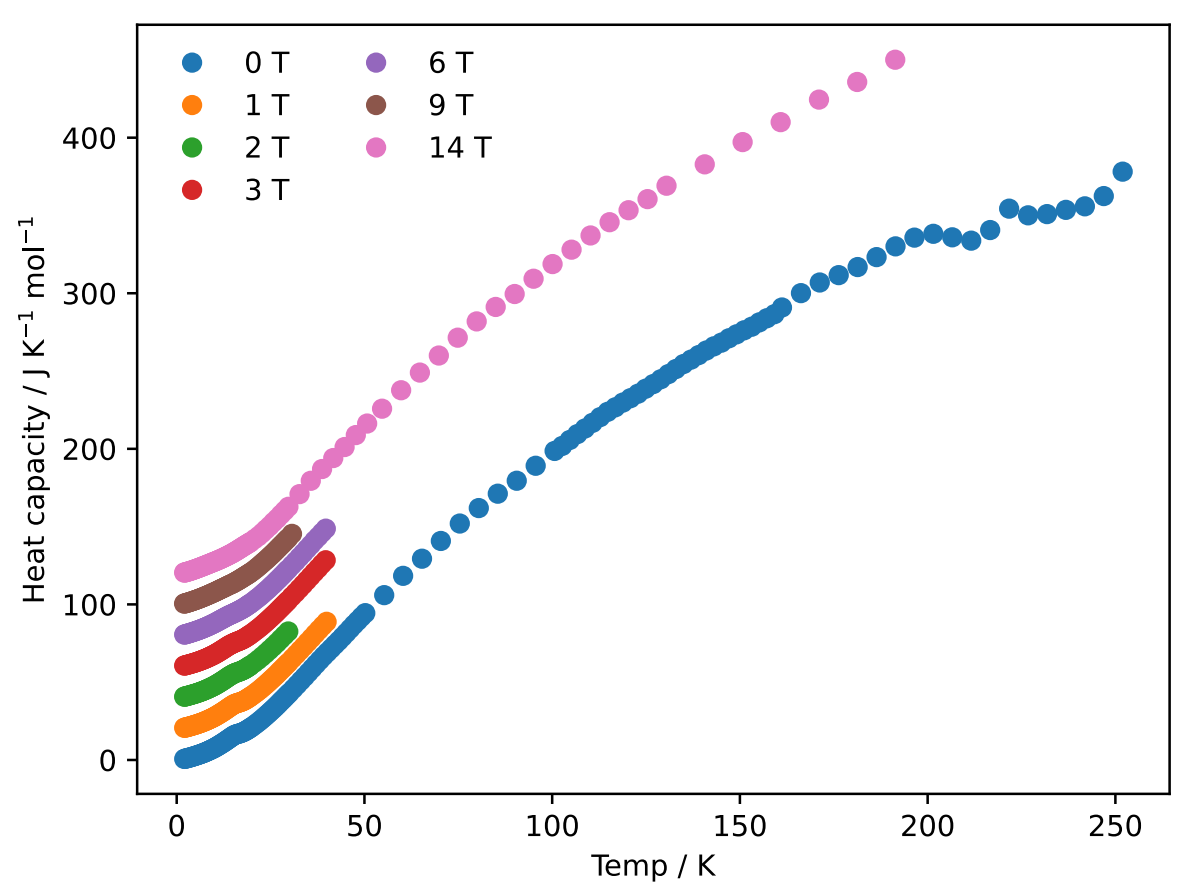

Figure S38: Experimentally-measured heat capacity datasets as a function of temperature, for various fields, for 12R-BNM-S2.

Given that we cannot use the Debye equation to, all non-magnetic contributions, C_{bkg}, to the heat capacity were subtracted using the following empirical relation:

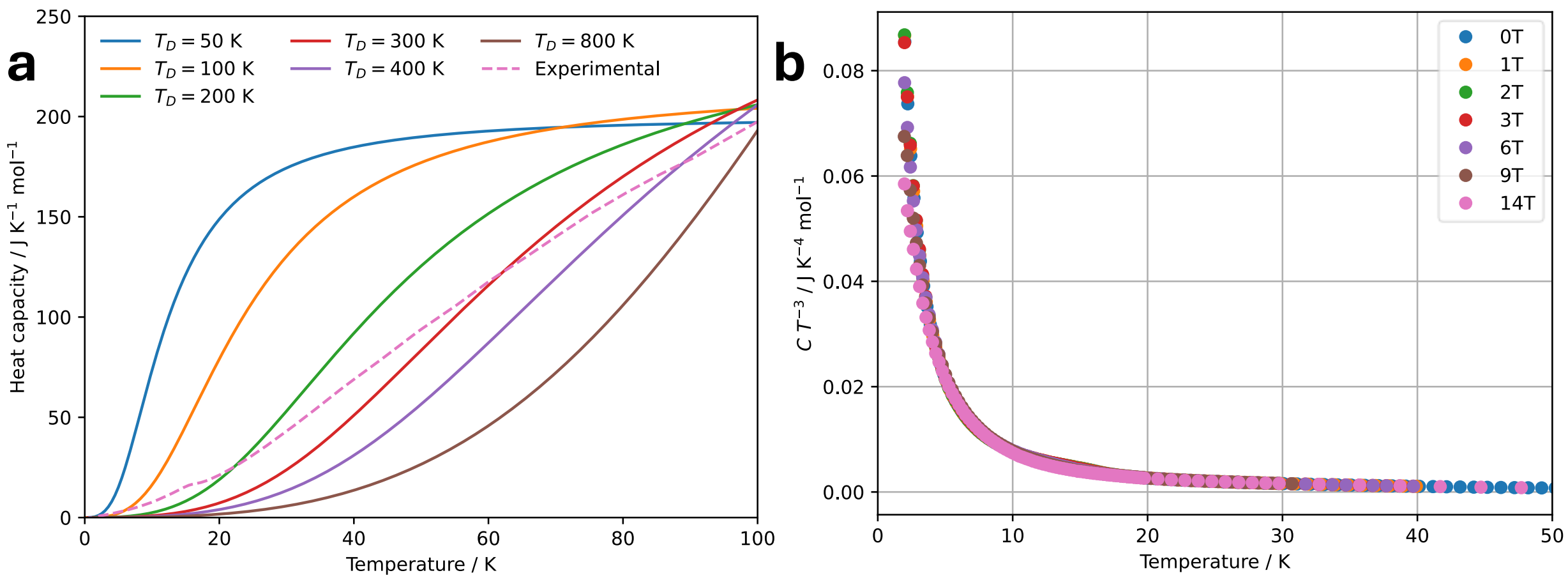

Figure S39: Evidence that the Debye Equation (Equation S8) cannot be used to calculate the lattice contribution to the total sample heat capacity. (a) shows the experimental data for 12R-BNM-S2 under a field of 0 T overlaid with various calculated Debye-type lattice heat capacities, showing a clear difference in the functional form. (b) shows the experimental data for 12R-BNM-S2 divided by T^3, showing the absence of a plateau indicative of Debye-type behaviour. In light of these findings, we opt for an alternative empirical approach to fitting the heat capacity data.

$$C_{\text{bkg}} = aT^3 + bT^2 \tag{S9}$$

where T is temperature and a and b are empirical parameters. This same empirical relation has been used previously.[1] Figure S40 shows fits of this equation to the experimental heat capacity data over the range 20 K to 40 K for 12R-BNM-S1 and 12R-BNM-S2. Figure S41 shows the total heat capacity, and the C_{bkg} subtracted, for all incident fields for 12R-BNM-S2.

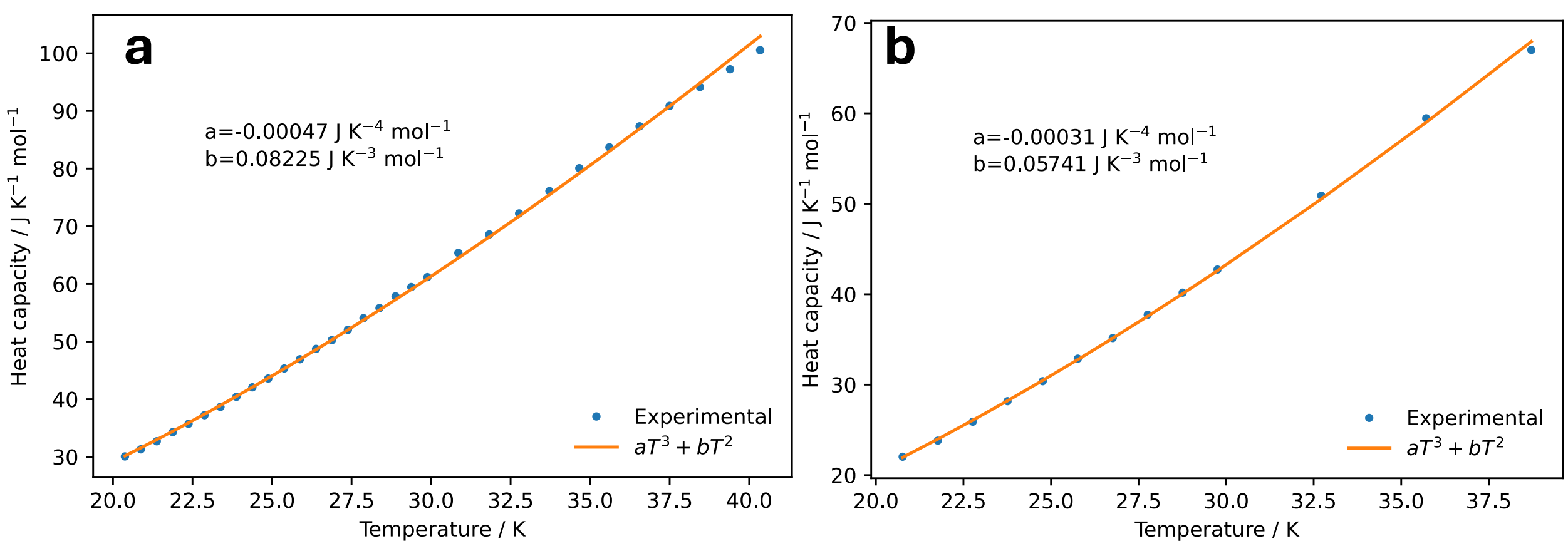

Figure S40: Fits of the empirical relation $C_{\text{bkg}} = aT^3 + bT^2$ to the temperature-dependence of heat capacity for (a) 12R-BNM-S1 and (b) 12R-BNM-S2.

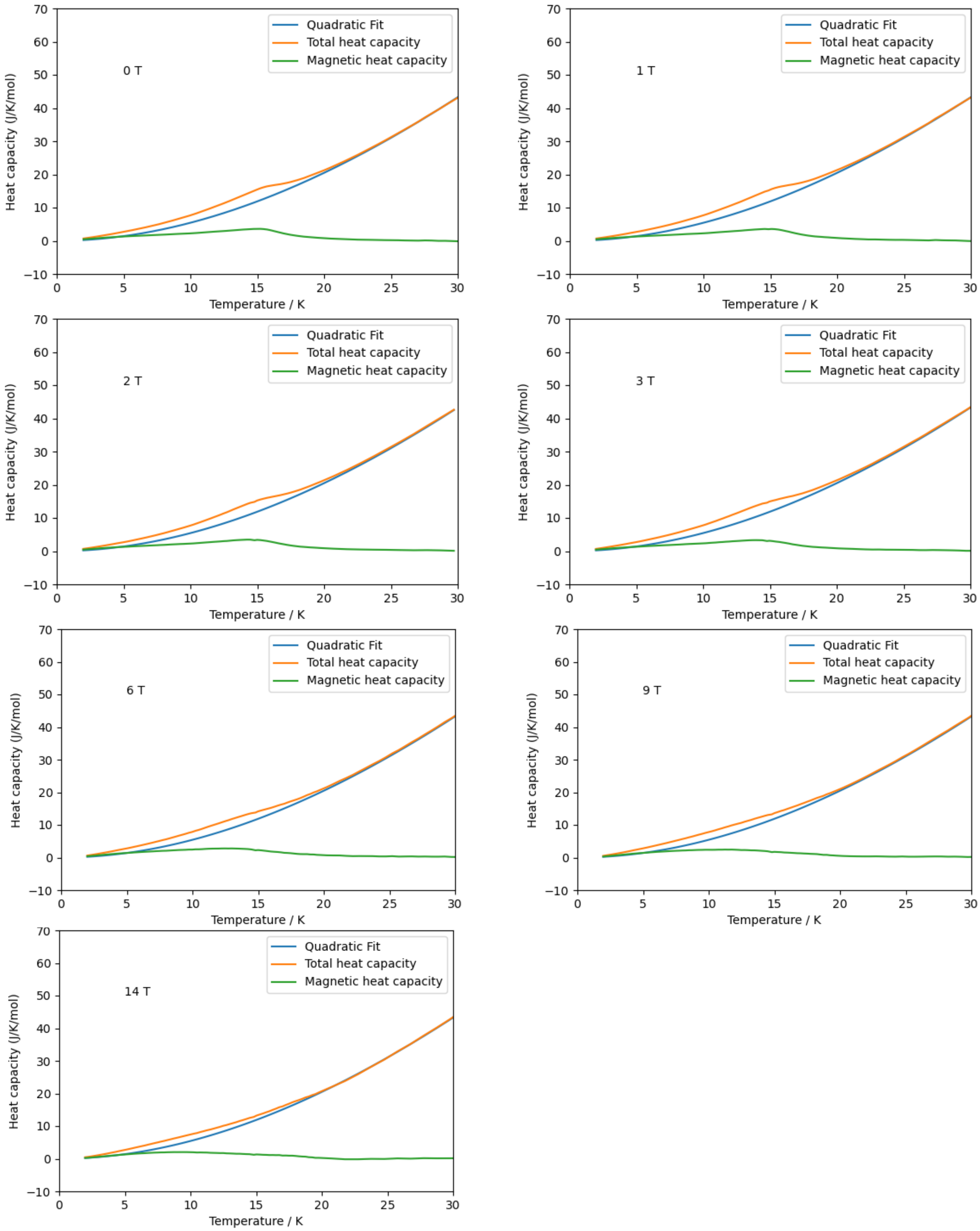

Figure S41: Comparison of the total heat capacity, background component obtained by fitting, and resulting magnetic heat capacity for 12R-BNM-S2. The subtracted data is the result of fitting to the 14 T data.

S12 Calculation of theoretical maximum magnetic entropy change during ordering

To estimate the maximum possible magnetic entropy change associated with magnetic ordering in 12R-BNM, we consider the effective spin degrees of freedom present in the system. The three Mn^{4+} ions ($3d^3$, $S = \frac{3}{2}$) are assumed to form a magnetic cluster that behaves as a single spin-$\frac{3}{2}$ entity, with negligible angular momentum contribution. In contrast to 12R-$Ba_4CeMn_3O_{12}$, in 12R-BNM the lanthanoid cation will contribute magnetic entropy in both the Nd^{3+} ($4f^3$) and Nd^{4+} ($4f^2$) oxidation states, and so assuming free-ion paramagnetism will have a larger magnetic entropy. Also, in contrast to Mn^{4+}, angular momentum L cannot be discounted for Nd, resulting in a total angular momentum quantum number $J = 4$ and $J = \frac{9}{2}$ for Nd^{4+} and Nd^{3+} respectively, corresponding in turn to a 9-fold and 10-fold degeneracy.

The total degeneracy of the system is therefore the product of the individual degeneracies:

$$\Omega = (2S + 1)(2J + 1) = 4(2J + 1) \tag{S10}$$

Ω is therefore 36 or 40 for Nd^{4+} and Nd^{3+} respectively. The corresponding magnetic entropy change is given by:

$$\Delta S_{\text{mag}} = k_B \ln(\Omega) \tag{S11}$$

To express this in terms of $k_B \ln(2)$, a natural unit for magnetic entropy, we evaluate:

$$\frac{\Delta S_{\text{mag}}}{k_B \ln(2)} = \frac{k_B \ln(\Omega)}{k_B \ln(2)} = \frac{\ln(\Omega)}{\ln(2)} = \log_2(\Omega) \tag{S12}$$

Thus, the expected magnetic entropy release through ordering in $Ba_4Mn_3NdO_{12}$ is, assuming Nd^{4+}:

$$\Delta S_{\text{mag}} \approx 5.17\, k_B \ln(2) \tag{S13}$$

Assuming Nd is in the Nd^{3+} oxidation state, the entropy release is:

$$\Delta S_{\text{mag}} \approx 5.32\, k_B \ln(2) \tag{S14}$$

This value represents the theoretical maximum entropy loss assuming complete ordering of the spin degrees of freedom associated with the Mn_3O_{12} cluster and Nd^{4+} ion. Given that Nd is likely mixed between these oxidation states, the true theoretical maximum entropy is likely somewhere between these values.

S13 Magnetic form factor calculation

For the Rietveld refinement of the magnetic structure from neutron diffraction data, the magnetic form factor is modelled using TOPAS 7.[20] For a full description of the calculation, the documentation of TOPAS 7 should be consulted. Here, we note that the $\langle j_i(s)\rangle$ integrals within the form factor calculation were modelled via the 7-parameter analytical approach. In this approach, the $\langle j_0(s)\rangle$ and $\langle j_2(s)\rangle$ integrals are approximated thus:

$$\langle j_0(s)\rangle = A\exp(-as^2) + B\exp(-bs^2) + C\exp(-cs^2) + D \tag{S15}$$

$$\langle j_2(s)\rangle = \big(A\exp(-as^2) + B\exp(-bs^2) + C\exp(-cs^2) + D\big)\, s^2 \tag{S16}$$

In Equation S15 and S16, s is the spin of the magnetic ion and A, a, B, b, C, c, and D are empirical parameters unique for each oxidation state and element. The values of these parameters used in this work are given in Table S7, and taken from the Institut Laue-Langevin website here: https://www.ill.eu/sites/ccsl/ffacts/ffactnode7.html.

We are unaware of any reported form factors for Nd^{4+} magnetic cations, so in this work we have used the form factors for Nd^{3+} despite Nd being likely mixed 4+/3+. We believe this is reasonable as both oxidation states have $4f$ electrons responsible for their magnetism, and such electrons are very far from the nucleus and highly-shielded. We note that Regier *et al.*[2] also used the magnetic form factors of the 3+ cation for the magnetic Pr^{4+} ions

Table S7: The empirical parameters used in Equations S15 and S16 for the calculation of the magnetic form factor. Magnetic form factors are used in the Rietveld refinement[9] of the magnetic structure of 12R-$Ba_4NdMn_3O_{12-\gamma}$. The values are taken from the Institut Laue-Langevin website here: https://www.ill.eu/sites/ccsl/ffacts/ffactnode7.html.

Ion	$\langle j_i(s)\rangle$	A	a	B	b	C	c	D
Mn^4+	$\langle j_0(s)\rangle$	0.3760	12.5661	0.6602	5.1329	-0.0372	0.5630	0.0011
	$\langle j_2(s)\rangle$	0.7879	13.8857	1.8717	5.7433	0.5981	2.1818	0.0034
Nd^3+	$\langle j_0(s)\rangle$	0.0540	25.0293	0.3101	12.1020	0.6575	4.7223	-0.0216
	$\langle j_2(s)\rangle$	0.6751	18.3421	1.6272	7.2600	0.9644	2.6016	0.0150

in the Rietveld refinement of the magnetic structure of the Pr-containing analog of 12R-$Ba_4NdMn_3O_{12}$.

S14 Density Functional Theory

In the main text of this manuscript we showed DFT calculations of the Density-of-States (DOS) and oxygen vacancy formation energy. Calculations were performed as discussed in Methods in main text. Here we present in Figure S42 and Table S8 the oxygen vacancy formation energy, and in Figure S43 the full DOS for 12R-BNM, with comparison to 12R-$Ba_4PrMn_3O_{12}$, the Pr-containing analog, as reported by Regier *et al.*[2] Valuable future work would be to verify these trends *via* variable-temperature conductivity measurements on polycrystalline or, better yet, single crystal samples.

Table S8: DFT-calculated oxygen vacancy formation energies for 12R-$Ba_4LnMn_3O_{12}$ (Ln = Ce, Pr, Nd). The calculations in this table assume no O vacancies to begin with.

Compound	O1 (eV)	O2 (eV)	Mean (eV)
12R-$Ba_4CeMn_3O_{12}$	2.66	3.40	3.03
12R-$Ba_4PrMn_3O_{12}$	2.59	2.92	2.76
12R-$Ba_4NdMn_3O_{12}$	1.77	1.73	1.75

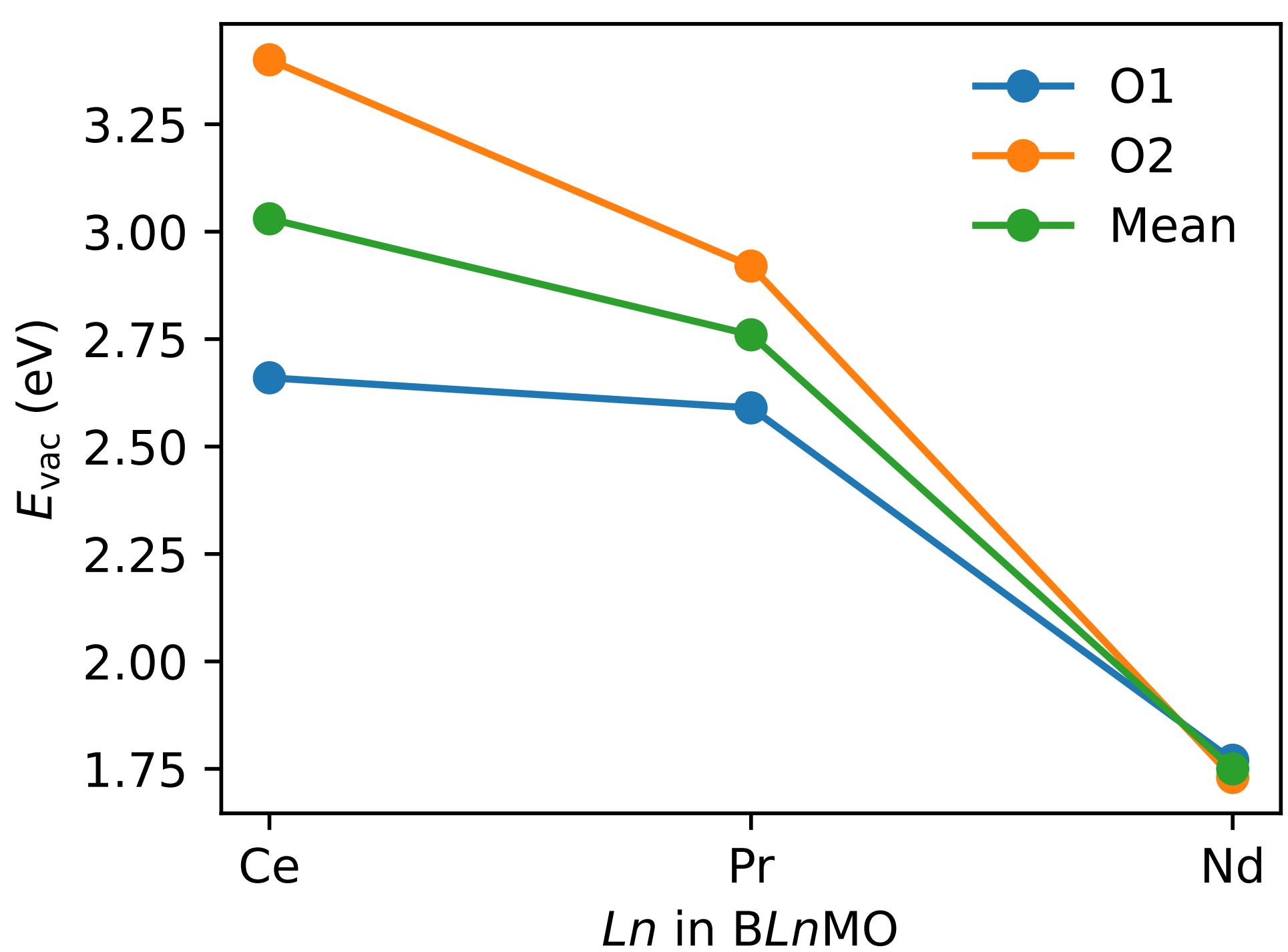

Figure S42: Oxygen vacancy formation energy from DFT+U calculations.

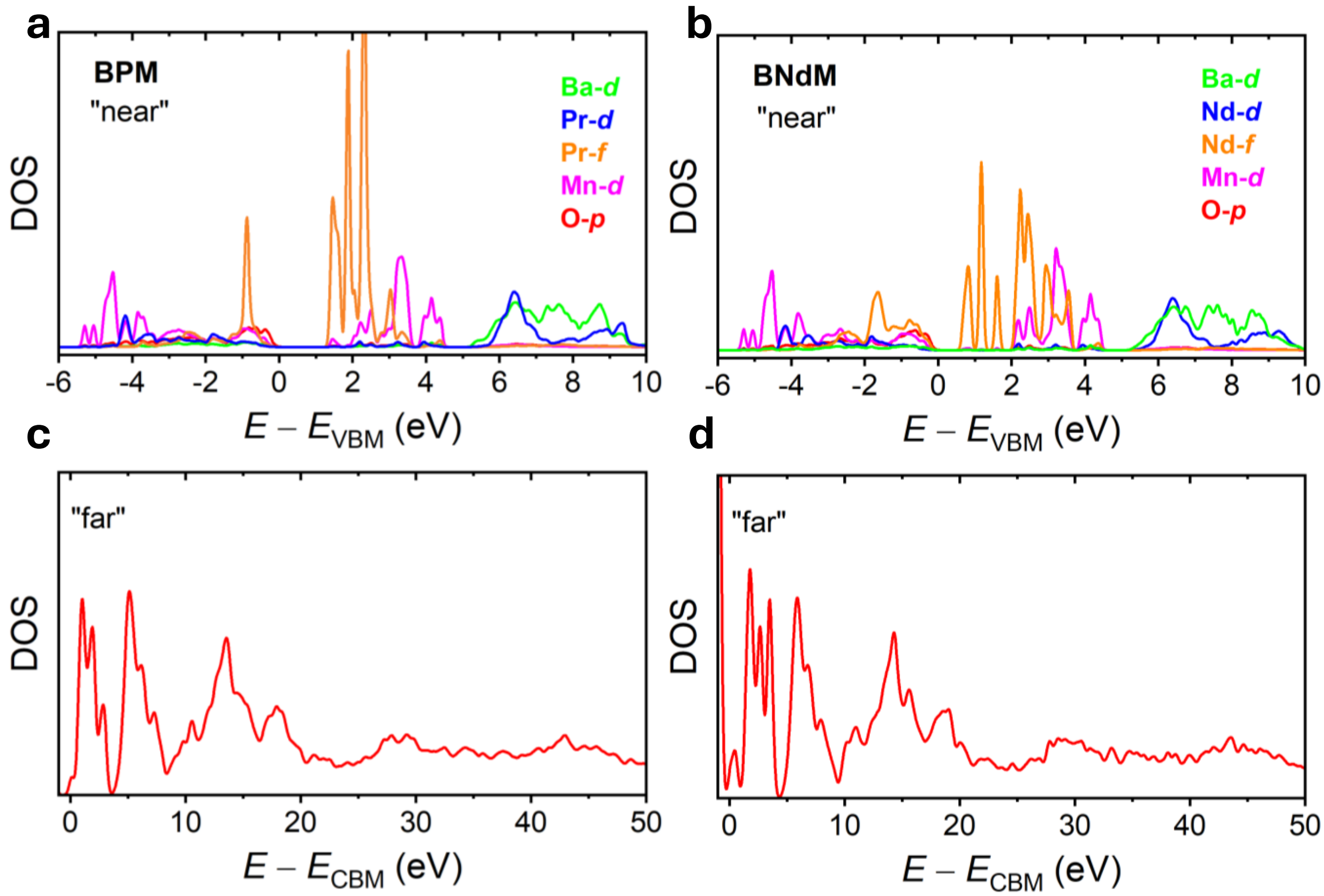

Figure S43: Density-of-States for 12R-$Ba_4Mn_3LnO_{12}$, where Ln is (a,c) Pr^{4+} and (b,d) Nd^{4+}. Calculations assume zero O vacancies. Data for Pr^{4+} are reproduced from Regier *et al.*[2] whereas the data for Nd^{4+} are calculated specifically for this paper. (a,b) shows the calculated partial DOS in the vicinity of the Fermi level, whereas (c,d) shows the O-p partial DOS up to 50 eV above the conduction band maximum. A Gaussian smoothing with σ of (a,b) 0.05 and (c,d) 0.20 eV was applied to the DOS.

References

(1) Dzara, M. J.; Campello, A. C.; Breidenbach, A. T.; Strange, N. A.; Park, J. E.; Ambrosini, A.; Coker, E. N.; Ginley, D. S.; Lee, Y. S.; Bell, R. T.; Smaha, R. W. Influence of the Rare Earth Cation on the Magnetic Properties of Layered 12R-$Ba_4M^{4+}Mn_3O_{12}$ (M = Ce, Pr) Perovskites. *Chemistry of Materials* **2024**, *36*, 2810–2818.

(2) Regier, C. E.; O'Donnell, S.; Goyal, A.; Dzara, M. J.; Park, J. E.; Bell, R. T.; Kramer, M. J.; Paddison, J. A. M.; Shulda, S.; Ginley, D. S.; Yahne, D. R.; Lany, S.; Smaha, R. W.; Klein, R. A. Interlayer Ions Control Spin Canting in Low-Dimensional Manganese Trimers in 12R-$Ba_4MMn_3O_{12}$ (M = Ce, Pr) Layered Perovskites. *Inorganic Chemistry* **2024**, *63*, 24176–24186.

(3) Panish, M. B. Vaporization of several rare earth oxides. *Journal of Chemical Physics (US)* **1961**, *34*.

(4) Bassou, A. A.; Machado, P. J.; Gomes, M. M.; Manjunath, B.; Vilarinho, R.; Silva, B.; Oliveira, J.; Almeida, B.; Almeida, A.; Fernandes, J. R.; others Thermal stability and decomposition kinetics of $NdNiO_{3-\delta}$ at 1 bar of O_2. *Materials Today Communications* **2021**, *28*, 102663.

(5) Key, T. S.; Crist Jr, B. Metastable States in Alumina-Rich La_2O_3:Al_2O_3 and Nd_2O_3:Al_2O_3. *Journal of the American Ceramic Society* **2005**, *88*, 191–195.

(6) Bi, S. L.; Wu, B. L.; Wu, Y. Interface Reaction between Nd_2O_3 and Alumina Single Crystal. *Advanced Materials Research* **2015**, *1104*, 21–25.

(7) Pawley, G. S. Unit-Cell Refinement From Powder Diffraction Scans. *J. Appl. Cryst* **1981**, *14*, 357–361.

(8) Iyi, N.; Inoue, Z.; Kimura, S. The crystal structure of neodymium hexaaluminate. *Journal of Solid State Chemistry* **1984**, *54*, 123–125.

(9) Rietveld, H. M. The Rietveld method. *Physica Scripta* **2014**, *89*, 098002.

(10) Pein, A.; Puhr, B.; Jones, A. *The Non-Ambient Guide: Principles of Non-Ambient X-Ray Diffraction*, 1st ed.; Anton Paar GmbH: Austria, 2018.

(11) Huq, A.; Kirkham, M.; Peterson, P. F.; Hodges, J. P.; Whitfield, P. S.; Page, K.; Hűgle, T.; Iverson, E. B.; Parizzi, A.; Rennich, G. POWGEN: rebuild of a third-generation powder diffractometer at the Spallation Neutron Source. *Applied Crystallography* **2019**, *52*, 1189–1201.

(12) Zubkov, V. G.; Tyutyunnik, A. P.; Berger, I. F.; Voronin, V. I.; Bazuev, G. V.; Moore, C. A.; Battle, P. D. Crystal and magnetic structures of $Ba_4Mn_3O_{10}$. *Journal of Solid State Chemistry* **2002**, *167*, 453–458.

(13) Litvin, D. B. Tables of crystallographic properties of magnetic space groups. *Foundations of Crystallography* **2008**, *64*, 419–424.

(14) Thole, B. T.; Van der Laan, G.; Fuggle, J. C.; Sawatzky, G. A.; Karnatak, R. C.; Esteva, J.-M. 3d x-ray-absorption lines and the $3d^94f^{n+1}$ multiplets of the lanthanides. *Physical Review B* **1985**, *32*, 5107.

(15) Suljoti, E.; Nagasono, M.; Pietzsch, A.; Hickmann, K.; Trots, D. M.; Haase, M.; Wurth, W.; Föhlisch, A. Geometric and electronic structure of lanthanide orthophosphate nanoparticles determined with x-rays. *The Journal of Chemical Physics* **2008**, *128*.

(16) Tripathi, S. XMCD investigation at $M_{4,5}$ edges of the rare earth elements in high-performance permanent magnet. PhD thesis, University of Stuttgart, Stuttgart, Germany, 2018.

(17) Adkin, J. J.; Hayward, M. A. Structure and magnetism of 4H-$BaMnO_{3-x}$ ($0 \leq x \leq 0.35$)

and 4H-$Ba_{0.5}Sr_{0.5}MnO_{3-x}$ ($0 \leq x \leq 0.21$). *Journal of Solid State Chemistry* **2006**, *179*, 70–76.

(18) Nguyen, L. T.; Kong, T.; Cava, R. J. Trimers of MnO_6 octahedra and ferrimagnetism of $Ba_4NbMn_3O_{12}$. *Materials Research Express* **2019**, *6*.

(19) Debye, P. Zur theorie der spezifischen wärmen. *Annalen der Physik* **1912**, *344*, 789–839.

(20) Coelho, A. A. TOPAS and TOPAS-Academic: An optimization program integrating computer algebra and crystallographic objects written in C++. *Journal of Applied Crystallography* **2018**, *51*, 210–218.
